# Experimental Quantification of Spin–Phonon Coupling in Molecular Qubits Using Inelastic Neutron Scattering

**DOI:** 10.1021/jacs.6c03700

**Published:** 2026-05-08

**Authors:** Stefan H. Lohaus, Kay T. Xia, Yongqiang Cheng, Ryan G. Hadt

**Affiliations:** † Division of Chemistry and Chemical Engineering, Arthur Amos Noyes Laboratory of Chemical Physics, 6469 California Institute of Technology, Pasadena, California 91125, United States; ‡ Neutron Scattering Division, 6146 Oak Ridge National Laboratory, Oak Ridge, Tennessee 37831, United States

## Abstract

Electronic spin superposition states enable nanoscale sensing through their sensitivity to the local environment, yet their sensitivity to vibrational motion also limits their coherence times. In molecular spin systems, chemical tunability and atomic-scale resolution are accompanied by a dense, thermally accessible phonon spectrum that introduces efficient spin relaxation pathways. Despite extensive theoretical work, there is little experimental consensus on which vibrational energies dominate spin relaxation or how molecular structure controls spin–phonon coupling (SPC). We present a fully experimental method to quantify SPC coefficients by combining temperature-dependent vibrational spectra from inelastic neutron scattering with spin relaxation rates measured by electron paramagnetic resonance. We apply this framework to two model *S* = 1/2 systems, copper­(II) phthalocyanine (CuPc) and copper­(II) octaethylporphyrin (CuOEP). Two distinct relaxation regimes emerge: below 40 K, weakly coupled lattice modes below 50 cm^–1^ dominate, whereas above 40 K, optical phonons above ∼185 cm^–1^ become thermally populated and drive relaxation with SPC coefficients nearly 3 orders of magnitude larger. Structural distortions in CuOEP that break planar symmetry soften the crystal lattice and enhance anharmonic scattering but also raise the energy of stretching modes at the molecular core where the spins reside. This redistributes vibrational energy toward the molecular periphery and out of plane, ultimately reducing SPC relative to CuPc and enabling room-temperature spin coherence in CuOEP. Although our method does not provide mode-specific SPC coefficients, it quantifies contributions from distinct spectral regions and establishes a broadly applicable, fully experimental link between crystal structure, lattice dynamics, and spin relaxation.

## Graphical abstract

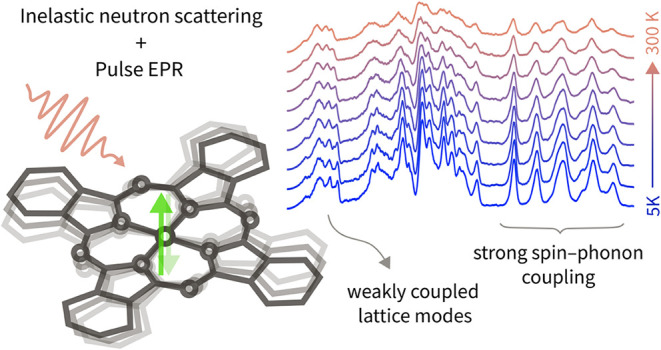

## Introduction

The application of quantum bits (qubits) as nanoscale sensors has enabled measurements at unprecedented spatial and sensitivity scales, from detecting single-neuron action potentials^1^ and mapping subcellular temperature variations,^2^ to single-protein NMR spectroscopy.^3^ These experiments were achieved by creating superpositions of spin states and exploiting their high sensitivity to the environment. While record room-temperature coherence times of up to 1.8 ms were achieved for spins housed at nitrogen-vacancy defects in a diamond lattice,^4^ their bulky carbon framework restricts chemical tunability and limits the spatial resolution to several nanometers. An alternative molecular approach uses unpaired electron spins of paramagnetic complexes. In addition to enabling further miniaturization of quantum sensors, molecules are compatible with a range of physical and biological environments^5^ and have highly tunable structures, enabling chemists to adjust their electronic transitions and vibrational spectra.^6–8^

For quantum sensors, spins must respond sensitively to external signals while remaining well shielded from environmental noise to preserve long coherence times.^9^ This has been achieved by minimizing nearby electronic and nuclear spins that induce decoherence through dipole–dipole and hyperfine interactions.^4,10^ Interactions with lattice vibrations, however, are unavoidable and become increasingly significant as more phonon modes are thermally populated. At room temperature, spin–phonon coupling (SPC) becomes a dominant relaxation channel, limiting coherence and destroying spin superposition.^11^ As discussed below, many recent studies have modeled spin relaxation using *ab initio* and ligand-field frameworks to pinpoint the most strongly coupled modes. Yet, predictions remain inconsistent, and experimental quantification of individual coupling coefficients has not been achieved.

Here, we take a fully experimental approach, measuring vibrational spectra by inelastic neutron scattering (INS) and correlating them to the spin relaxation determined by pulse electron paramagnetic resonance (EPR). We chose copper­(II) phthalocyanine (CuPc) and copper­(II) octaethylporphyrin (CuOEP), two model systems with one unpaired electron at their Cu­(II) centers (*S* = 1/2), see Figure 1a,b. In a magnetic field, the spin sublevels split and form a two-level system that can be manipulated by microwave pulses in an EPR spectrometer. Interactions with lattice vibrations drive relaxation back to equilibrium, with a characteristic time scale defined as the spin–lattice relaxation time *T*_1_.

**Figure 1:**
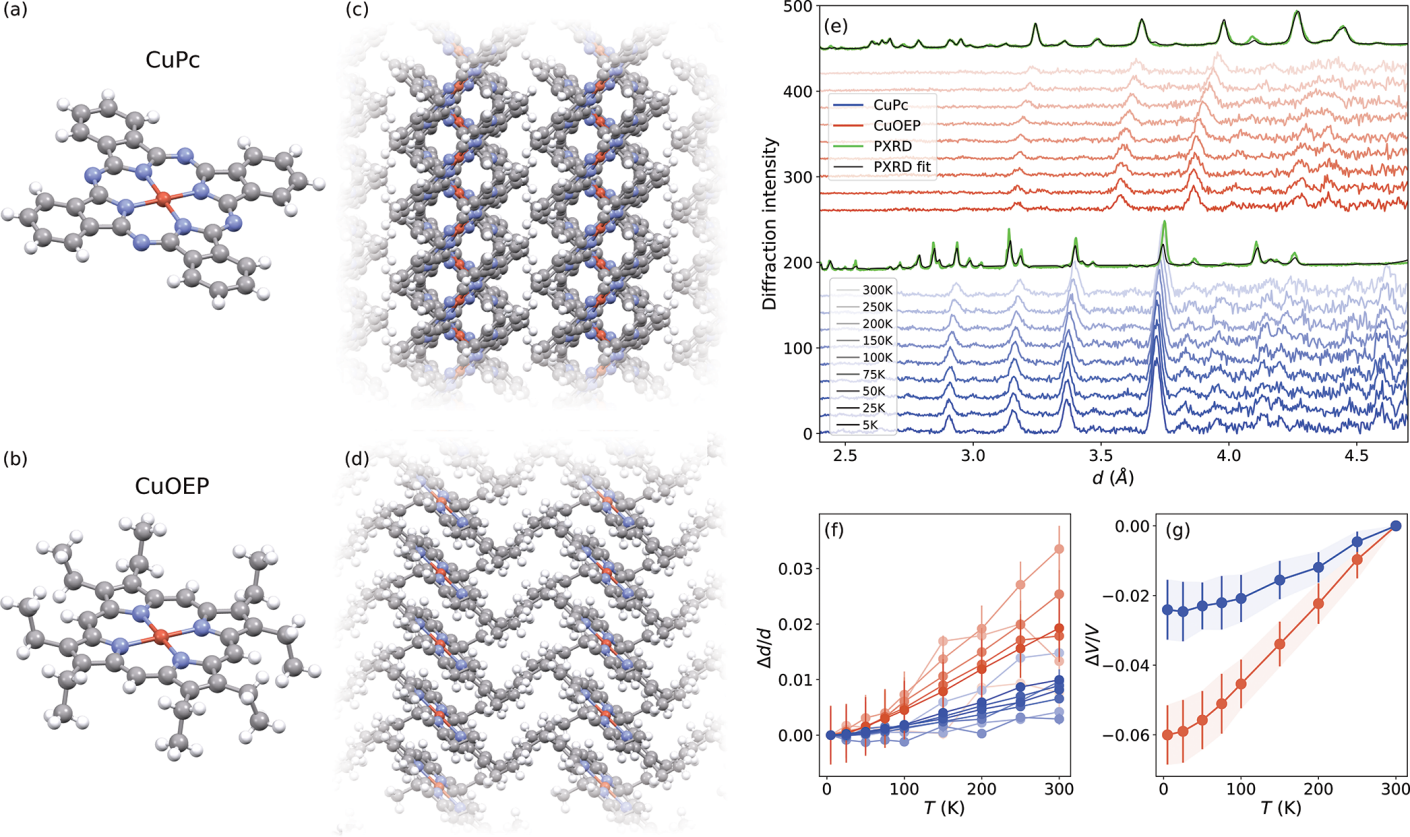
Structural characterization. Molecular (a, b) and crystal structures (c, d) of CuPc and CuOEP. (e) Neutron diffraction patterns collected between 5 and 300 K (dark to light shades) compared to PXRD at 300 K. (f) Temperature-dependent *d*-spacing changes along different crystallographic directions (different shades), obtained from fits to the peaks in panel (e), with error bars representing one-sigma fitting uncertainties. (g) Fractional volume change assuming an isotropic lattice expansion (see SI Section 2). Errors indicate the standard deviation between the isotropic prediction and the measured anisotropic *d*-spacings.

Many studies have sought to identify which phonon modes most strongly contribute to spin relaxation in *S* = 1/2 systems. Ligand-field models predict that high-energy optical modes involving symmetric metal–ligand stretching have the symmetry needed to produce the largest variations in the spin sublevel splitting (*g* value).^12–14^ Raman spectroscopy has indeed linked such symmetric stretches (200–300 cm^–1^) to spin relaxation above 40 K in Cu­(II) porphyrins.^15^ When molecular symmetry is lowered, this selection rule relaxes, enabling additional vibrational modes to couple linearly with the spin.^14^

Beyond these symmetry-based models, *ab initio* calculations remain divided and at times contradictory about which vibrations govern relaxation. One study reports comparable coupling strengths across both low- and high-energy modes,^16^ while others attribute the strongest effects either to the lowest optical phonons below 50 cm^–1^ ^17,18^ or to modes above 200 cm^–1^.^19^ Single-crystal EPR measurements on Cu­(acac)_2_ show that both high-energy optical and low-energy lattice vibrations contribute to spin relaxation, but in different temperature regimes. As temperature increases, the dominant relaxation pathway shifts from collective lattice modes to intramolecular optical vibrations.^20^

Another key factor is the mixing between vibrational modes of optical and acoustic character. Acoustic modes with long wavelengths barely distort individual molecules and cannot directly couple to spins.^21^ However, they can contribute to spin relaxation when mixed with intramolecular modes.^22,23^ This understanding motivated design strategies that reduce the number of low-energy modes and use rigid ligands to reduce the motion of the molecular core,^23–25^ as well as approaches that combine high-energy intramolecular modes with weak intermolecular interactions to limit coupling between optical and acoustic modes.^26^

Although phonons lie at the heart of spin–lattice relaxation, direct experimental observation of their spectra in *S* = 1/2 molecules remains remarkably scarce. Optical spectroscopies (Raman, IR, THz) are bound to symmetry-based selection rules and access only zone-centers. To date, phonon dispersions have been experimentally mapped in only two systems: VO­(acac)_2_ by INS^22^ and VO­(TPP) by inelastic X-ray scattering (IXS).^27^ These studies highlighted the importance of low-energy modes in spin relaxation, revealing optical vibrations as low as 10 cm^–1^ arising from the softness of the porphyrin lattice. However, both techniques are experimentally demanding and involve complex data reduction, hindering systematic, temperature-dependent studies across molecular systems. For example, single-crystal INS requires large, well-aligned molecular crystals and laborious sample rotation, while IXS measurements can be limited by X-ray radiation damage.

Using an alternative instrument design, we employ INS to directly probe the full vibrational spectrum of *S* = 1/2 complexes up to 8000 cm^–1^ (the highest-energy phonon feature appears at 3100 cm^–1^). The inverted geometry of the VISION spectrometer at the Oak Ridge National Laboratory^28,29^ integrates over momentum, sacrificing momentum resolution in exchange for rapid acquisition of complete spectra. Each spectrum was measured in under 30 min using ∼500 mg of powder sample. This capability enables temperature-dependent measurements between 5 and 300 K, capturing anharmonicity through line broadening and frequency shifts. By combining these vibrational spectra with pulse EPR measurements of spin–lattice relaxation, we propose a fully experimental framework to interpret SPC. This approach identifies the spectral regions most relevant to spin relaxation and provides, for the first time, experimental SPC coefficients for low- and high-energy modes, bridging the gap between theory-driven models and experiment.

## Results and Discussion

### Molecular and Crystal Structures

CuPc is a planar molecule with four coordinating nitrogens and a ligand composed of fused pyrrole and benzene rings, forming a D_4h_ framework (Figure 1a). CuOEP has the same CuN_4_ coordination, but the peripheral benzene rings are opened into β-ethyl groups that protrude out of the plane (Figure 1b). This results in a saddled macrocycle with a square planar core.^15,30^

These structural differences result in different packing in the crystalline phase and different space groups (Figure 1c,d). CuPc typically crystallizes in its monoclinic β-phase, with two molecules per unit cell arranged in a herringbone-like stacking (criss-cross) along the b direction, with an interlayer spacing of 3.34 Å. In contrast, CuOEP crystallizes in a triclinic structure. It adopts a simpler stacking with one molecule per unit cell and an interlayer spacing of 3.33 Å. Interactions between layers are mediated by the out-of-plane β-ethyl groups and by π–π contacts.

The lattice structures of CuPc and CuOEP were characterized by X-ray and neutron diffraction (Figure 1e). Their distinct powder X-ray diffraction (PXRD) patterns (green curves) were indexed and Rietveld-refined against the corresponding crystal structures shown in Figure 1c,d using GSAS-II (black curves). *In situ* neutron diffraction was used to quantify thermal expansion in both compounds. Although only a few Bragg reflections are visible due to strong incoherent scattering from hydrogen, peak positions in *d*-space could be tracked by Gaussian fits (Figure 1f). The change in lattice volume was determined, assuming isotropic thermal expansion, using PXRD values measured at room temperature as references (Figure 1g). For CuPc, a full monoclinic fit yielded a volume expansion equivalent to the isotropic model (see SI Section 2). CuOEP exhibits more than twice the expansion of CuPc, reflecting its softer lattice arising from the out-of-plane β-ethyl groups that weaken intermolecular interactions. All fits, models, and peak indexing details are provided in Section 2 of the Supporting Information.

### Spin–Lattice Relaxation

Spin–lattice relaxation rates (1/*T*_1_) of CuPc and CuOEP, measured by pulse EPR between 3.5 and 300 K, are shown in Figure 2a. To minimize spin–spin interactions and isolate intrinsic spin–lattice relaxation, powder samples were diluted into isostructural diamagnetic matrices (ZnPc and ZnOEP) at a 1:1000 molar ratio. In terms of phonons, Cu–Zn substitution is expected to have only a modest effect on long-wavelength, collective lattice modes, as the two compounds are structurally very similar (with lattice parameters changing by at most 1.24% for Pc and 2.41% for OEP; see SI Section 1) and differ by only ∼0.3% in total molecular mass. Modes with stronger metal–ligand participation may exhibit more noticeable energy shifts.

**Figure 2:**
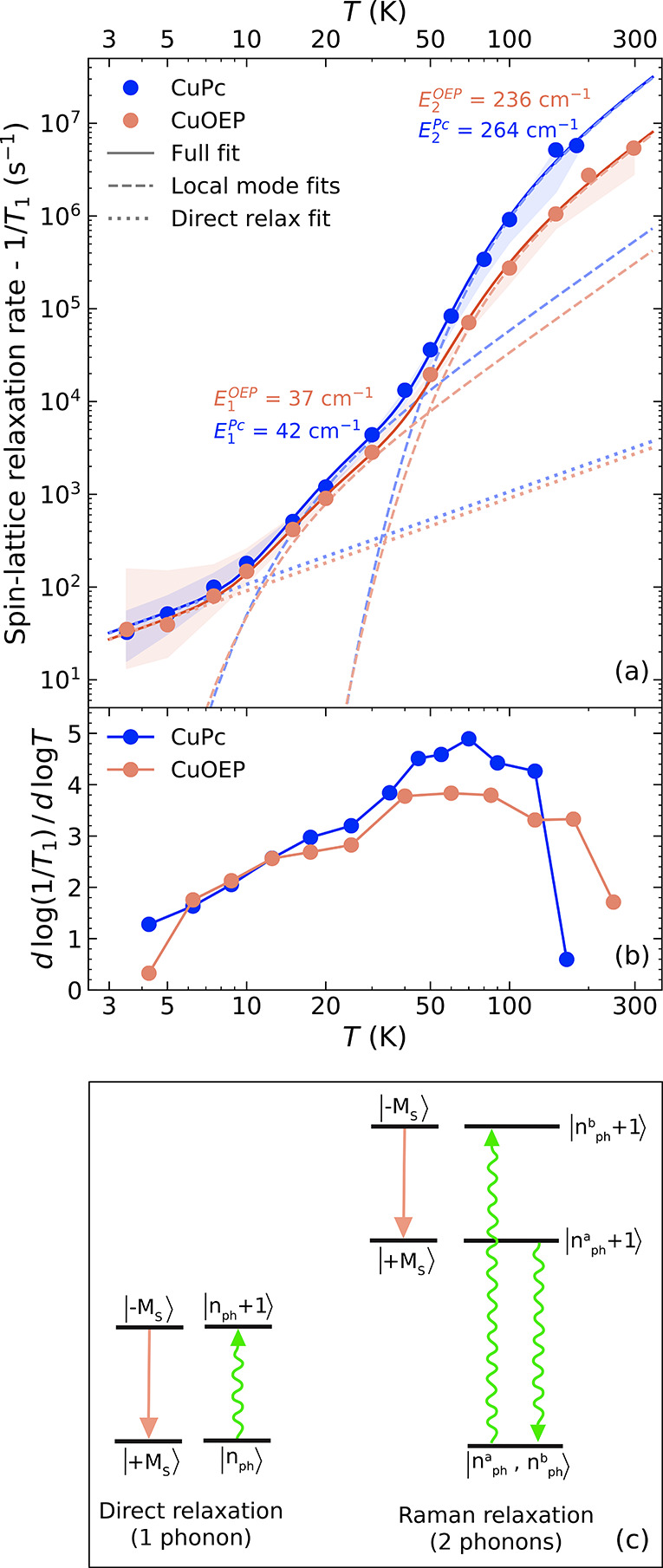
*S*pin dynamics characterization. (a) Spin–lattice relaxation rates (1/*T*_1_) of CuPc and CuOEP measured by pulse EPR at perpendicular field orientations. Data were collected by saturation recovery below 30 K and inversion recovery above. The shaded areas indicate the full range of data acquired at parallel and perpendicular field orientations, with both inversion and saturation recovery experiments (see SI Section 3). Dashed lines represent local-mode fits using eq 1, dotted lines show the one-phonon contributions, and solid curves the combined fit. (b) Logarithmic slopes of the data from panel (a), giving the power-law exponent in the temperature dependence of 1/*T*_1_. (c) Schematic illustration of spin relaxation mechanisms involving one-phonon (direct) and two-phonon (Raman) processes.

EPR data were collected using inversion- and saturation-recovery sequences at magnetic field positions corresponding to molecular orientations parallel and perpendicular to the applied field. Spectral diffusion, which is minimized in saturation-recovery measurements, is noticeable only at low temperatures. Accordingly, Figure 2 presents saturation-recovery data below 30 K and inversion–recovery data at higher temperatures (for the perpendicular field orientation). The complete data set is shown in Figure 5 and discussed in Section 3 of the SI. Our measurements are consistent with previous pulse EPR studies at high temperatures^11,15^ and extend those results to lower temperatures with improved temperature resolution.

As temperature increases, phonon populations rise and spin relaxation accelerates. Both molecules exhibit comparable relaxation rates at low temperature, but at elevated temperatures CuPc relaxes more rapidly. By 180 K, its excited-spin lifetime becomes too short to detect by pulse EPR, whereas CuOEP remains coherent up to room temperature (*T*_1_ for CuOEP at 300 K was taken from the 1:100 dilution data,^15^ as the 1:1000 sample signal was too weak). Changes in the slope of 1/*T*_1_(*T*) indicate the onset of additional relaxation mechanisms (Figure 2b shows the logarithmic derivative of panel a). Two such transitions appear near 7 and 40 K. Below 7 K, the nearly linear dependence (1/*T*_1_ ∝ *T*) corresponds to the *direct* process, in which a spin relaxes by emitting a single phonon of matching energy (see Figure 2c). Both CuPc and CuOEP exhibit comparable contributions from this process. The direct pathway is inefficient, however, because the phonon density is extremely low at the spin-transition energy (∼0.14 cm^–1^ at 0.3 T).

Two-phonon Raman processes dominate spin relaxation above 7 K. In this mechanism, one phonon is absorbed while another is emitted to mediate the spin transition (Figure 2c). Only the energy difference between the two phonons must match the spin splitting, allowing the entire thermally accessible phonon spectrum to contribute. Using the Bose–Einstein distribution $n\left(E,T\right)=1/\left(e^{E/k_{B}T}-1\right)$ , which gives the thermal population of phonon modes, the probability of such a two-phonon relaxation process becomes^18^

1 $${\left(\frac{1}{T_{1}}\right)}_{2-ph}\propto n\left(E_{ph},T\right)\times \left(n\left(E_{ph},T\right)+1\right)=\frac{e^{E_{ph}/k_{B}T}}{{\left(e^{E_{ph}/k_{B}T}-1\right)}^{2}}$$

This *local-mode model* has the same *T* ^2^ high-temperature limit as the widely used Debye model. In contrast, the local-mode model allows direct extraction of the phonon energies involved in spin relaxation and remains applicable across all energies beyond the Debye (quadratic) phonon regime, which extends only up to ∼15 cm^–1^ in molecular systems. Alternative Debye fits are provided in Section 3 of the SI.

The relaxation data in Figure 2a were fitted using two local modes in addition to the linear direct process. Relaxation between 7 and 40 K is mediated by phonons around 42.5 ± 5.7 cm^–1^ for CuPc and 37.1 ± 4.8 cm^–1^ for CuOEP, while the high-temperature regime involves modes around 264.8 ± 14.4 cm^–1^ and 236.2 ± 12.9 cm^–1^, respectively (errors denote one-sigma fit uncertainties). Without direct phonon measurements, spin-relaxation fits can only identify these characteristic vibrational energies. In the following section, we show how experimental phonon data provide a more complete picture of spin–lattice relaxation and enable quantification of coupling strengths.

### Phonon Spectra

We employ INS to measure the full vibrational spectra of CuPc and CuOEP between 5 and 300 K (Figure 3a). The VISION spectrometer measures the vibrational modes along a fixed momentum-energy trajectory, providing rapid access to the vibrational spectrum but not a strict phonon density of states (DOS) without computational input (details in SI Section 4.6). Because neutron scattering weights different atoms differently, the measured intensities can differ from the true DOS and are often dominated by hydrogen motion in hydrogen-rich materials. In the energy range relevant here, however, the vibrations involve motion of the whole molecule, so the spectra still capture the overall vibrational behavior, but with neutron-weighted intensities.

**Figure 3:**
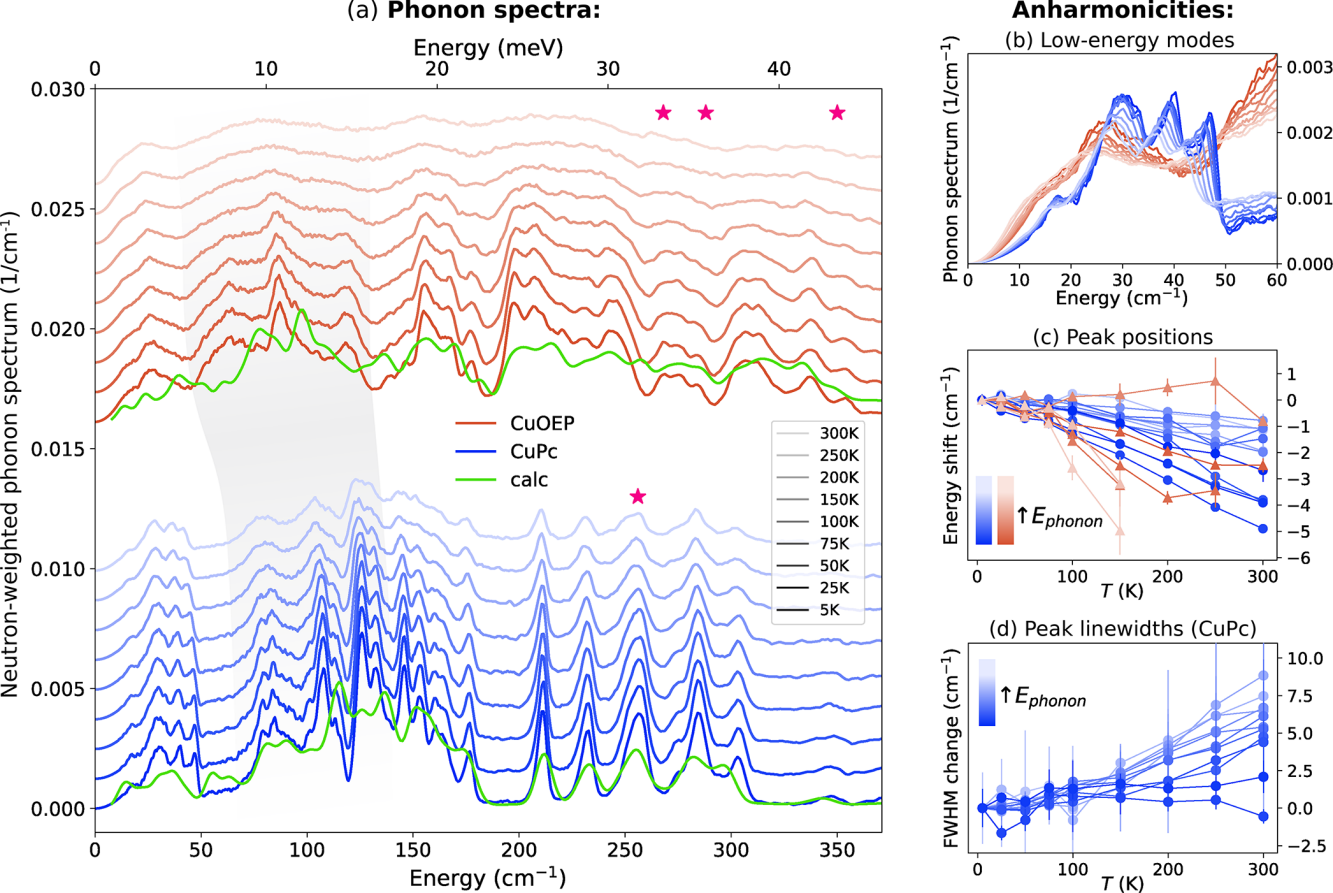
Phonon behavior of CuPc and CuOEP. (a) Normalized phonon spectra measured by INS between 5 and 300 K (after corrections described in Section 4 of the SI). Green curves show DFT-calculated phonon spectra. Stars mark the calculated energy of the symmetric stretch of CuPc and the three modes of CuOEP with the largest symmetric stretching character. Gray shading visualizes the red-shifted spectrum of CuOEP compared to CuPc. (b) Expanded view of the low-energy phonon region. (c) Anharmonic energy shifts of CuPc (blue) and CuOEP (orange), with lighter shades corresponding to higher phonon energies. Error bars show one-sigma peak fitting uncertainties. (d) Temperature-dependent phonon broadening of CuPc, quantified as changes in FWHM from Gaussian fits.

To minimize computational input, we use the measured VISION spectra in the analysis and then compare the results with those obtained using the phonon DOS. All spectra were corrected for excitation probabilities (Bose correction), multiphonon excitations, and instrumental background (see SI Section 4 for details and the full data set). The spectra are normalized to unity up to 600 cm^–1^, which we take here as the upper limit of thermal accessibility at 300 K. The DFT-calculated phonon spectra (see SI Section 8), shown in green, reproduce most of the measured features and are used to assign the character of the observed modes (*vide infra*).

CuPc exhibits sharp phonon modes up to room temperature and a distinct set of low-energy translational and bending modes below about 50 cm^–1^ (Figure 3b). These include acoustic lattice phonons and low-energy optical vibrations, often referred to as pseudoacoustic modes. The quadratic energy dependence below 10–15 cm^–1^ arises from the Debye model used to remove the experimental elastic line. Between 70 and 200 cm^–1^, CuPc shows higher-order bending motions such as doming, saddling, and ruffling, together with an asymmetric stretch near 175 cm^–1^. Above 200 cm^–1^, modes include in-plane torsion (212 cm^–1^), scissoring (229 cm^–1^), and the symmetric stretch (256 cm^–1^, marked with a star), which is often considered central to spin relaxation. The mode assignments for both CuPc and CuOEP agree with previous calculations and resonance Raman measurements.^14,15,31,32^

CuOEP has a similar phonon spectrum, but with substantially broader line widths and a nearly featureless spectrum at room temperature. Much of its spectrum is shifted to lower energies compared to CuPc. While modes below 50 cm^–1^ are red-shifted by about 5 cm^–1^ with respect to CuPc, modes between 50 and 130 cm^–1^ have larger shifts of up to 20 cm^–1^ (visualized by the gray band in Figure 3a). These correspond to different bending modes, which are especially sensitive to the softer peripheral β-ethyl groups compared to the planar benzene rings of CuPc.

The calculations reveal a second important difference: the normal modes of CuOEP show much stronger vibrational mixing, combining in- and out-of-plane motion. In CuPc, the local D_4h_ symmetry keeps stretching and out-of-plane modes largely separable, whereas in CuOEP the saddled macrocycle (∼D_2d_) lifts these degeneracies and allows eigenvectors to mix. As a result, CuOEP exhibits several mixed stretching modes with substantial out-of-plane character, rather than a single dominant stretching mode seen in CuPc. In contrast to the lower-energy modes, which are red-shifted relative to CuPc, the dominant CuOEP stretching modes occur at higher energies (268, 288, and 350 cm^–1^; marked with stars in Figure 3a). This indicates a stiffer CuOEP molecular core relative to CuPc, alongside softer out-of-plane modes arising from its molecular geometry. This picture is consistent with prior DFT and resonance Raman studies.^15^ Such a local molecular symmetry description is very helpful as a simplifying picture, although the phonon eigenvectors ultimately reflect the full crystal symmetry of the monoclinic and triclinic lattices.

### Phonon Anharmonicities

With increasing temperature, the phonon spectra of CuPc and CuOEP show both energy shifts (softening to lower energies) and line width broadening. These reveal clear deviations from purely harmonic behavior. A useful starting point is the *quasiharmonic* model, in which phonon frequencies shift due to thermal expansion of the lattice and the associated weakening of restoring forces. In this framework, the energy shift of a phonon mode is described by its Grüneisen parameter γ_i_:

2 $$\frac{\Delta E_{i}}{E_{i}}=-\gamma_{i}\frac{\Delta V}{V}$$

Additional energy shifts beyond this model, as well as line width broadening, arise from higher-order anharmonic interactions. These give phonons finite lifetimes through processes such as phonon–phonon and spin–phonon scattering.

To quantify these effects in CuPc and CuOEP, phonon peaks were fitted with Gaussian and Voigt profiles (see SI Section 6 for details). In CuPc (Figure 3c,d), low-energy modes (<150 cm^–1^) soften by up to ∼ 5 cm^–1^ at 300 K with negligible line width changes, whereas higher-energy optical modes (>200 cm^–1^) remain nearly fixed in energy but broaden significantly, indicating shorter lifetimes. In CuOEP, strong spectral overlap prevents reliable fits to individual modes, so trends are discussed qualitatively. However, both low- and high-energy modes show pronounced softening (particularly the feature near 240 cm^–1^), suggesting stronger anharmonicity.

Thermal expansion measured by *in situ* neutron diffraction (Figure 1g) was used to model these frequency shifts through the quasiharmonic relation (eq 2). In both systems, the softening of low-energy modes scales approximately linearly with lattice expansion, indicating that Grüneisen parameters γ_i_ capture most temperature dependence (see Figures S42, S45). At low energies, γ_i_ is significantly larger for CuPc (*γ_i_* ≈ 4) than CuOEP (*γ_i_* ≈ 2). Since CuPc and CuOEP show comparable absolute frequency shifts in this range, but CuOEP expands considerably more, its vibrational spectrum is less sensitive to lattice expansion. The β-ethyl substituents of CuOEP weaken intermolecular interactions, softening the lattice and partially decoupling its vibrational modes from lattice strain. This greater vibrational isolation in CuOEP could contribute to its reduced 1/*T*_1_ compared to CuPc.

Line width broadening reflects reduced phonon lifetimes and is observed in both systems at elevated temperature, but is substantially more pronounced and widespread in CuOEP than in CuPc. Because both samples formed good crystals (Figure 1e), disorder is unlikely to be the origin of the enhanced line width. Instead, the lower symmetry and mixed character of CuOEP’s eigenvectors may facilitate additional phonon–phonon scattering channels not accessible in higher-symmetry structures.^33,34^ This may also contribute to the enhanced softening of its high-energy modes relative to CuPc. In particular, mixing of localized stretching modes with out-of-plane motion could transfer the typically stronger volume dependence of bending modes (higher γ_i_) to stretching ones.

### Thermal Phonon Population

Beyond vibrational energies and anharmonicities, understanding phonon behavior requires consideration of their thermal population. This is directly relevant for spin–lattice relaxation, since Raman processes require thermally populated phonon modes. The phonon mode population at each temperature can be visualized by weighting the phonon spectrum by the Bose–Einstein factor *n­(E,T)*, as shown in Figure 4.

**Figure 4:**
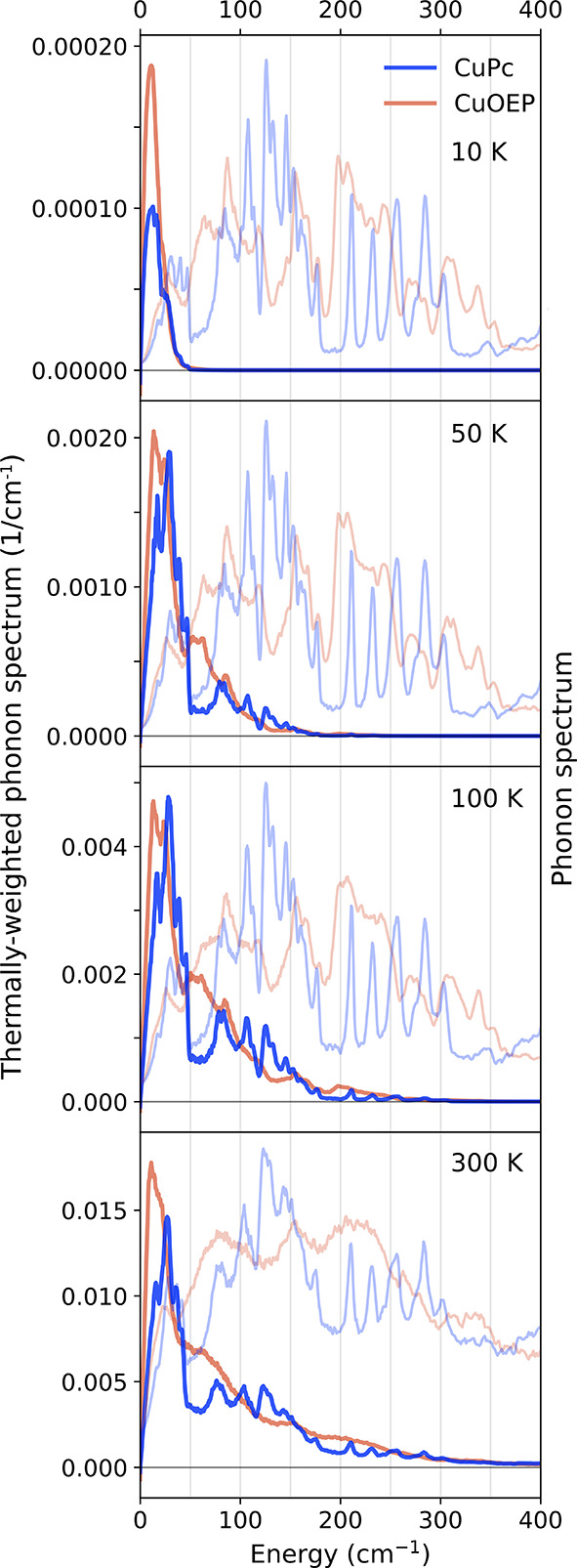
Thermal phonon population of CuPc and CuOEP. Bose-weighted phonon spectra at different temperatures, overlaid with their measured phonon spectra for reference (right axis, light-shaded curves). The spectra at 10 K are linear interpolations between 5 and 25 K measurements.

Below 10 K, CuOEP has a larger thermal phonon population than CuPc because its phonon modes below 25 cm^–1^ lie at lower energies. Around 10 K, modes around 40–50 cm^–1^ begin to populate, consistent with the onset of Raman relaxation through modes at 42 cm^–1^ and 37 cm^–1^ for CuPc and CuOEP, respectively, as identified by the local-mode fits in Figure 2a. By 50 K, all modes below 200 cm^–1^ are populated. The absence of new relaxation channels between 10 and 50 K suggests that modes between 50 and 200 cm^–1^ contribute little to spin relaxation. Beyond 50 K, phonons above 200 cm^–1^ become populated. The rapid increase in spin relaxation above 50 K indicates that they play a dominant role at higher temperatures, implying stronger coupling strengths.

### Spin–Phonon Coupling

To go beyond a qualitative discussion of SPC, we extend the local mode fitting model of eq 1 by explicitly incorporating the measured phonon spectra, *G*_*T*_ *(E)*. This reformulates the relaxation rate 1/*T*_1_ as an integral over the full vibrational spectrum, rather than in terms of discrete phonon energies:

3 $${\left(\frac{1}{T_{1}}\right)}_{T}=\int_{0}^{E_{ph,max}}\lambda_{SPC}\left(\varepsilon \right)\times G_{T}\left(\varepsilon \right)\times \frac{e^{\varepsilon /k_{B}T}}{{\left(e^{\varepsilon /k_{B}T}-1\right)}^{2}}d\varepsilon$$

Here *G*_*T*_(*E*) is normalized to one and *E*_*ph*,*max*_ = 600 cm^–1^. The effective spin–phonon coupling *λ*_*SPC*_(*E*) acts as a weighting function that is fitted to map the measured phonon spectrum onto the measured relaxation rates. We assume *λ_SPC_* (*E*) to be temperature-independent (it is, at most, weakly dependent on temperature^19^). To avoid overfitting, we exclude direct relaxation and fit only data above 10 K.

The main goal is to compare the contributions of highly populated low-energy vibrations to those of strongly coupled optical modes. A fully energy-resolved fitting function *λ*_SPC_(*E*) would overparameterize the model, so we approximate the SPC as constants over two spectral regions. Guided by the two dominant relaxation contributions identified from the EPR analysis in Figure 2a, we define:

$$\lambda_{SPC}\left(E\right)=\{\begin{array}{l} \lambda_{SPC}^{low},E\leq E_{trans}\\ \lambda_{SPC}^{high},E>E_{trans} \end{array}$$

Figure 5a–b shows the fits of eq 3 using these two coefficients, yielding $\lambda_{SPC}^{low}$ = 0.068 μs^–1^ and $\lambda_{SPC}^{high}$ = 127 μs^–1^ for CuPc, and $\lambda_{SPC}^{low}$ = 0.033 μs^–1^ and $\lambda_{SPC}^{high}$ = 20 μs^–1^ for CuOEP. In both molecules, the low-energy coupling is nearly 3 orders of magnitude smaller than the high-energy coupling. An alternative fit with a smoother transition of the SPC coefficient between both regions, along with the effects of field orientation and measurement method (inversion- vs saturation-recovery) on the fitted parameters, is discussed in Section 7 of the SI.

The transition energy separating the low- and high-energy regions was determined by minimizing the logarithmic root-mean-squared fitting error (Figure 5c). A value of *E*_trans_ = 185 cm^–1^ lies near a common minimum and coincides with a low-intensity spectral gap for both CuPc and CuOEP, providing a natural boundary between the two regimes. The thermal crossover between these regions occurs at 42 K for CuPc and 45 K for CuOEP. For CuOEP, this closely matches the 47 K crossover identified by pulse EPR anisotropy measurements,^20^ which marks the transition from lattice-dominated to more localized relaxation processes.

**Figure 5:**
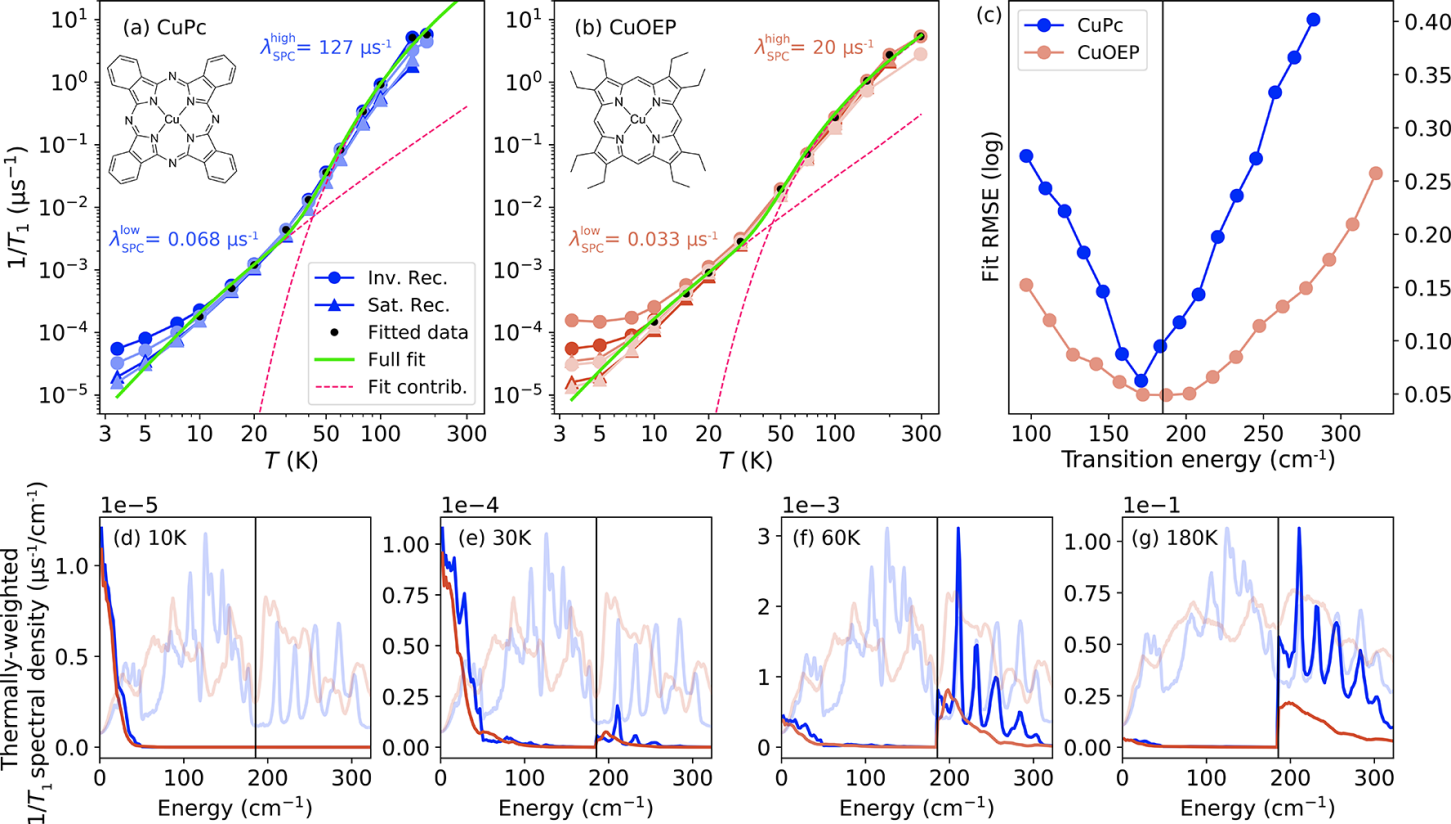
Spin–phonon coupling in CuPc and CuOEP. (a, b) Spin–lattice relaxation of CuPc and CuOEP measured by pulse EPR using inversion- and saturation-recovery sequences. Different shades represent measurements at different magnetic fields. Green curves show fits to eq 3, while pink dashed curves indicate the contributions from low- and high-energy phonons. (c) Root-mean-square fitting errors, computed on a logarithmic scale, as a function of the transition energy separating low- and high-energy phonon regions. (d–g) Thermally weighted spectral densities of spin–lattice relaxation, visualizing the parts of the phonon spectrum that participate in the relaxation process.

To test the robustness of our method, we evaluated the SPC coefficients using different normalization cutoff energies and phonon representations. Table 1 summarizes the main results, with full details in Section 7 of the SI. Lowering the normalization cutoff energy from 600 to 380 cm^–1^ increases the normalized spectral intensities, reducing the extracted SPC coefficients by 15% for CuOEP and about 30% for CuPc. The ratio between $\lambda_{SPC}^{high}$ and $\lambda_{SPC}^{low}$ remains mostly unchanged, indicating that the relative strengths are preserved. Using the phonon DOS, rather than the measured VISION spectra, affects the extracted parameters more strongly. Because neutron weighting can distort the spectral weight distribution of the measured VISION spectrum relative to the true phonon DOS, we use the calculated relation between the VISION spectrum and the phonon DOS to estimate how these effects influence the extracted SPC parameters. In particular, $\lambda_{SPC}^{low}$ roughly doubles, whereas $\lambda_{SPC}^{high}$ changes by less than 30%, reflecting the redistribution of spectral weight toward higher energies in the DOS. In all cases, $\lambda_{SPC}^{high}$ is nearly 3 orders of magnitude larger than $\lambda_{SPC}^{low}$ .

**Table 1:** Robustness of the Extracted SPC Coefficients (in μs^–1^) against Variations in Normalization Cutoff Energy (in cm^–1^) and Phonon Representation^a^

			**CuPc**		**CuOEP**	
Analysis condition	Phonon spectrum	*E*_cutoff_	$\lambda_{SPC}^{low}$	$\lambda_{SPC}^{high}$	$\lambda_{SPC}^{low}$	$\lambda_{SPC}^{high}$
Reference	VISION	600	0.068	127	0.033	20
Normalization	VISION	380	0.045	95	0.028	17
Phonon representation	DOS	600	0.135	111	0.082	26

a The reference corresponds to fits of Figure 5.

This method also allows us to visualize which parts of the vibrational spectrum contribute most strongly to spin relaxation. Figure 5d–g shows the spectral contributions to 1/*T*_1_ (integrand of eq 3) at different temperatures, overlaid with their phonon spectra for reference (light-shaded curves). At low temperatures, relaxation is dominated by low-energy modes below 50 cm^–1^. The contribution from the strongly coupled optical modes above 185 cm^–1^ becomes evident already at 30 K and grows quickly at higher temperatures. By contrast, modes in the 50–185 cm^–1^ range contribute only weakly to spin relaxation across all temperatures. Their effect is limited by both their lower population relative to lower-energy modes and their weaker coupling compared to higher-energy phonons. This explains why the red-shifted spectrum of CuOEP compared to CuPc in this region (gray shaded area in Figure 3a) has little effect on its spin relaxation. An alternative fit that introduces a third energy window to isolate this spectral region gives the same qualitative result: although the fitted coefficients shift slightly, modes between 50 and 185 cm^–1^ still contribute significantly less to spin relaxation than lower- and higher-energy vibrational modes (see Figures S52 and S53).

In the low-energy/low-temperature regime, spin–lattice relaxation follows a thermodynamic behavior: all phonon modes are weakly coupled to the spin, and their contribution depends solely on their thermal population. Here, CuOEP’s weaker coupling results in slightly slower relaxation despite its more highly populated spectrum below 50 cm^–1^ (Figure 4a, 10 K). A strongly coupled regime emerges when modes above 185 cm^–1^ become thermally populated. Beyond the symmetric stretching mode predicted by ligand-field models to be the most strongly coupled,^12–14^ this spectral region includes in-plane scissoring modes, torsions, and higher-order out-of-plane ruffling and saddling motions. In this regime, the coupling in CuPc is 6.4 times stronger than in CuOEP (127 vs 20 μs^–1^).

In ligand-field theory, SPC is expected to increase as the energy separation to excited electronic states decreases,^13,14^ but this does not explain the stronger coupling in CuPc, since CuOEP has slightly lower ligand-field transition energies. Instead, we attribute the weaker SPC in CuOEP to a combination of weaker intermolecular interactions and a stiffer first coordination sphere. This stiffening, reflected in its higher-energy symmetric Cu–N stretching modes, effectively isolates the localized optical modes that couple most strongly to spins from collective lattice motions, consistent with previously proposed vibrational decoupling strategies.^23–26^ Smaller computed Grüneisen parameters for CuOEP further support this interpretation, indicating that its low-energy phonons are less sensitive to collective lattice expansion than those of CuPc. Overall, the out-of-plane β-ethyl substituents appear to act as vibrational dampers, reducing coupling between the molecular core and the surrounding lattice.

This picture is also reflected in the calculated root-mean-squared displacements (RMSDs), which capture the average thermal vibrational amplitudes of atoms (see SI Section 8). At 300 K, although CuOEP shows larger overall atomic displacements than CuPc (0.32 vs 0.24 Å), its molecular core (Cu and first-coordination N atoms) is more rigid, with smaller RMSDs (0.15 vs 0.17 Å).

Lastly, although lowering molecular symmetry can increase the number of phonon modes that couple to spins,^14^ it does not necessarily increase SPC strength. In CuOEP, the symmetric Cu–N stretching motion is spread across several mixed modes instead of being concentrated in one dominant vibration. Using the calculated eigenvectors, we quantified the symmetric-stretching character of each mode. In CuPc, the symmetric stretching is concentrated near 256 cm^–1^, whereas in CuOEP it is distributed across several modes, with the highest-ranked ones at 350, 288, and 268 cm^–1^ (full ranking in Section 8 of the SI). Except for the 350 cm^–1^ mode, these CuOEP vibrations show more out-of-plane motion and less in-plane Cu–N stretching than CuPc. Reported mode-resolved SPC coefficients (∂*g*/∂*Q*_*i*_)^2^ indicate that such out-of-plane distortions reduce SPC strength, and accordingly, CuOEP’s three dominant mixed stretching modes have coupling strengths reduced by factors of 10, 1.7, and 1.4 relative to CuPc’s symmetric stretch.^14,15^ Together with the higher stretching energies, this helps explain the slower spin–lattice relaxation in CuOEP.

## Conclusion

The microscopic mechanisms of spin–lattice relaxation in paramagnetic molecules remain debated, largely because direct experimental correlations between phonon spectra and spin dynamics are scarce. By combining INS with pulse EPR, we introduce the first fully experimental framework to quantify SPC coefficients in *S* = 1/2 systems.

For CuPc and CuOEP, spin relaxation is mediated by two distinct regions of the vibrational spectrum. At low temperatures, relaxation occurs via weakly coupled lattice modes below 50 cm^–1^, whereas at higher temperatures optical phonons above ∼185 cm^–1^ become thermally populated and dominate Raman relaxation with SPC coefficients nearly 3 orders of magnitude larger. In CuOEP, distortions that break planar symmetry soften the lattice and intermolecular interactions and redistribute vibrational motion away from the Cu–N core into peripheral and out-of-plane modes. As a result, CuOEP exhibits weaker effective spin relaxation at the Cu core across the measured temperature range compared to CuPc, despite possessing a more thermally accessible vibrational spectrum.

In CuOEP, spin–phonon decoupling emerges from a planar, rigid molecular core that preserves high-energy symmetric stretches while allowing limited out-of-plane distortions that reduce intermolecular coupling. Such a strategy must be approached with care since excessive ruffling (CuOEP → CuTPP → CuTiPP) can redshift core stretches and accelerate relaxation,^15^ while bulky peripheral substituents increase highly populated low-energy phonon density that can also enhance SPC.^35^

Beyond CuPc and CuOEP, the presented approach is broadly applicable. It offers a relatively rapid, quantitative method to connect crystal structure, lattice dynamics, and spin relaxation without complex modeling or data analysis. The framework can extend to systems with multiple unpaired electrons, for example, where low-energy vibrations preclude spin coherence above liquid-nitrogen temperatures. Neutron spectroscopy provides access to the full vibrational spectrum, and deuteration could refine its weighting toward the thermodynamic phonon DOS. While mode-specific SPC coefficients remain inaccessible, quantifying coupling across energy ranges already yields valuable mechanistic insight. This combined INS–EPR approach provides an experimental pathway to probe structure–activity relationships and to guide the design of molecular qubits with improved coherence times, particularly near room temperature where quantum sensing applications are most relevant.

## Supplementary Material



## Notes

Synthesis and preparation of samples, crystal structures, and thermal expansion determined by neutron and X-ray diffraction, pulse EPR measurements and analysis of spin–lattice relaxation times, INS data processing procedures, quantification of phonon anharmonicities, spin–phonon coupling fitting procedures and sensitivity analyses, and details of phonon calculations (PDF)
