# Supplementary material for: Experimental Quantification of Spin–Phonon Coupling in Molecular Qubits Using Inelastic Neutron Scattering
Source: J Am Chem Soc. 2026 May 8;148(19):20054–63. doi: 10.1021/jacs.6c03700 (PMC13195662; doi:10.1021/jacs.6c03700)
Supplement: Supplementary file 1 [file ja6c03700_si_001.pdf]

Supporting Information for

Experimental Quantification of Spin–Phonon Coupling in Molecular Qubits using Inelastic Neutron Scattering

Stefan H. Lohaus[1], Kay T. Xia[1], Yongqiang Cheng[2], Ryan G. Hadt[1,*]

[1]Division of Chemistry and Chemical Engineering, California Institute of Technology, Pasadena, CA 91125.
[2]Neutron Scattering Division, Oak Ridge National Laboratory, Oak Ridge, TN 37831.

*Corresponding author: rghadt@caltech.edu

Table of Contents

1 SYNTHESIS OF SAMPLE COMPOUNDS 5

1.1 PREPARATION OF EPR SAMPLES 5
1.2 CRYSTAL DISTORTION: CU VS. ZN 5

2 CRYSTAL STRUCTURE – NEUTRON AND X-RAY DIFFRACTION 7

2.1 STRUCTURES AT 300K 7
2.2 LATTICE EXPANSION OF CUPC 8
2.3 LATTICE EXPANSION OF CUOEP 13
2.4 CUPC VS. CUOEP 15

3 SPIN RELAXATION – PULSE EPR DATA ANALYSIS 17

3.1 ECHO-DETECTED FIELD SWEEPS (EDFS) 17
3.2 SPIN-LATTICE RELAXATION TIMES (T_1) 19
3.3 FITTING T_1 DATA TO VIBRATIONAL ENERGIES 27
3.3.1 Debye Model Fit 27
3.3.2 Local-Mode Fit 29

4 INELASTIC NEUTRON SCATTERING DATA PROCESSING 32

4.2 BACKGROUND SUBTRACTION 36
4.3 BOSE CORRECTION 37
4.4 MULTIPHONON CORRECTION 39
4.5 ELASTIC LINE SUBTRACTION 41
4.6 PHONON DOS 42
4.7 NORMALIZATION 46

5 THERMAL PHONON POPULATION 48

6 PHONON ANHARMONICITIES 50

7 SPIN–PHONON COUPLING FITS 55

7.1 DEPENDENCE OF PHONON SPECTRUM: $S_{VISION}(E)$ VS. $G(E)$ VS. $G_{NW}(E)$ 55
7.2 DEPENDENCE OF NORMALIZATION CUTOFF ENERGY 59
7.3 DEPENDENCE OF EXPERIMENTAL FIELD AND EPR SEQUENCES 60
7.4 SMOOTH CROSSOVER DESCRIPTION OF $\lambda_{SPC}(E)$ 62
7.5 INCLUDING A THIRD WINDOW 63

8 PHONON CALCULATIONS 66

8.1 MEAN-SQUARED DISPLACEMENTS 66
8.2 STRETCHING CHARACTER OF MODES 67

9 REFERENCES 70

Table of Figures

FIGURE S1. DIFFRACTION PATTERNS OF CUPC AND CUOEP AT 300K. 7
FIGURE S2. TEMPERATURE-DEPENDENT NEUTRON DIFFRACTION PATTERNS OF CUPC. 9
FIGURE S3. GAUSSIAN FITS TO DETERMINE THE POSITION OF THE DIFFRACTION PEAKS OF CUPC. 9
FIGURE S4. TEMPERATURE-INDUCED CHANGES IN D-SPACING OF CUPC. 10
FIGURE S5. INDEXING OF DIFFRACTION PATTERNS OF CUPC. 10
FIGURE S6. COMPUTED LATTICE PARAMETERS OF CUPC. GRAY CURVES ARE EXTRACTED FROM THE MONOCLINIC MODEL. 12
FIGURE S7. TEMPERATURE-DEPENDENT NEUTRON DIFFRACTION PATTERNS OF CUOEP. 13
FIGURE S8. GAUSSIAN FITS TO DETERMINE THE POSITION OF THE DIFFRACTION PEAKS OF CUOEP. 14
FIGURE S9. TEMPERATURE-INDUCED CHANGES IN D-SPACING OF CUOEP. 14
FIGURE S10. COMPUTED LATTICE PARAMETERS OF CUOEP COMPUTED FROM THE ISOTROPIC MODEL. 15
FIGURE S11. LATTICE EXPANSION OF CUPC (BLUE) AND CUOEP (ORANGE). 16
FIGURE S12. EDFS SPECTRUM OF CUPC AT 20 K. 18
FIGURE S13. EDFS SPECTRUM OF CUOEP AT 20 K. 18
FIGURE S14. INVERSION-RECOVERY TRACES FOR CUPC. 20
FIGURE S15. SATURATION-RECOVERY TRACES FOR CUPC. 21
FIGURE S16. FITTING PARAMETERS FOR CUPC. TOP. 22
FIGURE S17. SIMPLE EXPONENTIAL FITS FOR CUPC. 23
FIGURE S18. INVERSION-RECOVERY TRACES FOR CUOEP. 24
FIGURE S19. SATURATION-RECOVERY TRACES FOR CUOEP. 25
FIGURE S20. FITTING PARAMETERS FOR CUOEP. TOP: SPIN-LATTICE RELAXATION TIME CONSTANT T_1. BOTTOM: STRETCHING FACTOR B. 26
FIGURE S21. DEBYE FITS FOR CUPC. 28
FIGURE S22. DEBYE FITS FOR CUOEP. 28
FIGURE S23. LOCAL-MODE FITS FOR CUPC. 30
FIGURE S24. LOCAL-MODE FITS FOR CUOEP. 30
FIGURE S25. RAW INELASTIC NEUTRON SCATTERING SPECTRA OF CUPC MEASURED AT VISION. 33
FIGURE S26. RAW INELASTIC NEUTRON SCATTERING SPECTRA OF CUOEP MEASURED AT VISION. 34
FIGURE S27. BACKGROUND SUBTRACTION FOR CUPC. 36
FIGURE S28. BACKGROUND SUBTRACTION FOR CUOEP. 37
FIGURE S29. BOSE-CORRECTED SPECTRA OF CUPC. 38
FIGURE S30. BOSE-CORRECTED SPECTRA OF CUOEP. 38
FIGURE S31. MULTIPHONON CONTRIBUTION IN CUPC. 39
FIGURE S32. MULTIPHONON CONTRIBUTION IN CUOEP. 40
FIGURE S33. ELASTIC LINE REMOVAL AND LOW-ENERGY DEBYE EXTRAPOLATION FOR CUPC AND CUOEP. 41
FIGURE S34. TRANSFER FUNCTIONS BETWEEN $S_{VISION}(\Omega)$ AND THE PHONON DOS $G(\Omega)$ OR THE NEUTRON-WEIGHTED PHONON DOS $G_{NW}(\Omega)$. 43
FIGURE S35. COMPUTATIONAL PHONON DOS $G(\Omega)$ AND NEUTRON-WEIGHTED DOS $G_{NW}(\Omega)$ OF CUPC FROM MEASURED $S_{VISION}(\Omega)$. 44
FIGURE S36. COMPUTATIONAL PHONON DOS $G(\Omega)$ AND NEUTRON-WEIGHTED DOS $G_{NW}(\Omega)$ OF CUOEP FROM MEASURED $S_{VISION}(\Omega)$. 45
FIGURE S37. EFFECTS OF NORMALIZATION CUTOFF ENERGY ON THE EXPERIMENTALLY MEASURED $S_{VISION}(Q,\Omega)$, THE PHONON DOS $G(\Omega)$, AND THE NEUTRON-WEIGHTED DOS $G_{NW}(\Omega)$. 47
FIGURE S38. PHONON THERMAL POPULATION OF CUPC. 48
FIGURE S39. PHONON THERMAL POPULATION OF CUOEP. 49
FIGURE S40. FITTING OF PHONON PEAKS FOR CUPC. 51
FIGURE S41. PHONON ENERGY SHIFTS AND LINEWIDTH BROADENING OF CUPC 51
FIGURE S42. QUASIHARMONIC ENERGY SHIFTS OF CUPC. 52
FIGURE S43. FITTING OF PHONON PEAKS FOR CUOEP. 52
FIGURE S44. PHONON ENERGY SHIFTS OF CUOEP. 53
FIGURE S45. QUASIHARMONIC ENERGY SHIFTS OF CUOEP. 53
FIGURE S46. COMPARISON OF ANHARMONIC PHONON SHIFTS IN CUPC AND CUOEP. 54
FIGURE S47. DEPENDENCE OF THE SPIN-LATTICE RELAXATION FITS OF CUPC ON THE PHONON SPECTRUM USED IN THE ANALYSIS. 57
FIGURE S48. DEPENDENCE OF THE SPIN-LATTICE RELAXATION FITS OF CUOEP ON THE PHONON SPECTRUM USED IN THE ANALYSIS. 58

FIGURE S49. SPC FITS FOR CUPC AT DIFFERENT FIELD POSITIONS, COLLECTED USING INVERSION-RECOVERY AND SATURATION-RECOVERY PULSE SEQUENCES. 60
FIGURE S50. SPC FITS FOR CUOEP AT DIFFERENT FIELD POSITIONS, COLLECTED USING INVERSION-RECOVERY AND SATURATION-RECOVERY PULSE SEQUENCES. 61
FIGURE S51. SPC FITS WITH A SIGMOID DESCRIPTION FOR $\lambda_{SPC}(E)$ 62
FIGURE S52. SPC FITS USING THREE ENERGY WINDOWS. 63
FIGURE S53. SPC FITS USING THREE ENERGY WINDOWS WHILE EXCLUDING PHONON MODES BELOW 15 CM^{-1}. 65

Methods

1 Synthesis of sample compounds

Copper (II) phthalocyanine (CuPc) was purchased from Sigma Aldrich. Octaethylporphyrin (H_2OEP) was obtained from Strem Chemical. Copper (II) octaethylporphyrin (CuOEP) was synthesized according to a slight modification of a reported procedure (1). In a round-bottom flask, 500 mg H_2OEP (0.93 mmol., 1 equiv.) and 187 mg copper acetate hydrate were dissolved in 160 mL of a mixture of ethanol and dichloromethane (1:1 v/v). The flask was equipped with a reflux condenser and then heated at 80 °C for 18 hrs. The solvent was removed under rotary evaporation, and then the red solids were redissolved in a minimal amount of chloroform and eluted through an alumina plug with chloroform as the eluent. The chloroform was removed under rotary evaporation to yield 515 mg CuOEP as a red crystalline powder (92% yield). The product was characterized by powder X-ray diffraction and matched the reported data.

1.1 Preparation of EPR samples

0.1% CuOEP: 6.0 mg CuOEP was dissolved in 1 mL dichoromethane to create a 10 mM solution. Separately, 60 mg zinc octaethylporphyrin (ZnOEP) (0.1 mmol.) was dissolved in 5 mL dichoromethane. 0.1 mL of the 10 mM CuOEP solution (0.0001 mmol.) was added to the ZnOEP solution, and then the solvent was removed under reduced pressure. The resulting powder was dried under vacuum.

0.1% CuPc: 2.0 mg CuPc (0.0035 mmol.) was dissolved in 5 mL sulfuric acid. Separately, 196.5 mg zinc phthalocyanine (ZnPc) (0.35 mmol.) were dissolved in 10 mL sulfuric acid. 0.5 mL of the CuPc solution (0.00035 mmol.) was added to the ZnPc solution. The resulting solution was diluted with ice water until the solids precipitated, and the supernatant was clear. The solid powder was collected by vacuum filtration and then further dried under vacuum overnight.

1.2 Crystal distortion: Cu vs. Zn

Table S1 and **Table S2** quantify the structural changes in the molecular crystals resulting from the substitution of Cu with Zn in both phthalocyanine and octaethylporphyrin systems.

Parameter	ZnPc	CuPc	Δ (Cu − Zn)	% change
a (Å)	14.5347	14.5750	+0.0403	+0.28%
b (Å)	4.8529	4.7928	−0.0601	−1.24%
c (Å)	17.1927	17.1280	−0.0647	−0.38%
β (°)	106.201	105.687	−0.514	−0.48%
V (Å³)	1164.54	1151.91	−12.62	−1.08%

Table S1. *Crystal lattice parameters for CuPc (2) and ZnPc (3), and comparison of their structural parameters.*

Parameter	ZnOEP	CuOEP	Δ(Cu − Zn)	% change
a (Å)	4.6920	4.8050	+0.1130	+2.41%
b (Å)	13.1850	13.3140	+0.1290	+0.98%
c (Å)	13.2870	13.3920	+0.1050	+0.79%
α (°)	113.94	113.08	−0.86	−0.75%
β (°)	91.177	92.42	+1.243	+1.36%
γ (°)	92.157	93.38	+1.223	+1.33%
V (Å³)	750.16	784.80	+34.63	+4.62%

Table S2. *Crystal lattice parameters for CuOEP (4) and ZnOEP (5), and comparison of their structural parameters.*

2 Crystal structure – Neutron and X-ray diffraction

2.1 Structures at 300K

The crystal structures of CuPc and CuOEP were characterized by powder X-ray diffraction (PXRD) using a Rigaku SmartLab diffractometer with Cu Kα radiation (λ = 1.5406 Å, $K\alpha_1$) and a Kβ filter. The patterns were Rietveld refined in GSAS-II using the reported single-crystal structures as starting models (2, 4). The 300 K diffraction patterns and their fits are shown in **Figure S1**, confirming that CuPc adopts a monoclinic structure and CuOEP a triclinic one. The refined lattice parameters are summarized **Table S3**. CuPc is known to crystallize in different stacking polymorphs, and comparison with literature data shows that our sample corresponds to the most stable β phase.

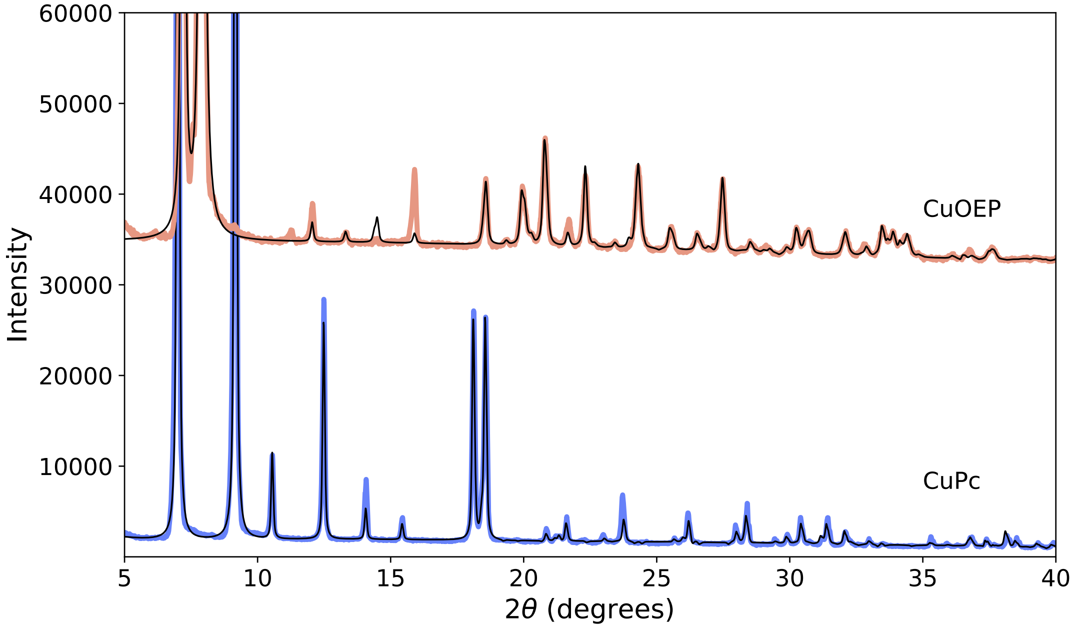

Figure S1. *Diffraction patterns of CuPc and CuOEP at 300K. Colored curves are X-ray measurements and black curves correspond to Rietveld refinements.*

sample	crystal	a (Å)	b (Å)	c (Å)	α (deg)	β (deg)	γ (deg)	Vol (Å³)
CuPc	monoclinic	14.6777	4.7845	17.3499	90	105.359	90	1174.882
CuOEP	triclinic	13.3059	13.3937	4.8212	92.369	93.355	113.046	787.343

Table S3. *Lattice parameters of CuPc and CuOEP determined from Rietveld refinements (GSAS-II) of PXRD patterns collected at room temperature.*

2.2 Lattice expansion of CuPc

Neutron diffraction measurements were performed in-situ on the VISION spectrometer. In time-of-flight mode, a dedicated elastic-scattering detector bank records the $E \approx 0$ signal over a broad range of momentum transfer, while the analyzer spectrometer simultaneously measures the inelastic phonon spectrum. This setup allows us to follow lattice changes as a function of temperature and relate them to phonon shifts through mode-resolved Grüneisen parameters (see Section 6). Since the samples are protiated, hydrogen contributes with a large incoherent scattering background, which limits the number of observable Bragg peaks. Nevertheless, the remaining peaks are sufficient to quantify the lattice expansions of both CuPc and CuOEP.

The temperature-dependent neutron diffraction patterns of CuPc are shown in **Figure S2**. To enable a direct comparison with the PXRD data, all diffraction patterns are plotted on the *d*-spacing scale using Bragg's law:

$$d = \frac{\lambda}{2\sin\theta}$$

We fitted the most prominent diffraction peaks with Gaussian functions (**Figure S3**). The resulting temperature-dependent changes in *d*-spacing, shown in **Figure S4**, are plotted both as absolute values and as fractional shifts along different crystallographic directions.

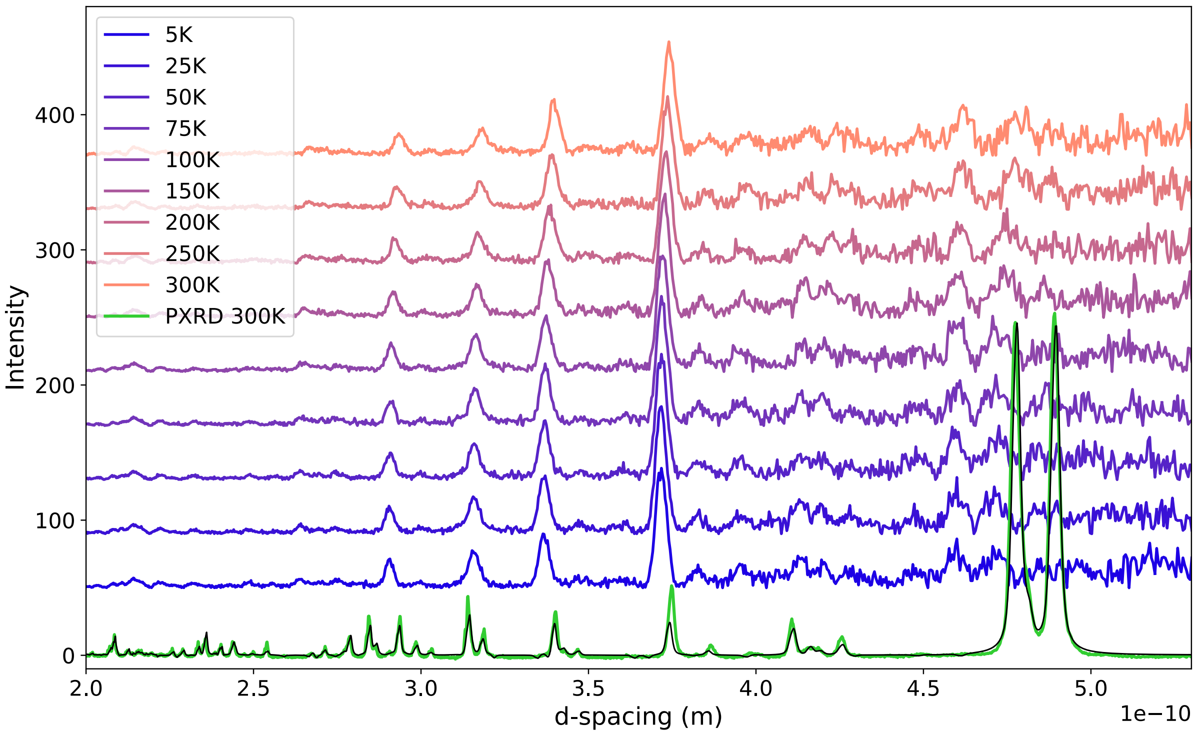

Figure S2. Temperature-dependent neutron diffraction patterns of CuPc.

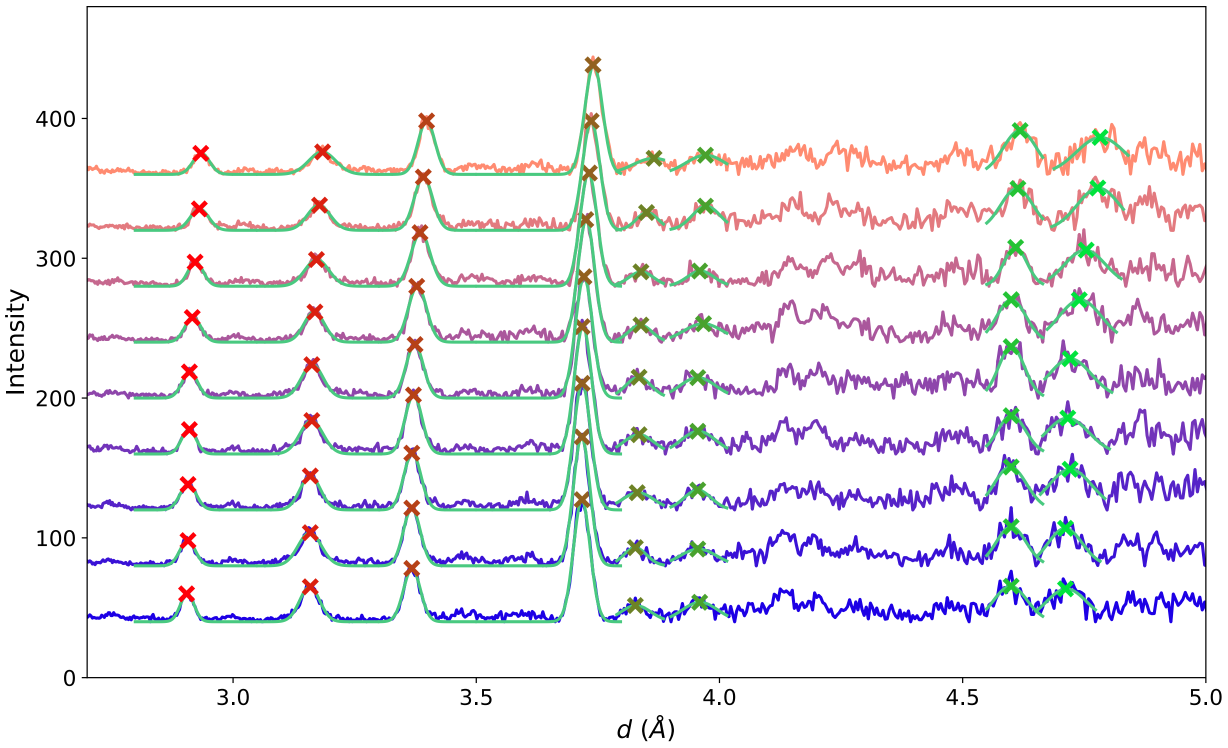

Figure S3. Gaussian fits to determine the position of the diffraction peaks of CuPc (marked with 'x').

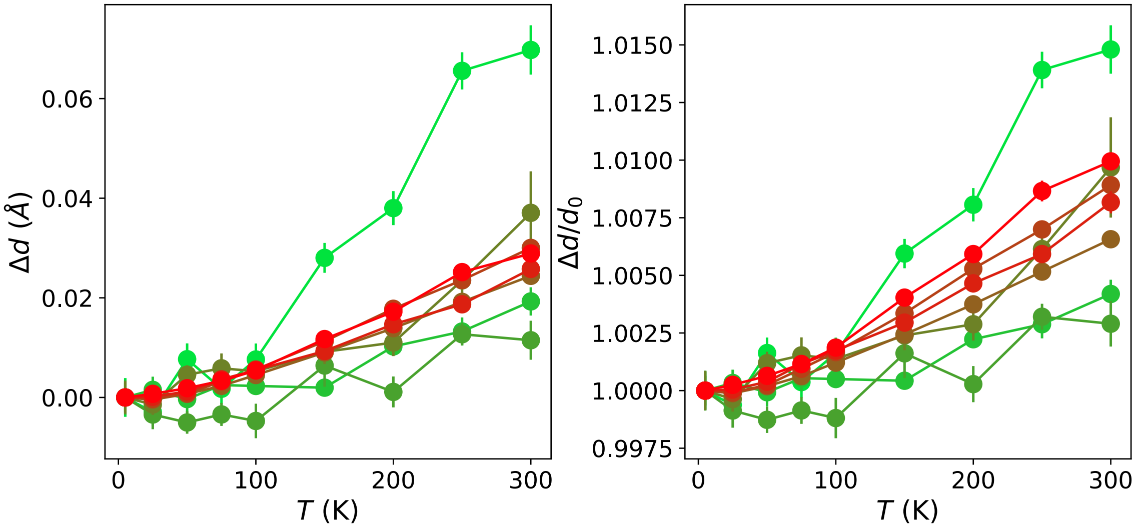

Figure S4. *Temperature-induced changes in d-spacing of CuPc. Left: absolute changes; right: fractional changes. Colors correspond to different crystallographic directions according to Figure S3. Error bars represent the standard deviation of the fitted peak parameters.*

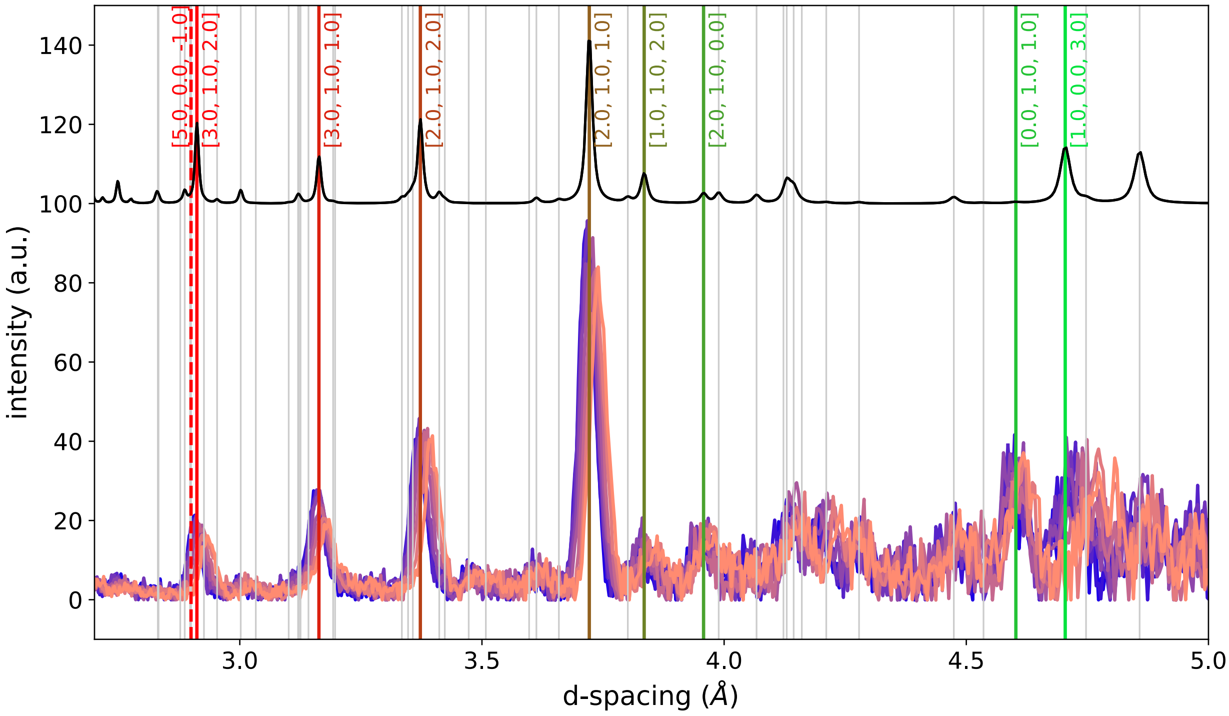

Figure S5. *Indexing of diffraction patterns of CuPc. Black spectrum is the literature reference, and gray vertical lines are the reported peak positions. Colored vertical lines are the assigned indices for our measurements.*

To extract lattice parameters, the diffraction peaks were indexed. Peak positions were compared to single-crystal reference patterns (**Figure S5**), and the closest matching Miller indices [h k l] were assigned. All peaks could be indexed unambiguously except for the highest order reflection at the smallest *d*-spacing, for which two possible index assignments are considered in the analysis. As shown later in **Figure S6**, both index assignments yield the same temperature trend for the lattice parameters and for the cell volume.

CuPc has a monoclinic structure, so the lattice parameters are related to the measured *d*-spacings and the Miller indices by:

$$\frac{1}{d_{hkl}^2} = \frac{h^2}{a^2 \sin^2 \beta} + \frac{k^2}{b^2} + \frac{l^2}{c^2 \sin^2 \beta} - \frac{2hl \cos \beta}{ac \sin^2 \beta}$$

which can be rewritten as

$$\frac{1}{d_{hkl}^2} = Ah^2 + Bk^2 + Cl^2 - 2Dhl$$

with

$$a = \frac{1}{\sqrt{A} \sin \beta}; \quad b = \frac{1}{\sqrt{B}}; \quad a = \frac{1}{\sqrt{C} \sin \beta}; \quad \beta = \cos^{-1}\left(\frac{D}{\sqrt{AC}}\right)$$

and the unit cell volume:

$$V = abc \sin \beta$$

The lattice parameters *a, b, c,* β, and *V* were obtained at each temperature by fitting the indexed *d*-spacings with a linear least-squares procedure applied to the equation above. Because only six diffraction peaks are available, the angle β was assumed to be temperature independent and fixed to the PXRD value of 105.359°. Weighting was included in the fit, since reflections at larger *d* have lower intensity and less reliable peak positions, while high-*Q* (low *d*) reflections are more sensitive to small lattice distortions. A weight of $1/d^2$ ensures that each peak contributes according to its fractional rather than absolute shift, otherwise reflections with large *d* would dominate the fit because $\Delta d = s \cdot d_0$.

The resulting temperature-dependent lattice parameters are shown in **Figure S6** as gray curves, with each curve corresponding to one of the two possible indexing assignments. Horizontal lines mark the room-temperature values from PXRD. The parameters are physically reasonable, particularly for *a* and *c*, but overestimate the parameter *b.* The resulting parameters also exhibit noise and non-monotonic behavior, which reflects the limited number of peaks used for the fitting.

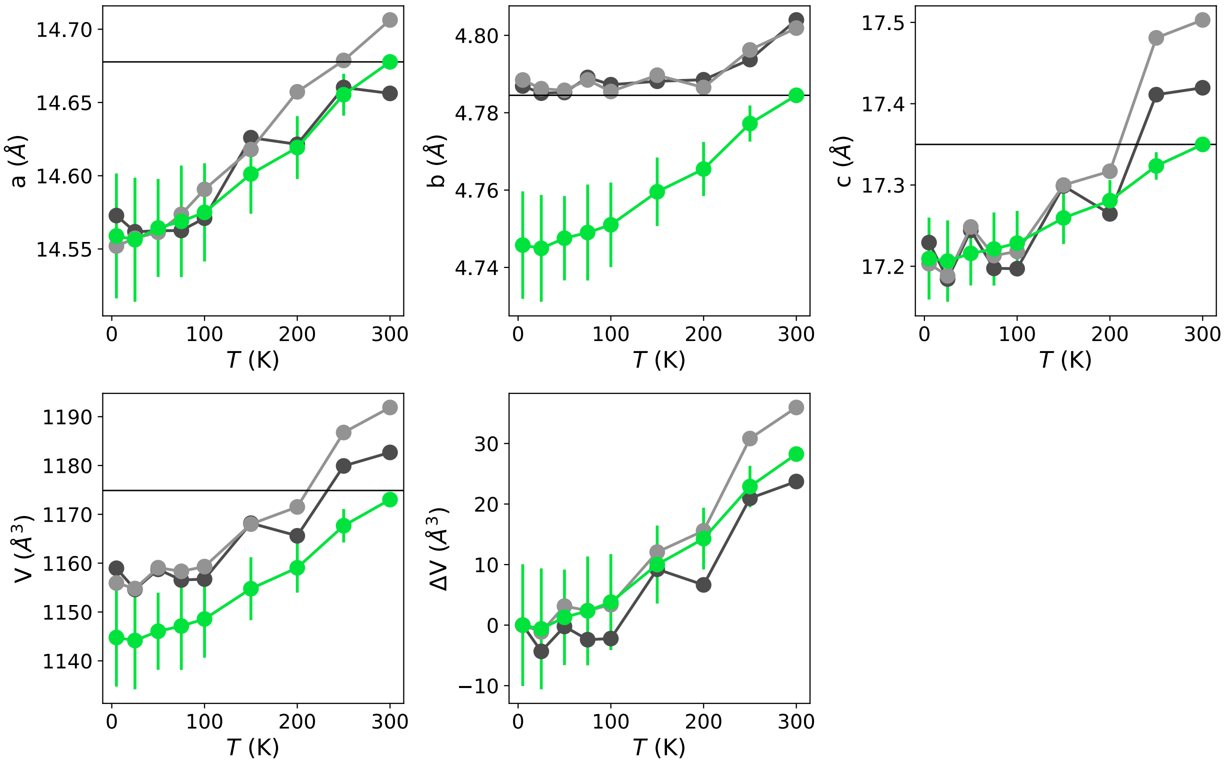

Figure S6. *Computed lattice parameters of CuPc. Gray curves are extracted from the monoclinic model (each for a different index assignment), and the green curves correspond to the isotropic model. Horizontal lines are values measured by PXRD at room temperature.*

An alternative approach is to assume that the lattice expands isotropically. In this model the thermal expansion is described by a single strain parameter $s(T)$ that scales all lattice constants uniformly, while the unit cell angles remain fixed at their room temperature PXRD values:

$$a(T) = a_0\big(1 + s(T)\big), \quad b(T) = b_0\big(1 + s(T)\big), \quad c(T) = c_0\big(1 + s(T)\big), \quad V(T) = V_0\big(1 + s(T)\big)^3$$

Using the room-temperature PXRD values as the reference, $s(T)$ is obtained by a weighted least squares fit that relates the measured *d*-spacings to the reference values through

$$d_{iso}(T) = d_{ref}\big(1 + s(T)\big)$$

As in the monoclinic fit, each reflection is weighted by $1/d^2$. The resulting lattice parameters are shown in **Figure S6** as green curves. They vary smoothly with temperature and match the full monoclinic fit closely, except for the parameter *b*. For the volume expansion Δ*V*/*V*, the quantity of interest to compute Grüneisen parameters, the isotropic model agrees well with the full monoclinic result. For this reason, the main manuscript reports only the isotropic model.

The uncertainty of the isotropic model shown in **Figure S6** reflects how much the measured anisotropic *d*-spacings deviate from those predicted by an isotropic expansion. The strain along a specific direction in the crystal is

$$s_i(T) = \frac{d_i(T)}{d_i(T_{ref})} - 1$$

Error bars are defined as the weighted RMS deviation of the measured (anisotropic) strains from the isotropic fit,

$$\sigma_{iso}(T) = \sqrt{\frac{\sum_i w_i (s_i(T) - s_{iso}(T))^2}{\sum_i w_i}}$$

with $w_i = 1/d_i(T)^2$. Since the 300 K values are used as the reference, the uncertainty is zero at 300 K and increases at lower temperatures as the isotropic approximation deviates from the measured values.

2.3 Lattice expansion of CuOEP

The measured neutron diffraction patterns of CuOEP are shown in **Figure S7**. We fitted six diffraction peaks with Gaussian functions, as shown in **Figure S8**. At low temperatures, all peaks can be resolved, but above 150 K the two highest *d* reflections begin to merge and their positions become unreliable. The corresponding temperature-dependent changes of the peaks in *d*-spacing are shown in **Figure S9**.

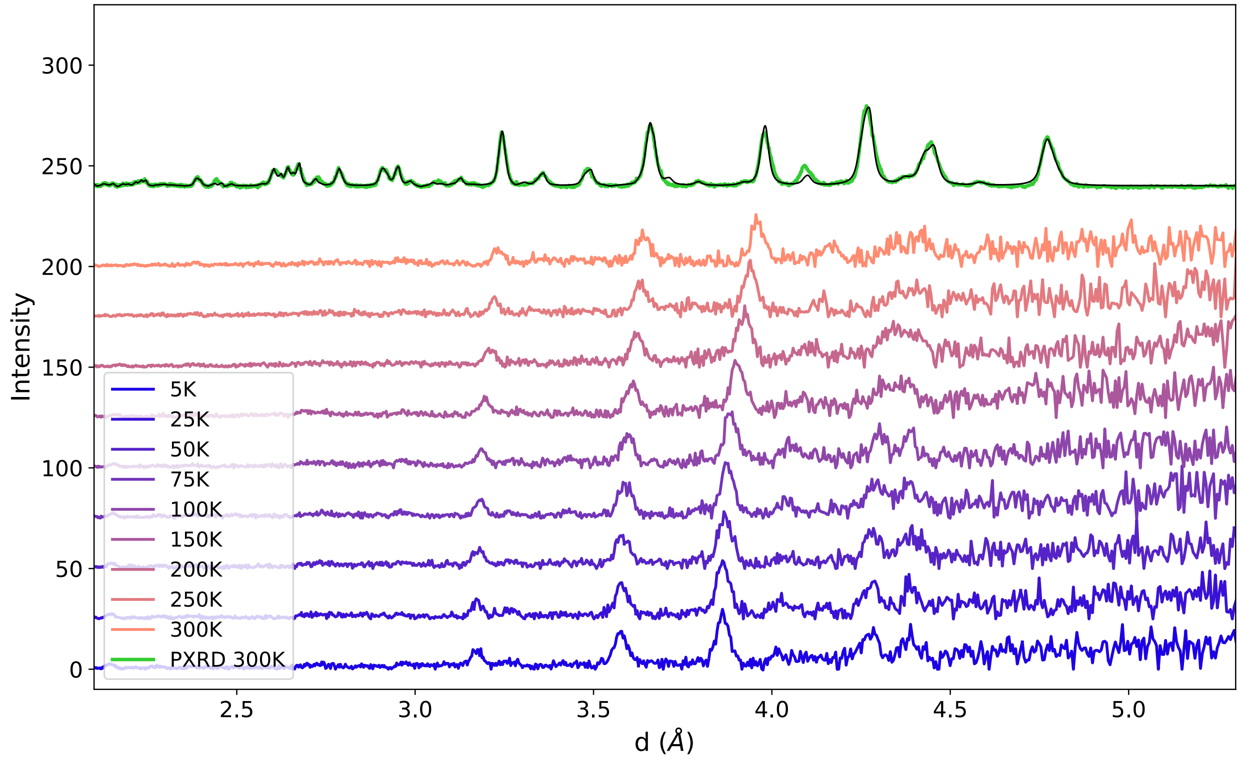

Figure S7. *Temperature-dependent neutron diffraction patterns of CuOEP.*

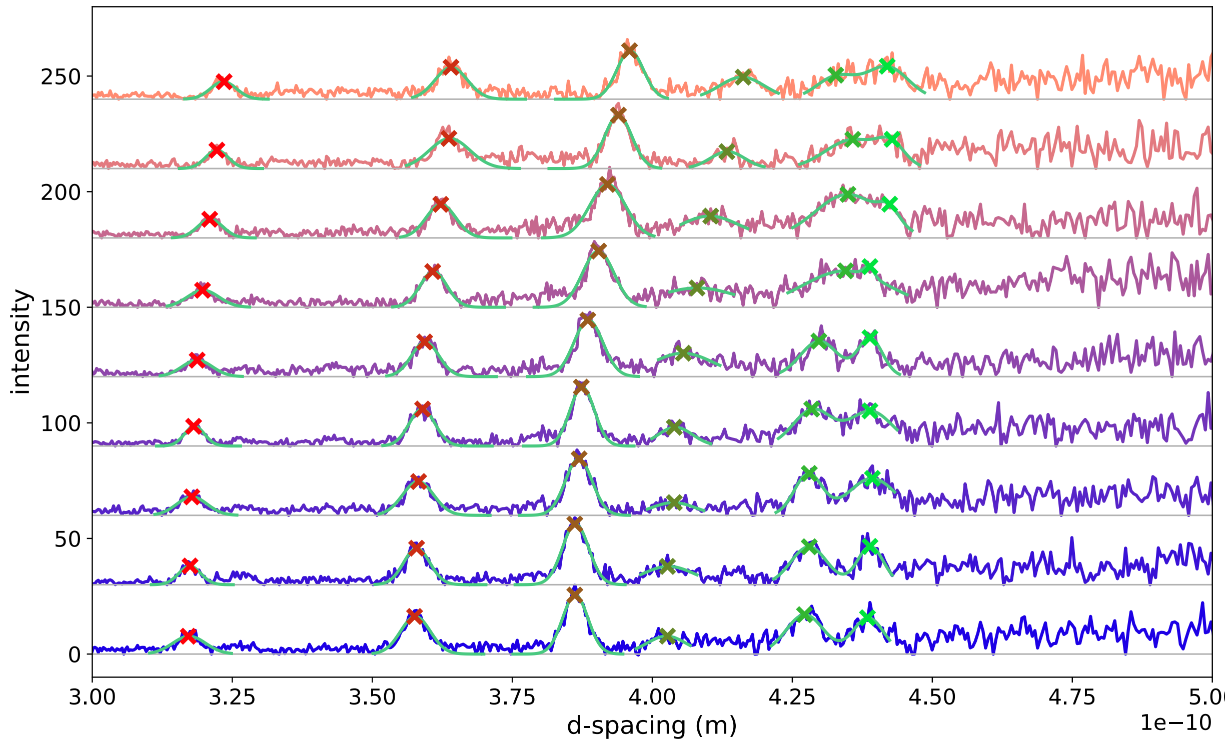

Figure S8. *Gaussian fits to determine the position of the diffraction peaks of CuOEP (marked with 'x').*

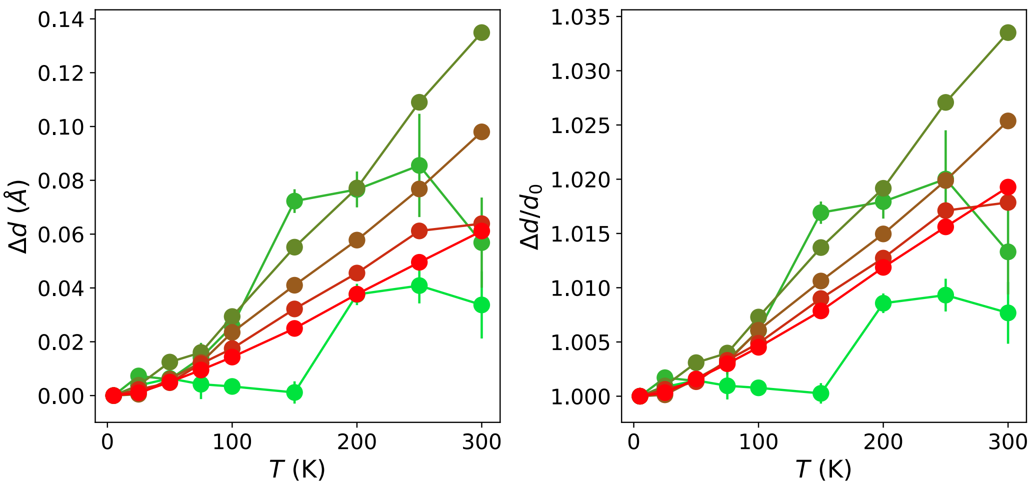

Figure S9. *Temperature-induced changes in d-spacing of CuOEP. Left: absolute changes; right: fractional changes. Colors correspond to different crystallographic directions according to Figure S8. Error bars represent the standard deviation of the fitted peak parameters.*

CuOEP crystallizes in a triclinic structure, which requires six independent lattice parameters to describe the unit cell. Since only six peaks are available and two of them cannot be accurately fitted at higher temperatures, a full triclinic refinement is not reliable (even with fixed lattice angles). We therefore apply the same isotropic expansion model used for CuPc. For the fit, we only use the four lowest-d peaks, which remain well resolved across all temperatures. The resulting temperature-dependent lattice parameters are plotted in **Figure S10**.

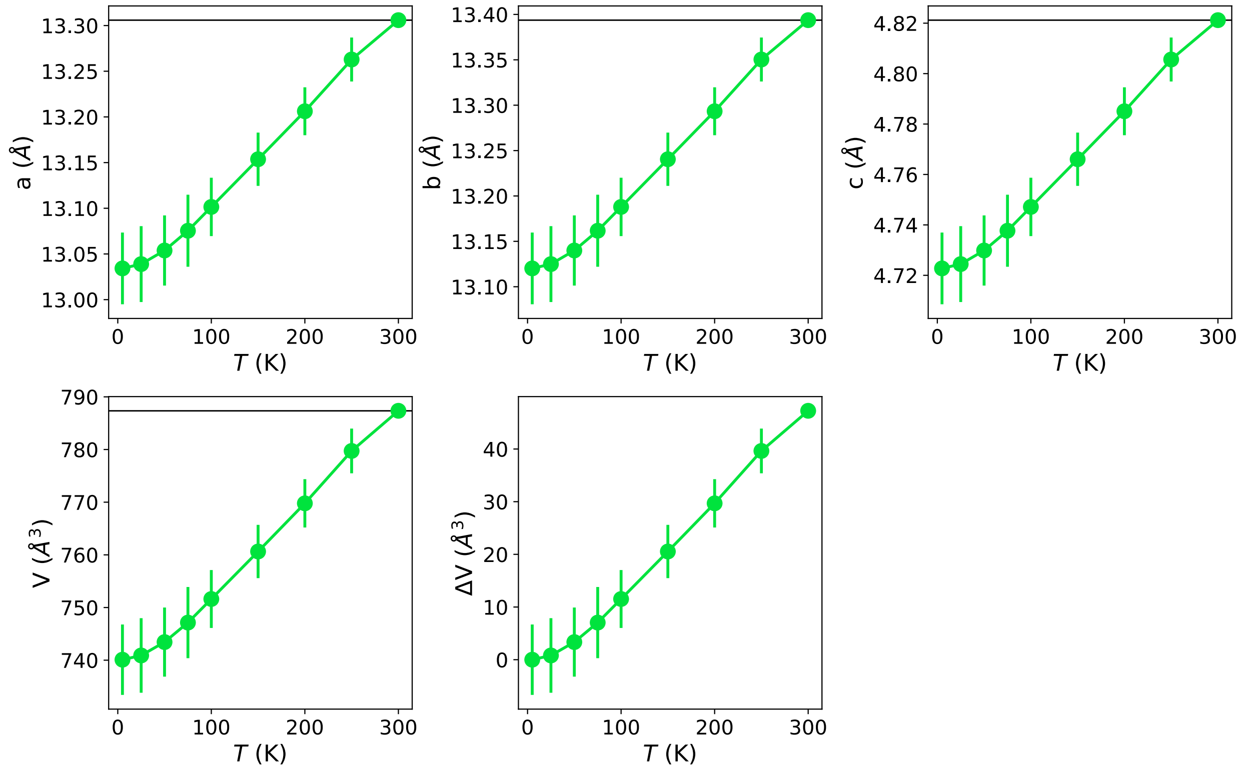

Figure S10. *Computed lattice parameters of CuOEP computed from the isotropic model. Horizontal lines are values measured by PXRD at room temperature.*

2.4 CuPc vs. CuOEP

Figure S11 compares the absolute changes in *d*-spacing for CuPc and CuOEP, as well as their absolute and fractional volume expansions. Fractional volume changes, normalized to the room-temperature unit cell volume, allow for a direct comparison between the two lattices and are therefore presented in the main manuscript. CuOEP shows a significantly larger volume expansion than CuPc, which we attribute to weaker intermolecular interactions arising from its crystal packing (see main text).

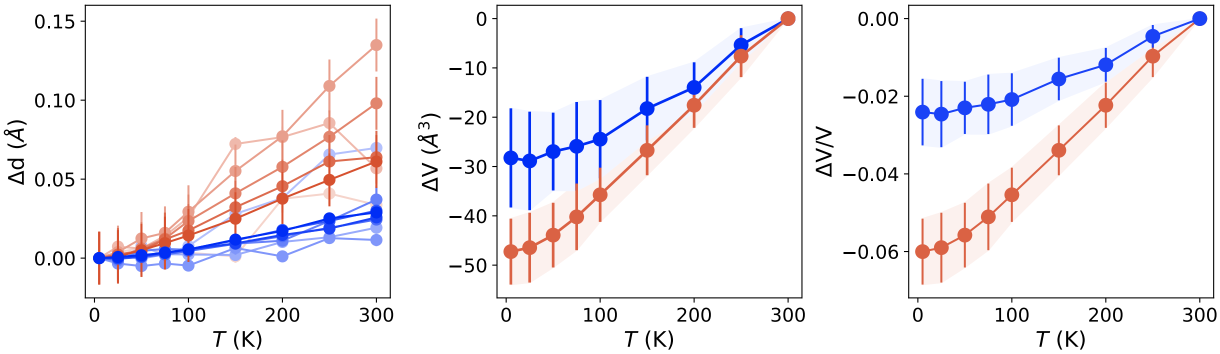

Figure S11. *Lattice expansion of CuPc (blue) and CuOEP (orange). Left: absolute changes of d-spacings normalized to 5K values. Color shades correspond to different crystallographic directions. Center: absolute volume changes obtained from the isotropic expansion model, normalized by room temperature values. Right: Fractional volume changes. Error bars show the RMSE of individual peak strains relative to the isotropic fit.*

3 Spin relaxation – Pulse EPR data analysis

Pulsed EPR measurements at X-band (~9.7 GHz) were performed on a Bruker ELEXSYS E580 spectrometer equipped with an MD-4 resonator. Samples were loaded into 4 mm Norell quartz EPR tubes and cooled using an Oxford Instruments CF-935 liquid-helium flow cryostat regulated by a Mercury temperature controller, enabling measurements down to 3.5 K. To minimize dipole–dipole interactions and measure the effect of phonons, CuPc and CuOEP were diluted 1:1000 into isostructural diamagnetic hosts (ZnPc and ZnOEP).

Echo-detected field sweeps (EDFS) were acquired using the standard two-pulse Hahn-echo sequence (π/2 – τ – π – τ – echo), while varying the interpulse delay τ. Longitudinal relaxation times (T_1) were determined by inversion-recovery experiments employing the π – t – π/2 – τ – π – τ – echo pulse sequence, where t is a variable delay and τ was fixed and optimized to maximize the echo amplitude. Typical π/2 and π pulse lengths were 8 ns and 16 ns, respectively. For each field position and temperature, the echo amplitude was optimized by adjusting the video gain. Four-step phase cycling was applied in inversion-recovery experiments to suppress unwanted echoes and microwave ring-down.

At low temperatures, where T_1 is long, spectral diffusion can contribute to the apparent relaxation times, so T_1 was also measured using initial saturation-recovery pulses, which suppress diffusion effects. Saturation-recovery measurements employed a train of eight π pulses spaced by 1 μs (picket-fence scheme), followed by the inversion-recovery sequence. Measurements at all fields and temperatures were collected sequentially in a single session for each sample. The cavity resonance frequency was matched for both samples.

3.1 Echo-detected field sweeps (EDFS)

EDFS spectra reveal resonant transitions between spin-up and spin-down states of unpaired electrons. Each spectrum reflects the anisotropic g-tensor and the hyperfine coupling to nearby nuclear spins. In pulsed EPR experiments, the microwave frequency is held constant while the external magnetic field is swept. Resonance occurs when the Zeeman energy splitting matches the photon energy, producing detectable changes in echo intensity. The EDFS spectra of CuPc and CuOEP measured at 20 K (**Figure S12** and **Figure S13**) exhibit the characteristic axial powder pattern of Cu(II) centers, with resolved Cu hyperfine splitting.

Because the electron Zeeman interaction is anisotropic, the resonance condition depends on the orientation of molecular crystallites relative to the external field. For CuPc and CuOEP, specific field positions correspond to molecular orientations parallel and perpendicular to the field (6, 7). For the T_1 measurements, these two orientations were selected as indicated by the vertical lines in the figures.

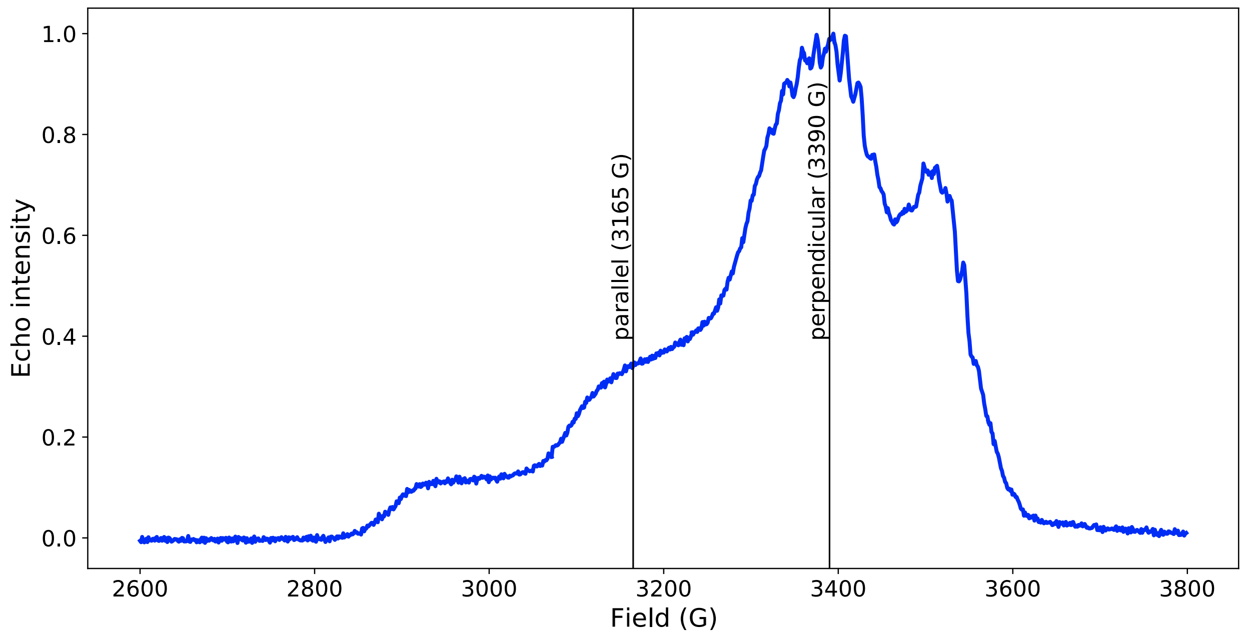

Figure S12*. EDFS spectrum of CuPc at 20 K.*

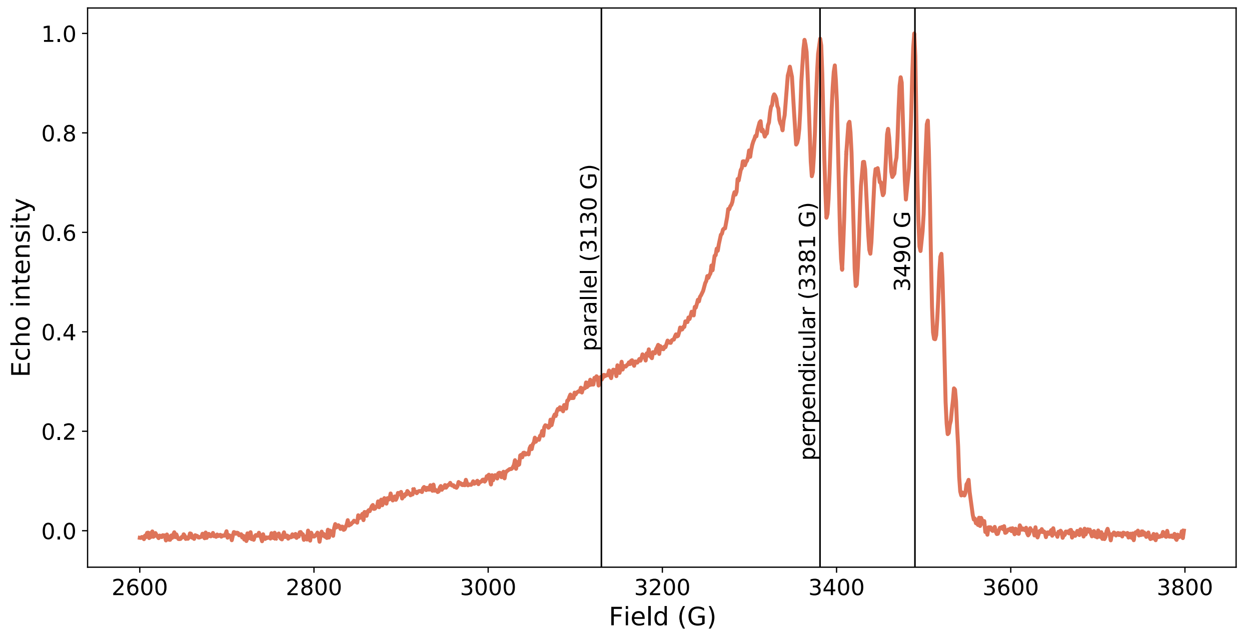

Figure S13. *EDFS spectrum of CuOEP at 20 K.*

3.2 Spin-lattice relaxation times (T_1)

Inversion-recovery and saturation-recovery measurements were fitted to stretched-exponential functions (using nonlinear regression):

$$I(t) = Ae^{-\left(\frac{t+d}{T_1}\right)^{\beta}} + I_0$$

where *t+d* is the total delay after the initial π pulse (*d* being a fixed delay ensuring that the total evolution time exceeds the pulse length). Error bars represent 95% confidence intervals for the fitted parameters T_1 and β. Inversion-recovery data and fits for CuPc are shown in **Figure S14**, while saturation-recovery results are presented in **Figure S15**. The extracted T_1 and β values from both methods are summarized in **Figure S16.**

Saturation-recovery measurements isolate the intrinsic spin–lattice relaxation process from spectral diffusion by first saturating the spin ensemble with a train of π pulses (picket-fence scheme) before monitoring the recovery. For CuPc, spectral diffusion becomes significant below 15 K, where relaxation times are long. Consequently, saturation-recovery yields systematically longer T_1 values than inversion-recovery under these conditions. In the main text, we report saturation recovery measurements below 30K and inversion recovery at higher temperatures.

The stretching factor β increases with temperature from approximately 0.5–0.6 at low temperatures to 0.8–0.9 at higher temperatures. Above 100 K, where relaxation is fast, the echo intensity becomes weak, resulting in increased scatter and less reliable β fits. To validate the stretched-exponential model, the relaxation traces were also fitted with single-exponential functions (β = 1). As shown in Figure S17, the single-exponential fits reproduce the data poorly at low temperatures. At high temperatures, where spins relax rapidly and the magnetization is already partially recovered at the start of the measurement window (echo intensity > –1), the single-exponential model fails to capture the correct extrapolation. Overall, the simple-exponential fits yield longer T_1 values, although the temperature dependence $T_1(T)$ remains consistent, supporting the validity of the stretched-exponential analysis.

The same fitting analysis for CuOEP is presented in **Figure S18**–
Figure S20. The overall trends mirror those observed for CuPc. The fits are slightly worse (larger residuals) for CuOEP at the two lowest temperatures. At higher temperatures, all measurements converge to similar T_1 values. Above approximately 30 K, no significant differences are observed between T_1 values obtained at different field positions or through inversion- and saturation-recovery.

CuOEP remains coherent at room temperature, allowing measurement of T_1 (and T_m) up to 300 K. However, the echo signal from our 1:1000 solid dilution in ZnOEP was too weak to determine T_1 at room temperature. Instead, we use measurements from a 1:100 dilution sample reported in (6).

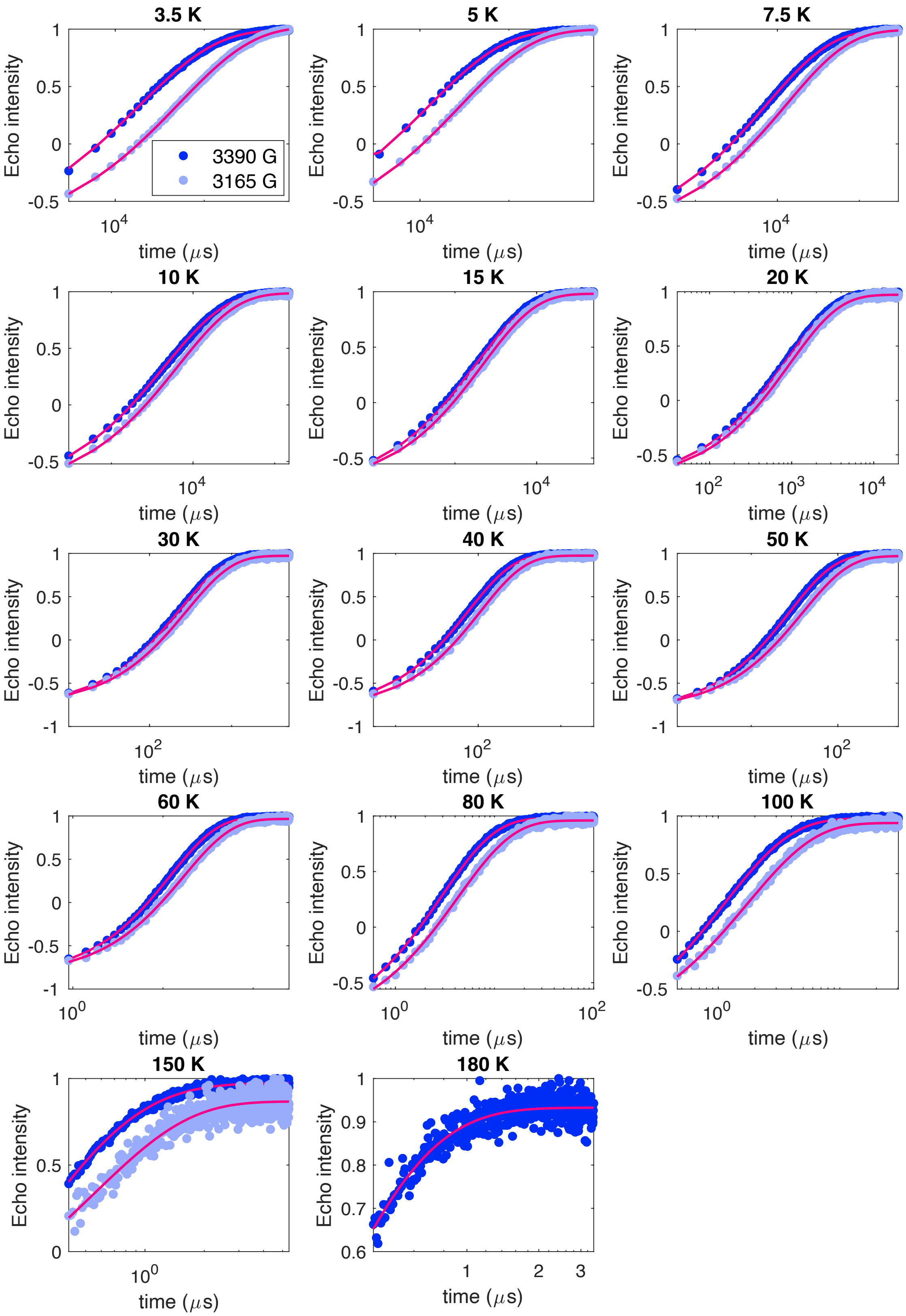

Figure S14. *Inversion-recovery traces for CuPc, shown with their stretched-exponential fits and on logarithmic time axes.*

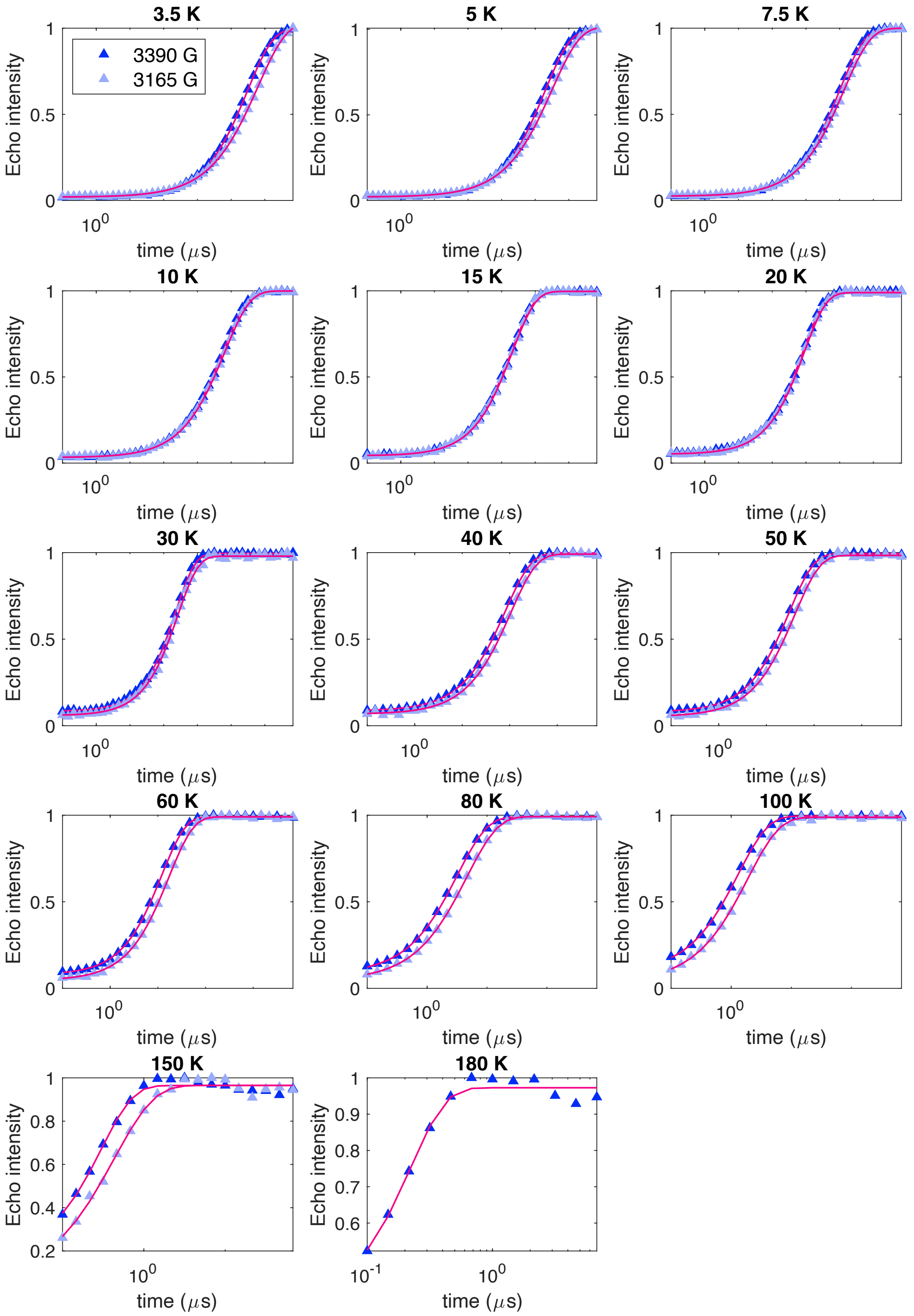

Figure S15. *Saturation-recovery traces for CuPc, shown with their stretched-exponential fits and on logarithmic time axes.*

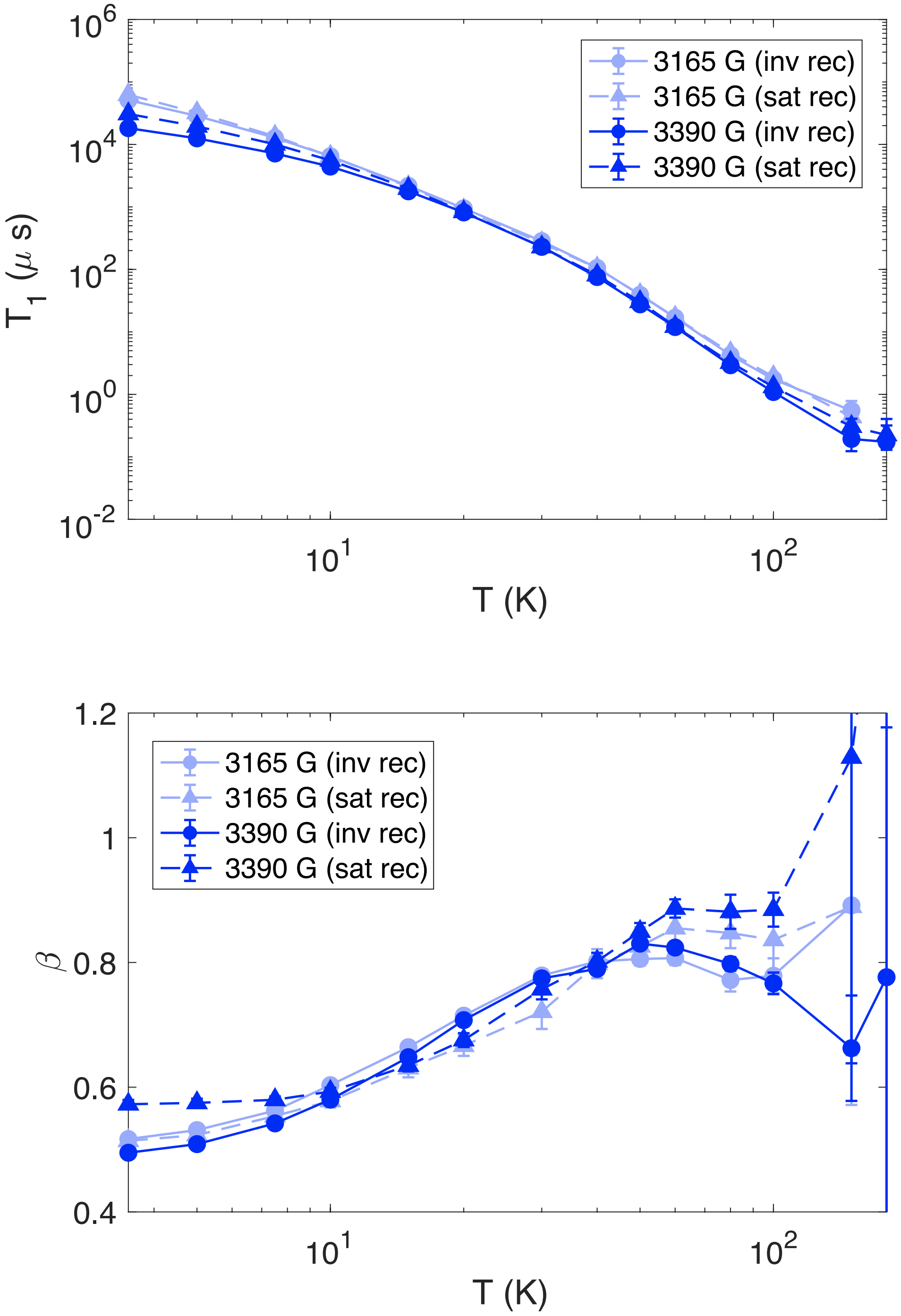

Figure S16. *Fitting parameters for CuPc. Top: Spin-lattice relaxation time constant T_1. Bottom: Stretching factor β.*

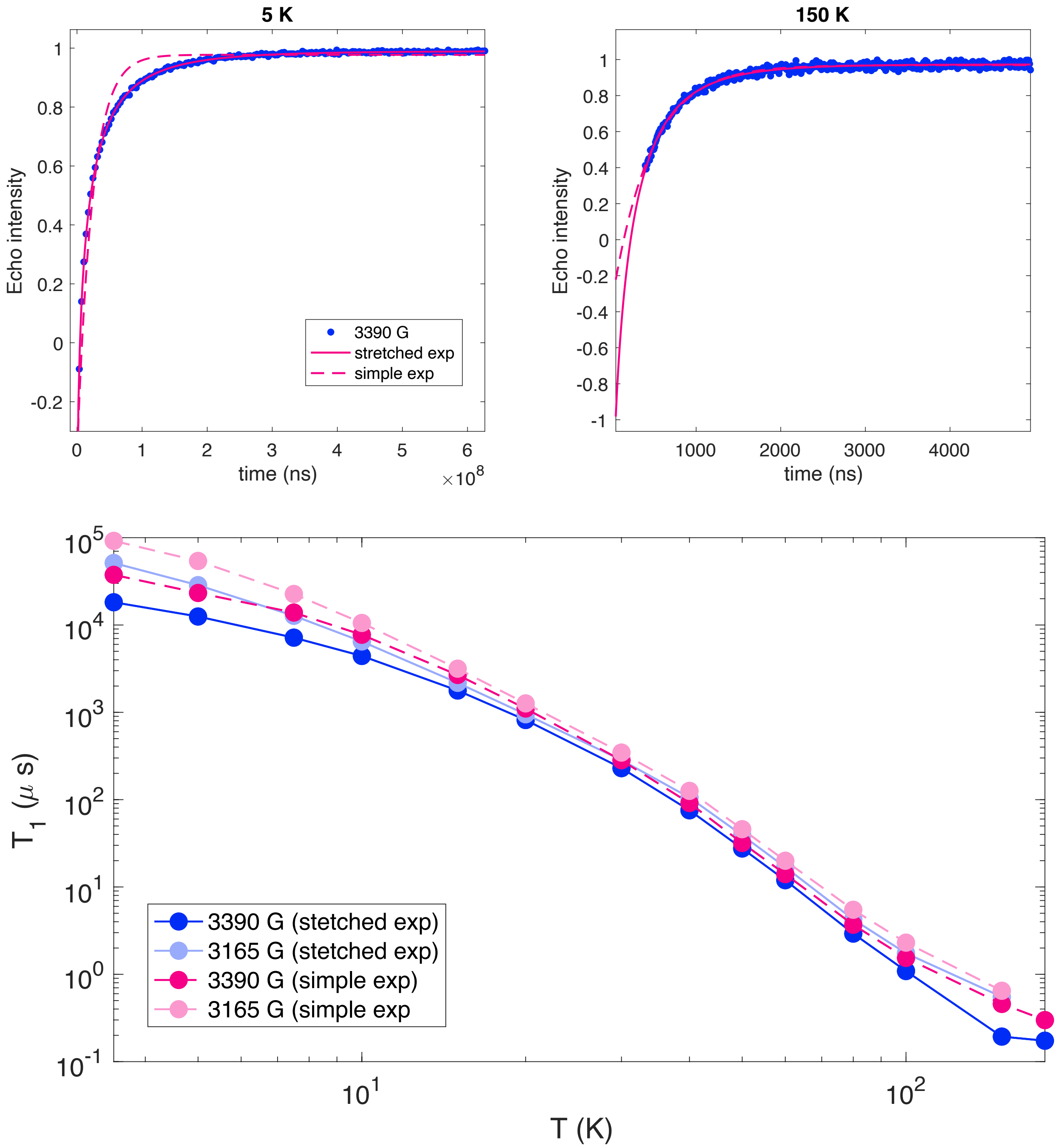

Figure S17. *Simple exponential fits for CuPc. Top: relaxation traces and fits at 5 and 150K measured by inversion recovery. Bottom: Fitted T1 values for inversion recovery measurements (from simple vs. stretched exponential fits)*

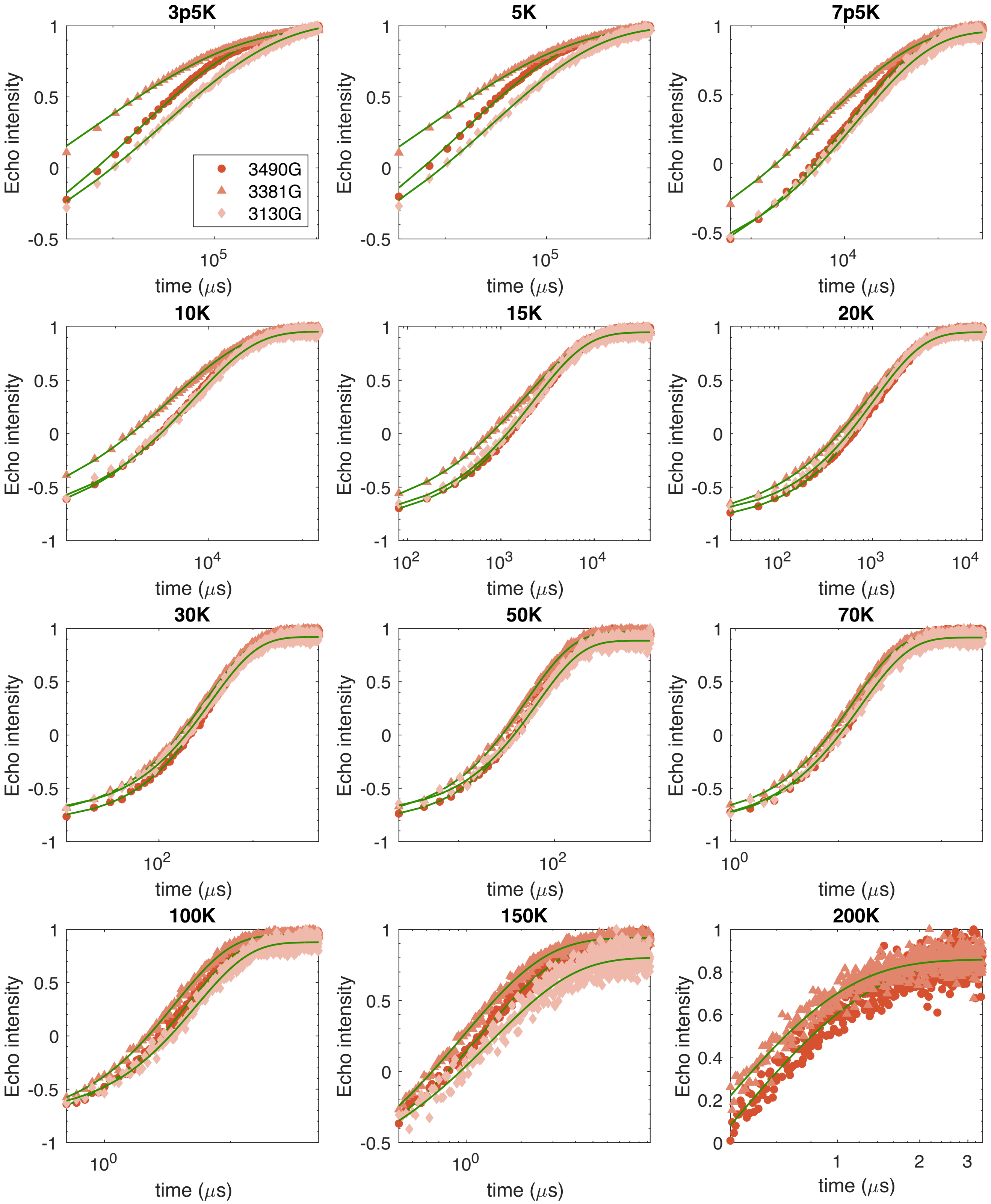

Figure S18. *Inversion-recovery traces for CuOEP, shown with their stretched-exponential fits and on logarithmic time axes.*

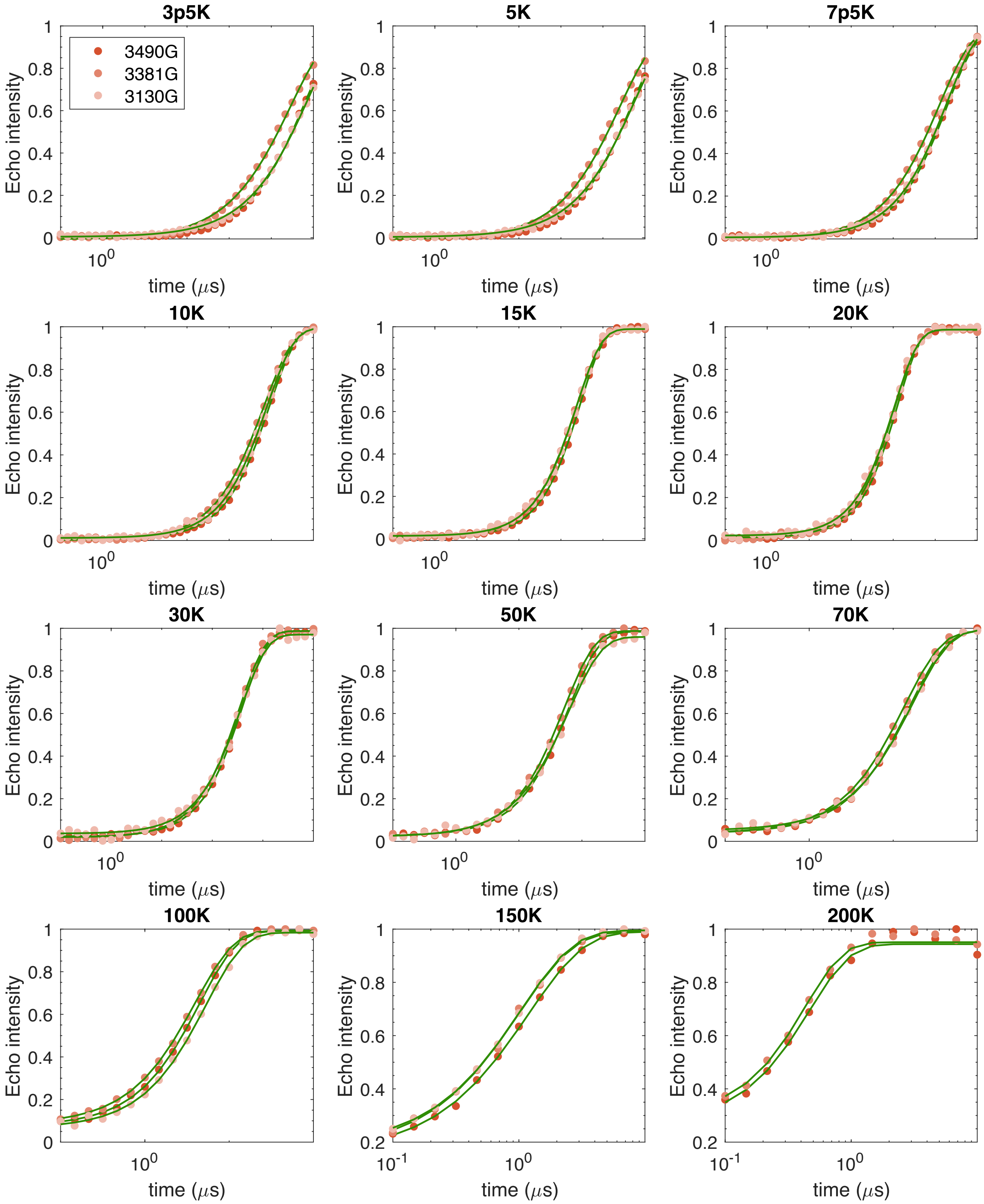

Figure S19. *Saturation-recovery traces for CuOEP, shown with their stretched-exponential fits and on logarithmic time axes.*

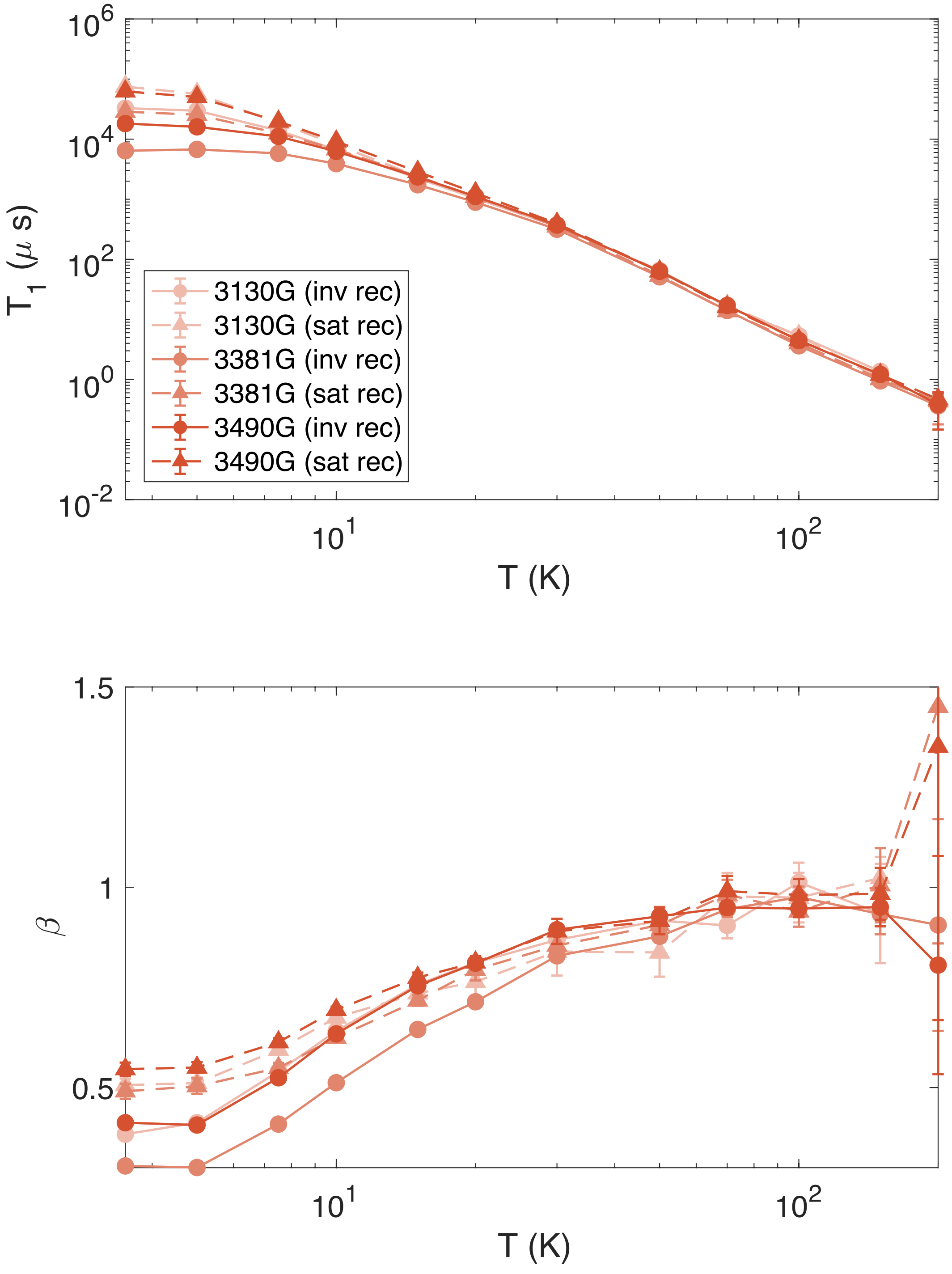

Figure S20. *Fitting parameters for CuOEP. Top: Spin-lattice relaxation time constant T_1. Bottom: Stretching factor β.*

3.3 Fitting T_1 data to vibrational energies

To extract the characteristic energies of vibrational modes contributing to spin relaxation, several models have been employed in the literature. The *direct* relaxation process, relevant primarily at very low temperatures, is commonly modeled with a linear temperature dependence. At higher temperatures, *two-phonon* relaxation mechanisms are typically analyzed using either a *Debye model* or a *local-mode model*.

3.3.1 Debye Model Fit

The Debye model assumes a linear phonon dispersion and a quadratic phonon density of states. Although this approximation is valid only for low-energy acoustic modes (below ~15 cm^{-1}), it is frequently applied in the high-temperature regime, where $1/T_1 \propto T^2$. To account for deviations from this strict quadratic dependence, T_1 traces are often fitted with a power-law expression in which the exponent is treated as a free parameter. Fits yielding exponents larger than 2 are commonly interpreted as a sign of enhanced spin–phonon coupling, although such behavior lacks a defined mechanism.

Following common practice, we fitted T_1 (T) data using a model that combines (i) a linear direct process, (ii) a power-law Debye contribution, and (iii) a local-mode term at higher temperatures:

$$\frac{1}{T_1} = A_{dir}T + A_{Debye}T^n + A_{loc}\frac{e^{E_{loc}/k_BT}}{(e^{E_{loc}/k_BT} - 1)^2}$$

The exponential term reflects the probability of a two-phonon scattering process. Assuming that both involved modes have the same energy E_{loc}, the probability of having one phonon already excited is given by the Bose–Einstein distribution $n(E_{loc}, T)$. The probability of creating another phonon of the same energy is $n(E_{loc}, T) + 1$. The combined probability of the two-phonon process is therefore $n(E_{loc}, T) \cdot (n(E_{loc}, T) + 1)$, which corresponds to the exponential term in the expression.

The resulting fits for CuPc and CuOEP are shown in **Figure S21** and **Figure S22**, and the corresponding fitted parameters and fitting errors are summarized in **Table S4**. Errors for a few fitted parameters of CuOEP are large, suggesting that local mode fits are more appropriate.

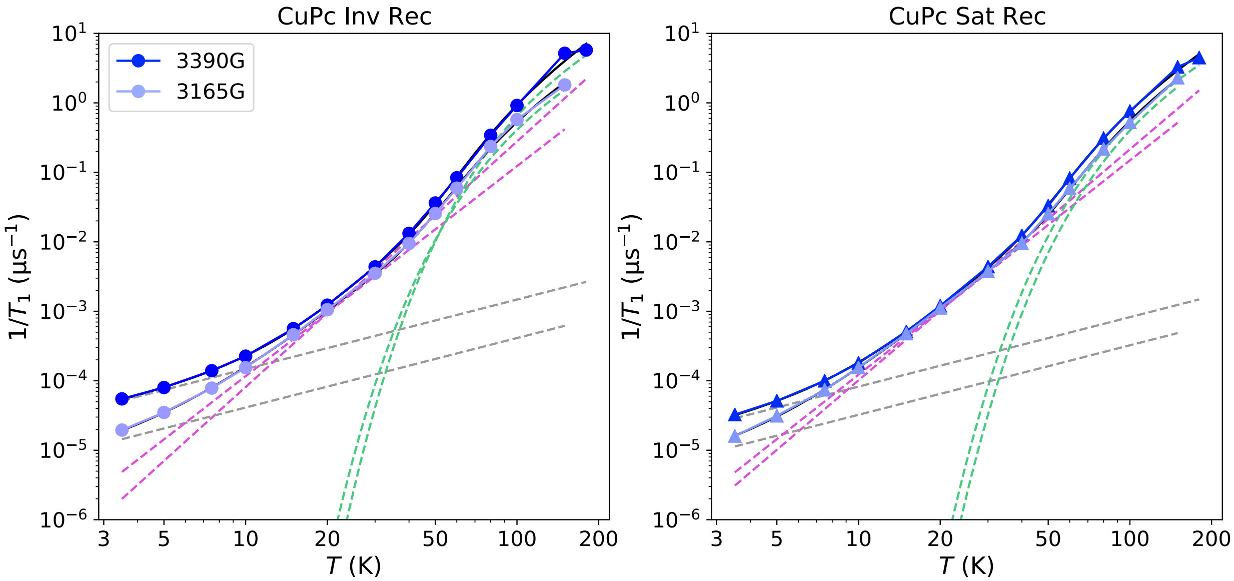

Figure S21. *Debye fits for CuPc. Left: Inversion-recovery measurements. Right: Saturation-recovery measurements. Gray lines correspond to the direct relaxation (i), pink lines to the Debye model (ii), and green curves to the local mode fits (iii).*

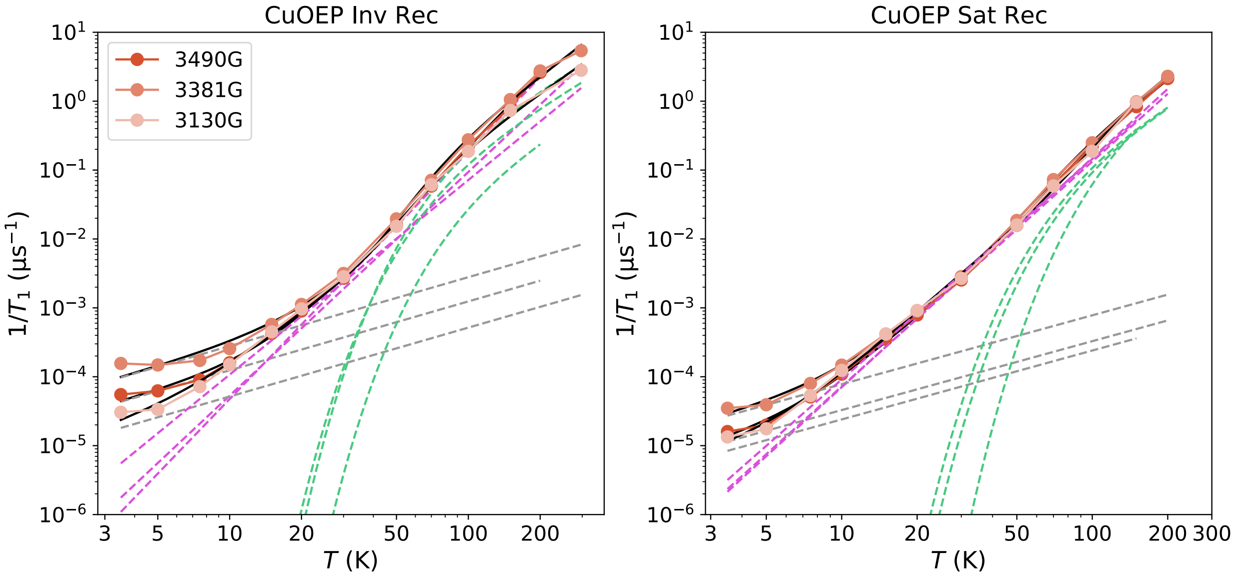

Figure S22. *Debye fits for CuOEP. Left: Inversion-recovery measurements. Right: Saturation-recovery measurements. Gray lines correspond to the direct relaxation (i), pink lines to the Debye model (ii), and green curves to the local mode fits (iii).*

Sample	Measurement	Field (G)	Exponent n	E_{loc} (cm^{-1})
CuPc	Inversion Rec	3390	3.53 (± 0.20)	287.0 (± 16.5)
		3165	3.02 (± 0.07)	249.6 (± 7.3)
	Saturation Rec	3390	3.32 (± 0.09)	260.1 (± 7.6)
		3165	3.08 (± 0.04)	279.4 (± 5.4)
CuOEP	Inversion Rec	3490	3.59 (± 0.18)	262.5 (± 173.1)
		3381	3.25 (± 0.39)	220.4 (± 40.6)
		3130	2.82 (± 0.25)	198.4 (± 29.8)
	Saturation Rec	3490	3.26 (± 0.13)	257.0 (± 31.7)
		3381	3.33 (± 0.15)	233.6 (± 29.2)
		3130	3.20 (± 0.15)	375.7 (± 131.8)

Table S4. *Fitted parameters obtained from the Debye model. Standard fitting errors are shown in parentheses.*

3.3.2 Local-Mode Fit

An alternative to the Debye model is to describe relaxation solely in terms of *local vibrational modes*. Their corresponding exponential expressions provide direct access to the phonon energies involved in the relaxation process, whereas the Debye model does not specify which vibrational modes contribute. Additionally, the local-mode model yields the same high-temperature limit as the Debye model, $1/T_1 \propto T^2$, corresponding to a power-law dependence.

$$\frac{1}{T_1} = A_{dir}T + A_{1,loc}\frac{e^{E_{1,loc}/k_BT}}{\left(e^{E_{1,loc}/k_BT} - 1\right)^2} + A_{2,loc}\frac{e^{E_{2,loc}/k_BT}}{\left(e^{E_{2,loc}/k_BT} - 1\right)^2}$$

Using a sum of local-mode terms thus offers a more physically meaningful description of the data, as each component can be directly associated with a specific vibrational energy. The fits and the resulting parameters are shown in **Figure S23** and **Figure S24**, with parameters presented in **Table S5**.

Sample	Measurement	Field (G)	$E_{1,loc}$ (cm^{-1})	$E_{2,loc}$ (cm^{-1})
CuPc	Inversion Rec	3390	51.2 (± 6.7)	271.9 (± 14.2)
		3165	29.1 (± 2.6)	227.4 (± 9.3)
	Saturation Rec	3390	39.1 (± 3.7)	243.8 (± 9.3)
		3165	29.1 (± 2.8)	242.2 (± 12.7)
CuOEP	Inversion Rec	3490	48.9 (± 7.9)	271.7 (± 24.6)
		3381	65.1 (± 18.3)	260.9 (± 34.1)
		3130	32.1 (± 3.4)	207.5 (± 12.5)
	Saturation Rec	3490	31.5 (± 2.9)	232.1 (± 13.1)
		3381	36.5 (± 4.4)	229.5 (± 13.0)
		3130	28.6 (± 2.9)	232.7 (± 19.8)

Table S5. *Fitted parameters obtained from the local-mode model. Standard fitting errors are shown in parentheses.*

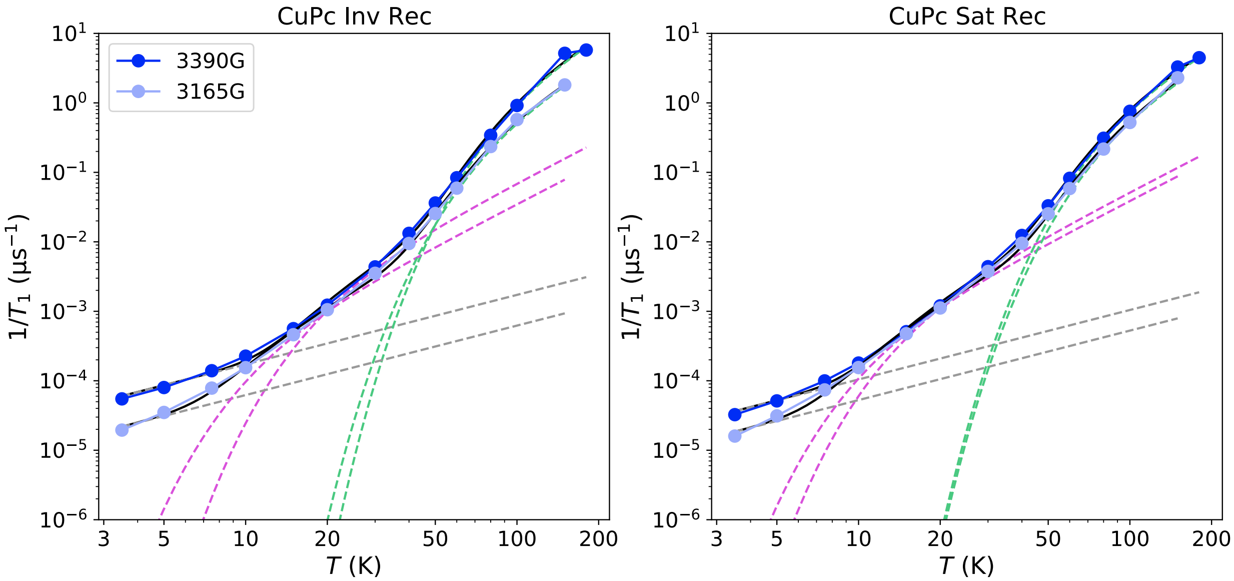

Figure S23. *Local-mode fits for CuPc. Left: Inversion-recovery measurements. Right: Saturation-recovery measurements. Gray lines correspond to the direct relaxation, pink curves to the first local mode, and green curves to the second local mode fit.*

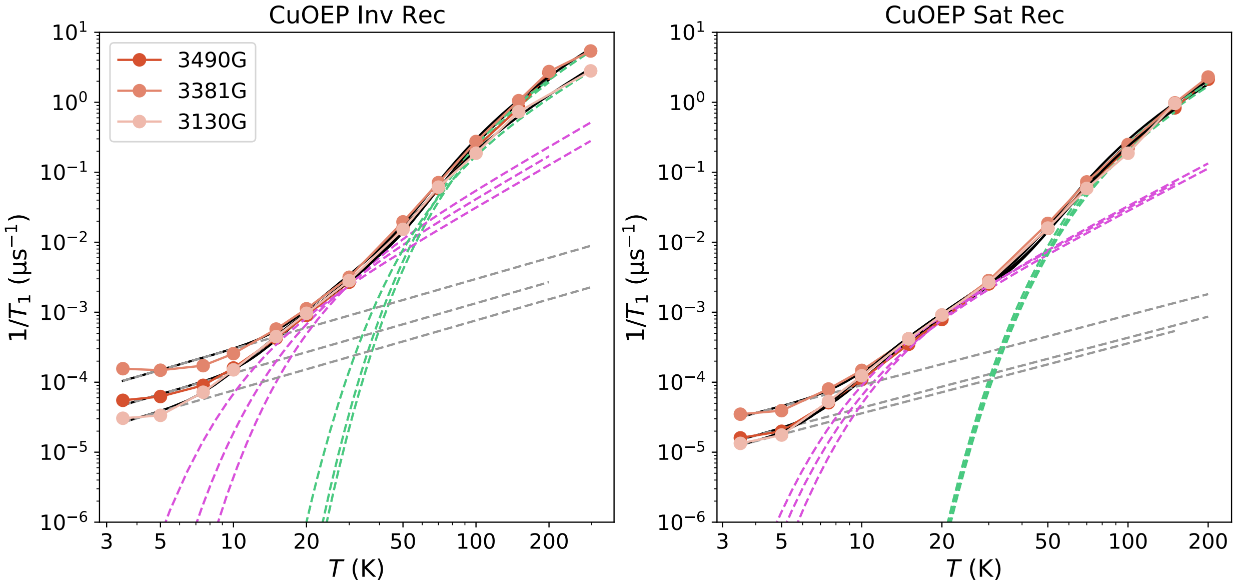

Figure S24. *Local-mode fits for CuOEP. Left: Inversion-recovery measurements. Right: Saturation-recovery measurements. Gray lines correspond to the direct relaxation, pink curves to the first local mode, and green curves to the second local mode fit.*

4 Inelastic neutron scattering data processing

Neutrons interact directly with atomic nuclei without optical selection rules and transfer sufficient momentum to probe vibrations across the entire Brillouin zone. Unlike X-rays or optical probes, neutrons primarily interact with atomic nuclei rather than electronic states, thereby minimizing radiation damage in molecular and inorganic materials such as CuPc and CuOEP. Measurements were carried out on the VISION spectrometer, on beamline BL-16B of the Spallation Neutron Source**,** Oak Ridge National Laboratory (8, 9). VISION operates in an inverted-geometry configuration in which pulses of white neutrons excite vibrations in the sample, and the scattered neutrons are reflected by crystal analyzers before being detected. By collecting scattering over a wide range of angles, VISION yields a momentum-averaged phonon density of states. Although momentum resolution is lost, this design enables rapid, high-sensitivity measurements of molecular powders, capturing the entire phonon range from −16 to 8000 cm^{-1} with relatively small amounts of sample.

For our experiments, approximately 500 mg of CuPc or CuOEP powder was sealed in thin-walled vanadium cans (6 mm diameter) under helium for thermal equilibration. Data were collected between 5–300 K with a white incident neutron beam, for 20 min (CuPc) and 30 min (CuOEP) per temperature, corresponding to proton charges at the target of 0.8 and 1.2 C, respectively. Data were reduced using the MANTID software package (10). The raw INS spectra collected at VISION are shown in Figure S25 and Figure S26. In addition to the inelastic phonon signal, the spectra contain strong elastic peaks and background contributions from the cryostat, sample can, and multiple-phonon scattering. The data were normalized by the incident neutron flux.

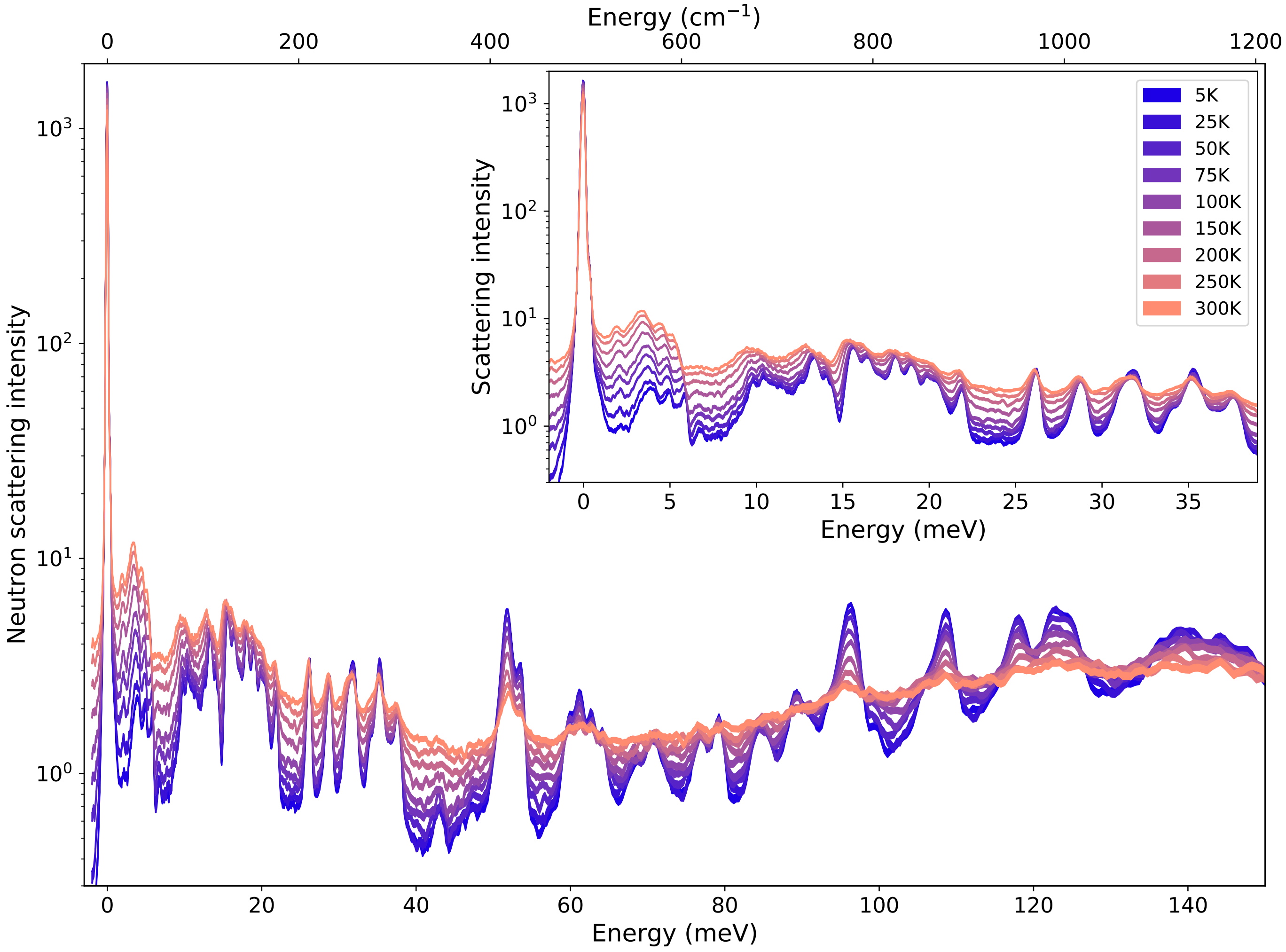

Figure S25. *Raw inelastic neutron scattering spectra of CuPc measured at VISION.*

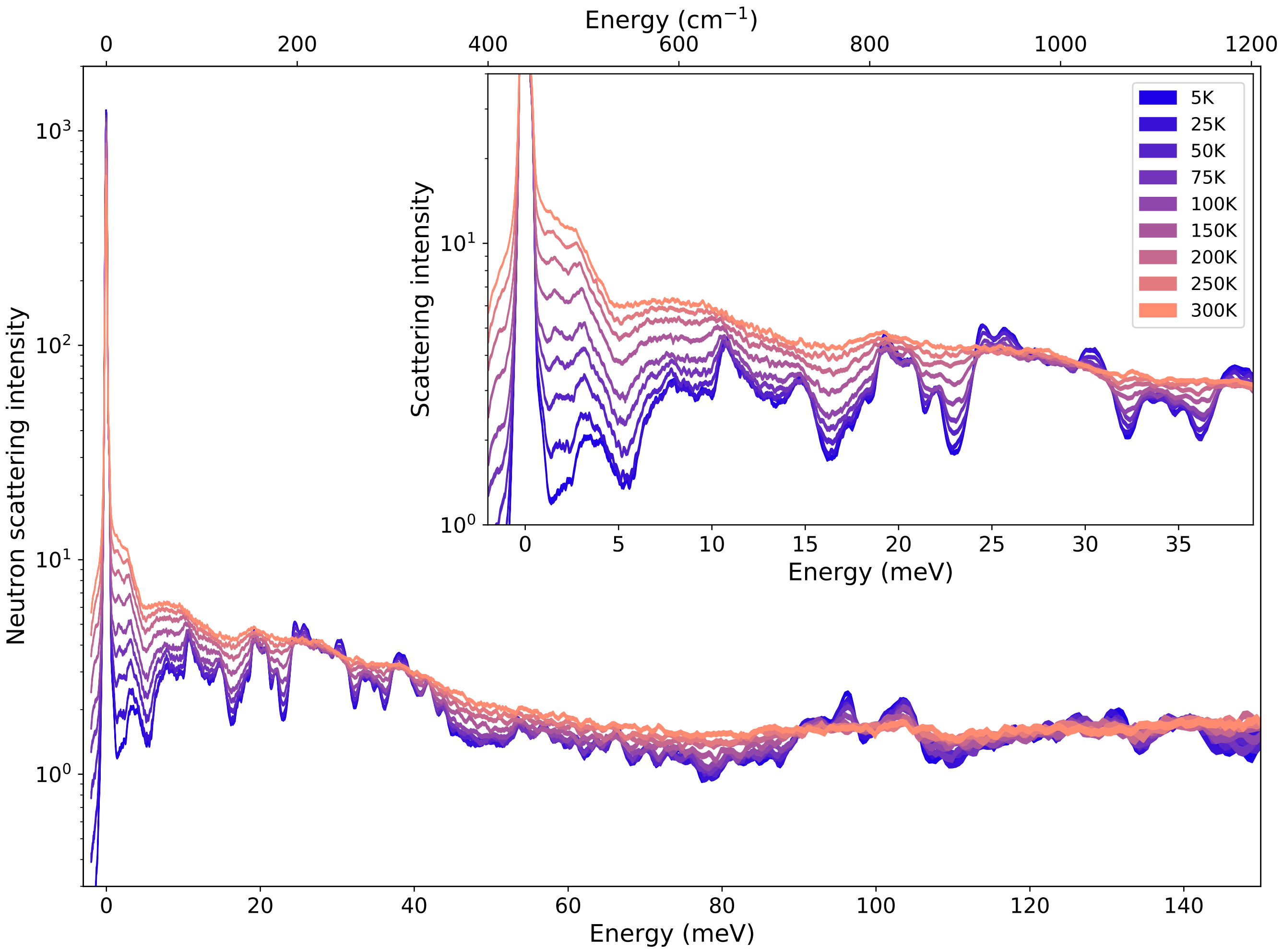

Figure S26. *Raw inelastic neutron scattering spectra of CuOEP measured at VISION.*

4.2 Background subtraction

Neutrons interact with all materials along their path, so it is necessary to subtract the scattering contributions from the aluminum-walled closed-cycle refrigerator and the vanadium cans used to hold the powder samples (which contribute minimally). Accordingly, we measured the signal of an empty can and subtracted it from the signal obtained from the samples. **Figure S27** and **Figure S28** show the original spectra (colored curves), the spectra after background subtraction (black curves), and the empty-can measurement at 5 K (green curve). The empty-can signal exhibits three main features: a strong elastic-line shoulder below 1 meV, and two distinct peaks at 4 and 6.7 meV (dotted lines), which disappear in the background-subtracted spectra.

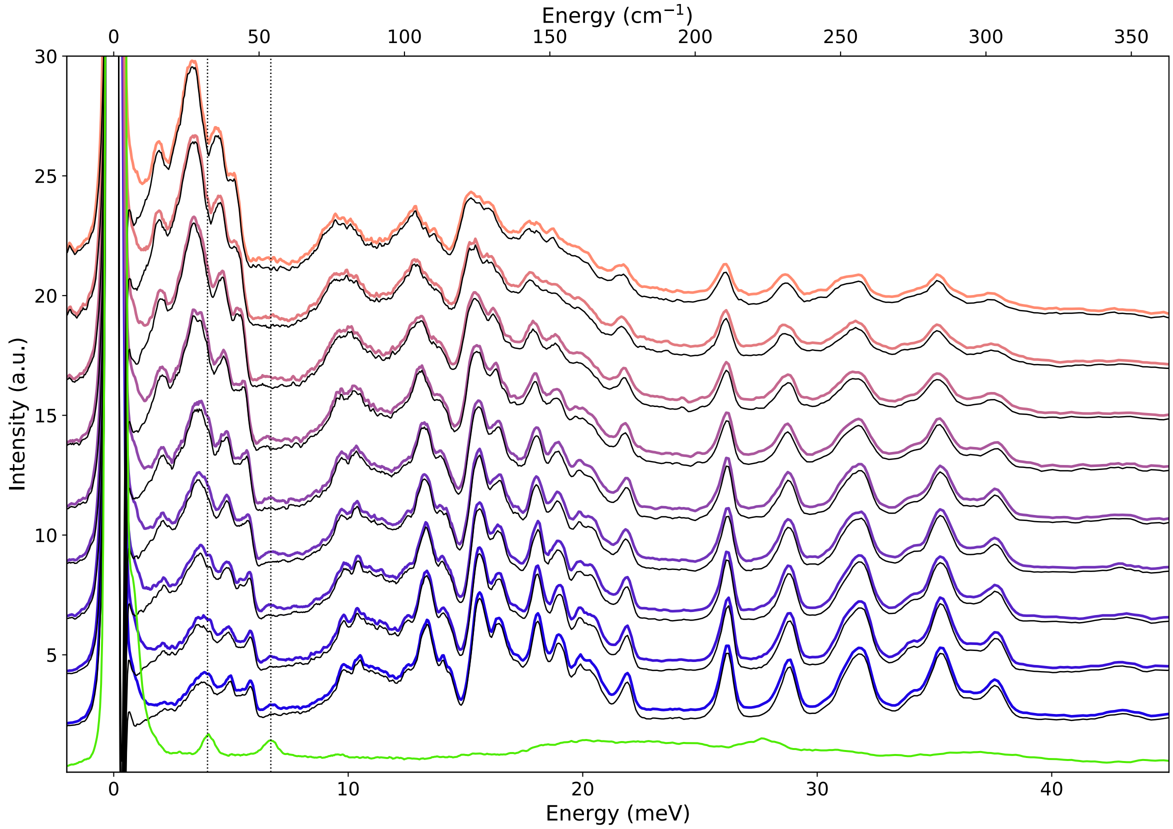

Figure S27. *Background subtraction for CuPc. Subtracted spectra (black curves) are obtained by subtracting the empty-can measurements at 5K (green curves, scaled by 5x for clarity) from the raw spectra (color curves). Dotted lines highlight spectral features of the empty-can signal that are removed from the sample spectra.*

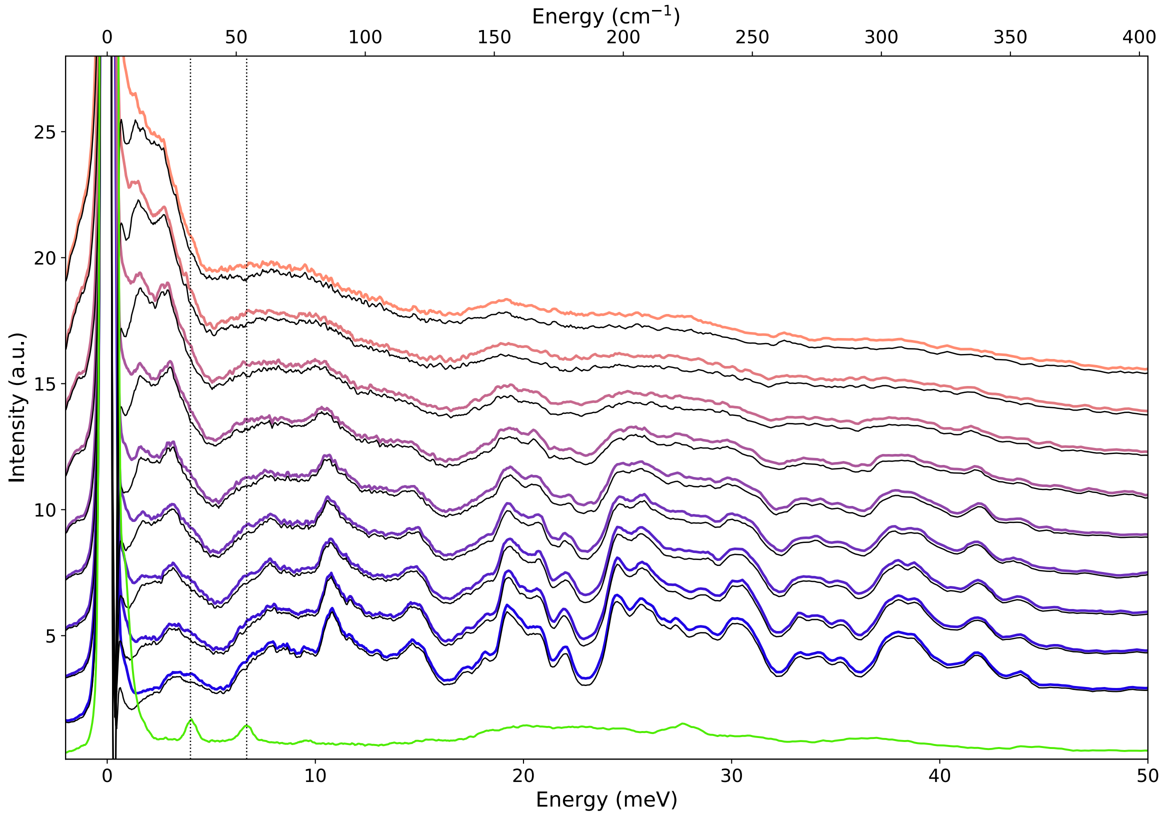

Figure S28. *Background subtraction for CuOEP. Subtracted spectra (black curves) are obtained by subtracting the empty-can measurements at 5K (green curves, scaled by 5x for clarity) from the raw spectra (color curves). Dotted lines highlight spectral features of the empty-can signal that are removed from the sample spectra.*

4.3 Bose correction

Phonons are bosonic quasiparticles whose population follows the Bose–Einstein distribution $n(E,T)$. Their excitation probability increases with temperature and is larger at lower energies because it depends on the number of phonons already thermally occupied. To account for this temperature-dependent population factor, all spectra were corrected by the factor $(n(E,T)+1)^{-1}$. This Bose correction removes the thermal population effect, allowing direct comparison of spectra collected at different temperatures and emphasizing the intrinsic phonon density of states, as shown in **Figure S29** and **Figure S30**.

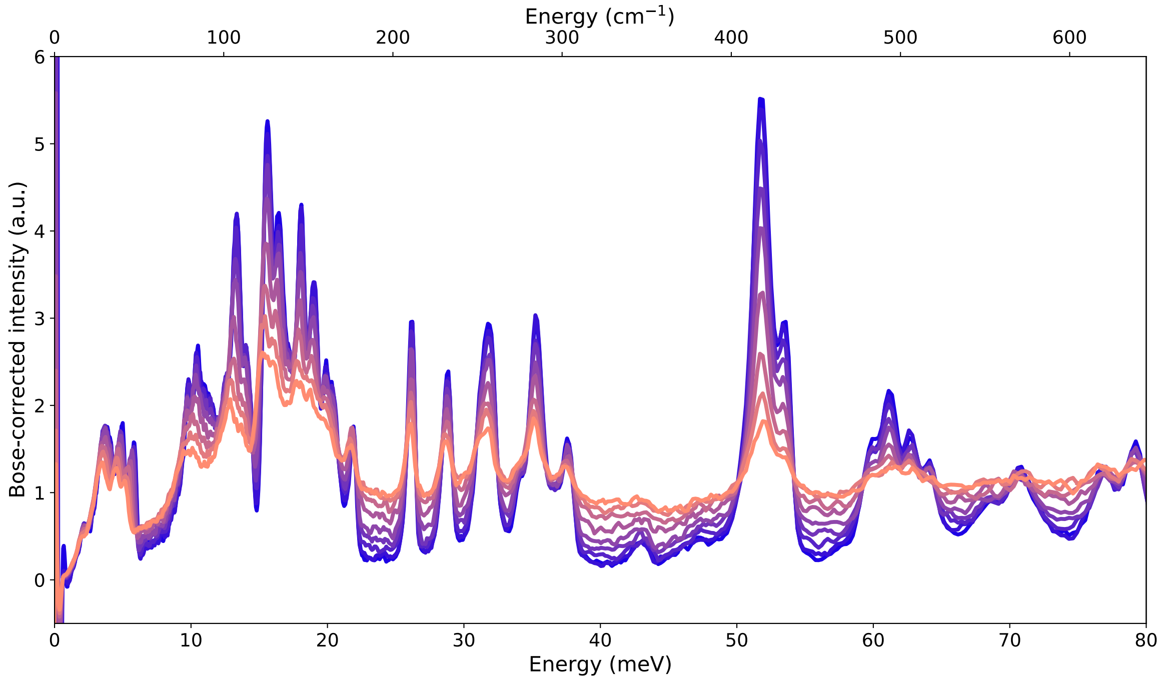

Figure S29. *Bose-corrected spectra of CuPc (after background subtraction).*

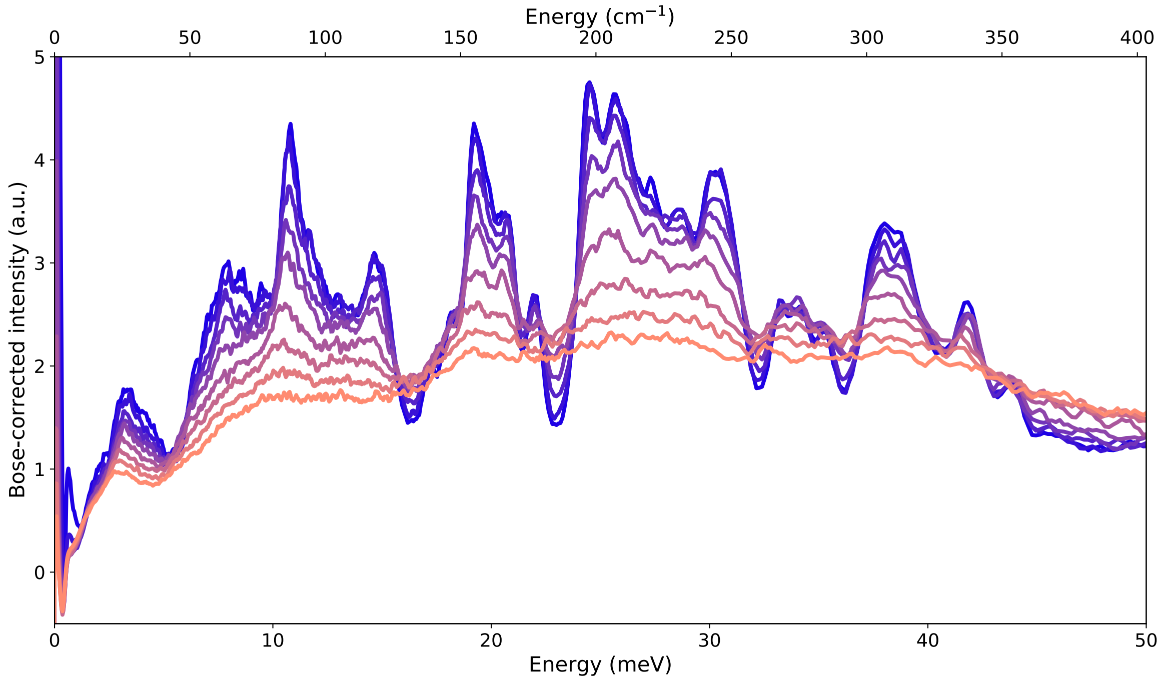

Figure S30. *Bose-corrected spectra of CuOEP (after background subtraction).*

4.4 Multiphonon correction

We aim to measure the phonon density of states, which represents the energy distribution of vibrations detected through one-phonon scattering events. However, incident neutrons can also excite two or more phonons simultaneously, producing additional spectral contributions (mainly at higher energies) that do not correspond to the intrinsic density of states but rather to combinations of multiple excited modes. These higher-order processes can be recursively calculated from the one-phonon contribution. **Figure S31** and **Figure S32** show the measured spectra at 5K, the calculated one-phonon and higher-order contributions. The multiphonon spectra are largely featureless, and their probability increases with excitation energy. For CuPc, the multiphonon contribution in the thermally accessible region below 100 meV is small, whereas CuOEP exhibits a substantial multiphonon signal even below 50 meV. The measured spectra were therefore corrected by subtracting the total calculated multiphonon contribution (green curves). Calculations were performed at 0 K, so the corrections do not include temperature-induced multiphonon contributions, which are expected to increase with temperature. We partially attribute the temperature-enhanced background signal observed between vibrational features (for example, in Figure S29 between 40-50 meV for CuPc) to these thermally activated multiphonon scattering events.

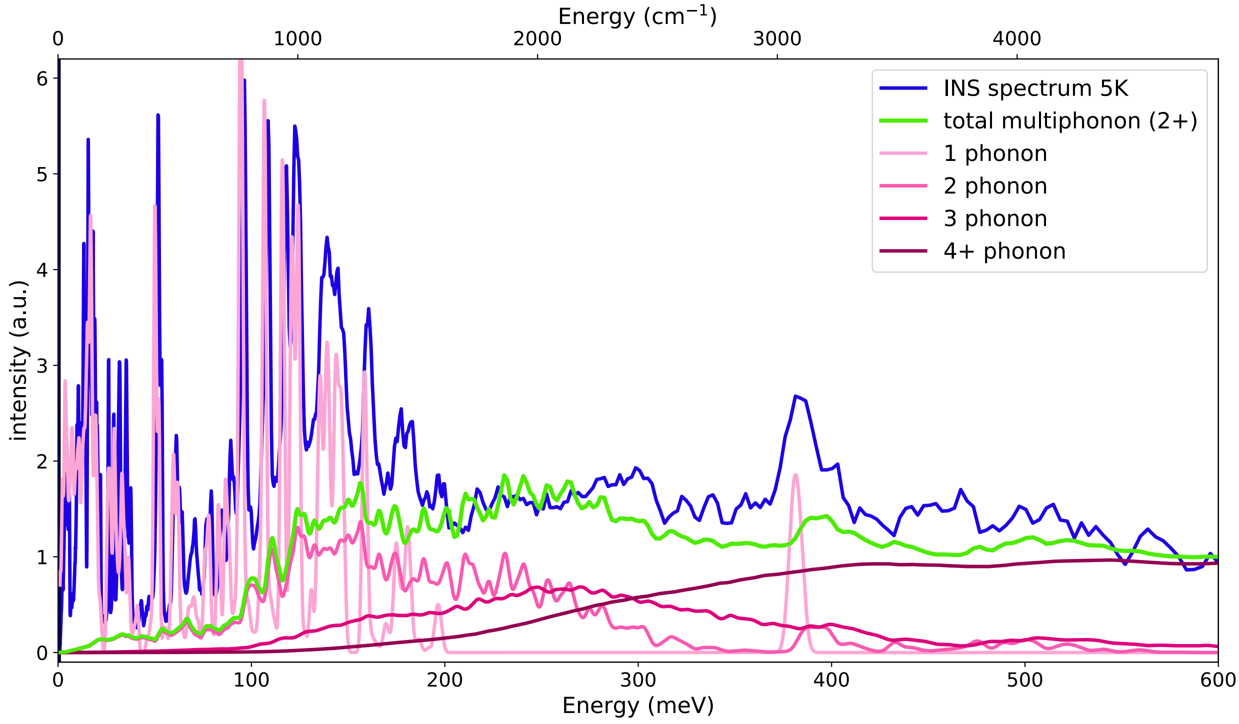

Figure S31. *Multiphonon contribution in CuPc. Calculated one-phonon and higher-order excitations are shown as pink-shaded curves. The total multiphonon contribution (sum of ≥ 2-phonon scattering events, green curve) was subtracted from the measured spectra (blue curve, only 5 K data is shown here).*

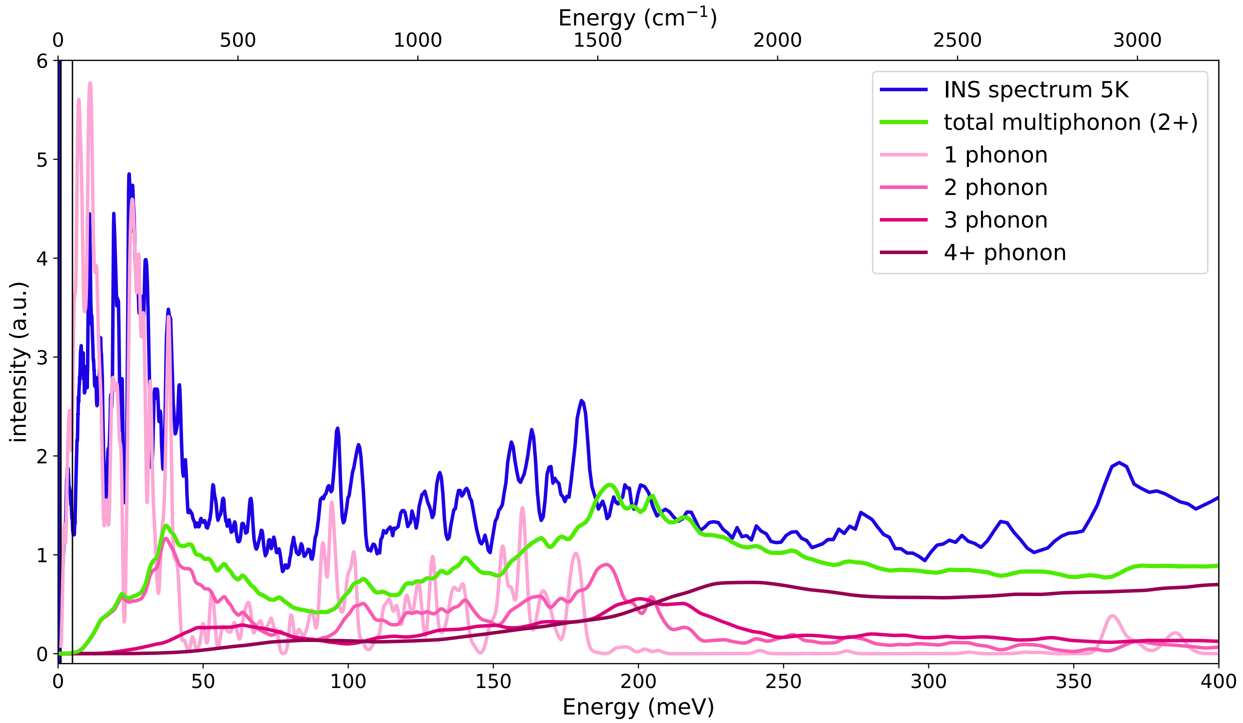

Figure S32. *Multiphonon contribution in CuOEP. Calculated one-phonon and higher-order excitations are shown as pink-shaded curves. The total multiphonon contribution (sum of ≥ 2-phonon scattering events, green curve) was subtracted from the measured spectra (blue curve, only 5 K data is shown here).*

4.5 Elastic line subtraction

The final step in obtaining the one-phonon spectrum is to remove the elastic peak from the data. At low energies (<10–15 cm^{-1}), we assume a Debye model, where the phonon density of states scales quadratically with energy (corresponding to a linear phonon dispersion). The lowest-energy phonon features were identified in the raw spectra, and cutoff energies were set below those features. The Debye model extrapolates the low-energy behavior of CuPc well, as shown in **Figure S33** (left). For CuOEP (right), the spectrum at the highest temperature exhibits an approximately linear phonon density, consistent with enhanced low-energy modes, as also observed in other porphyrins (11). For consistency across datasets, we applied the Debye model to all temperatures. After elastic-line subtraction, all spectra were normalized to unity up to 75 meV (≈600 cm^{-1}), which is taken here as the thermally accessible energy threshold. The corrected and normalized spectra are presented in the main text.

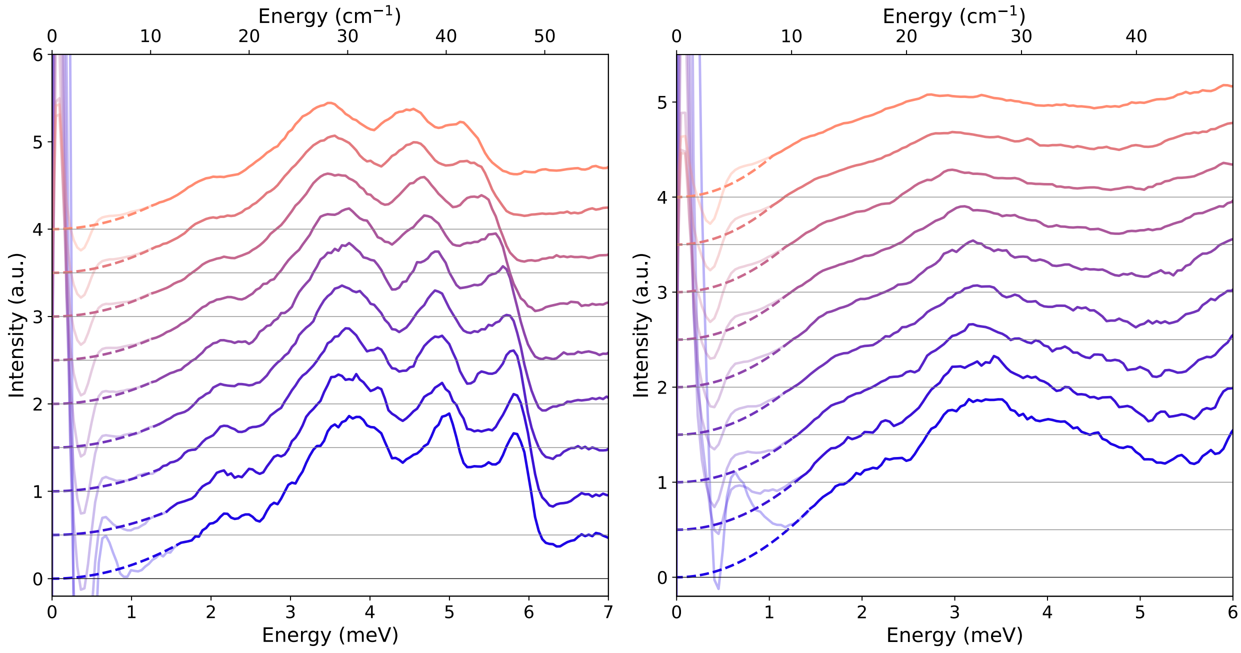

Figure S33. *Elastic line removal and low-energy Debye extrapolation for CuPc (left) and CuOEP (right).*

4.6 Phonon DOS

Inelastic neutron scattering measures the self-dynamic structure factor *S(Q,ω)*, which contains information on inelastic scattering processes and their cross sections. Within the incoherent approximation, the phonon density of states *G(ω)* is related to the measured *S(Q,ω)* as (12):

$$G(\omega) = \frac{2m}{e^{\langle -Q^2 x^2 \rangle}\hbar} \cdot \frac{\omega}{Q^2} \cdot \frac{S(Q,\omega)}{n(\omega)+1} \ ,$$

where $\omega = E/\hbar$ is the neutron energy transfer and *Q* the momentum transfer. The first term includes the exponential Debye–Waller (DW) factor, and the denominator in the last term accounts for the Bose population factor discussed above. For direct-geometry instruments with sufficient detector coverage, a 2D S(Q,ω) map can be measured. For each Q slice the DW factor is effectively constant, such that the density of states can be computed directly. VISION, however, is an indirect-geometry instrument that measures *S(Q,ω)* along a given *Q–ω* trajectory, so $Q = Q(\omega)$ and $S(Q,\omega) = S_{VISION}(\omega)$. To rigorously compute the phonon DOS, the DW factor must therefore be evaluated explicitly.

The DW factor can be easily computed when assuming a single effective mean-square atomic displacement $\langle x^2 \rangle$, corresponding to an average over all atoms, thereby omitting explicit atom-specific displacement amplitudes. This approximation is appropriate for computing the *neutron-weighted* DOS, where the scattering cross section is dominated by hydrogen atoms. An energy-dependent transfer function between *S(Q,ω)* and $G_{NW}(\omega)$ can thus be computed and is shown as the pink curves in **Figure S34**.

Our phonon calculations yield the VISION spectra $S_{VISION}(\omega)$ (shown in Fig. 3 of the main text), the phonon DOS *G(ω),* as well as the neutron-weighted DOS $G_{NW}(\omega)$. We can therefore alternatively compute transfer functions between $S_{VISION}(\omega)$ and $G_{NW}(\omega)$ or *G(ω)* by taking ratios of these calculated spectra. For the neutron-weighted DOS, the ratio $G_{NW}(\omega)/S_{VISION}(\omega)$ (black curve in **Figure S34**) agrees well with the theoretical expressions from above, particularly at low energies. Some additional spectral features are observed compared to the theoretical prediction obtained using a single effective $\langle x^2 \rangle$.

For computing the full DOS *G(ω)* mode-resolved contributions from different atomic species must be accounted for in the DW factor, and a purely analytical transfer function cannot be obtained without a full phonon calculation. We therefore compute the full DOS only via the ratio of the calculated spectra (gray curves).

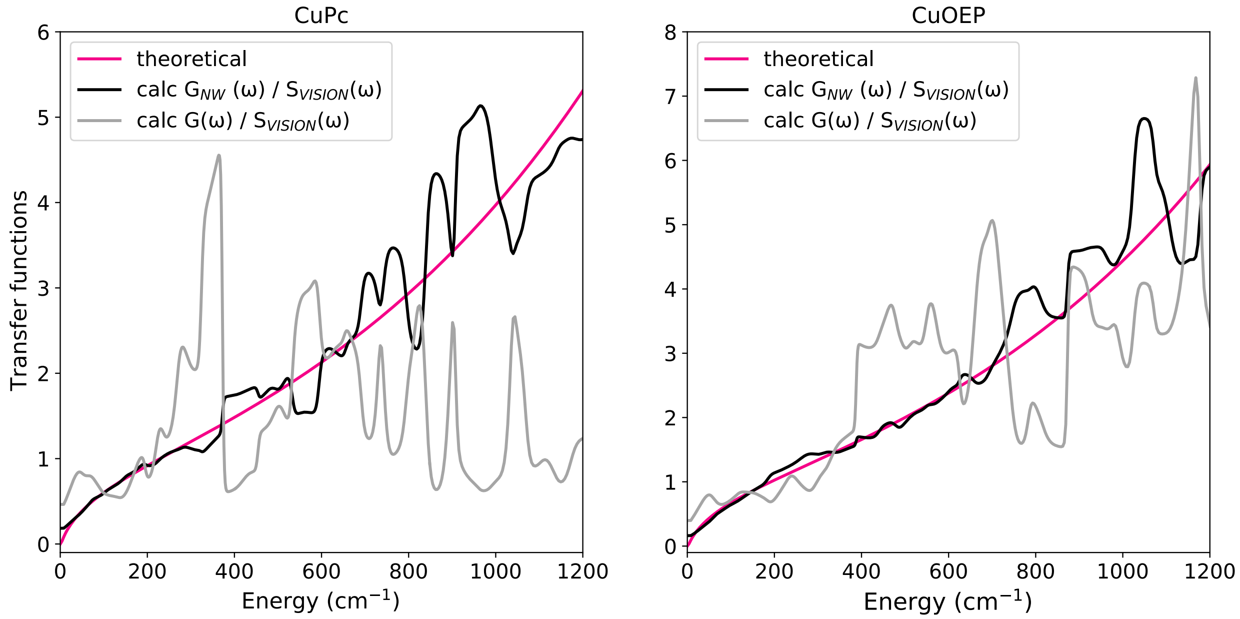

Figure S34. *Transfer functions between $S_{VISION}(\omega)$ and the phonon DOS $G(\omega)$ or the neutron-weighted phonon DOS $G_{NW}(\omega)$. Left: CuPc, right: CuOEP. Pink curves show the theoretical $S_{VISION}(\omega \leftrightarrow G_{NW}(\omega)$ transfer function computed using a single effective $\langle x^2 \rangle$ to evaluate the Debye-Waller factor. Black and gray curves were obtained by taking ratios of calculated spectra, as described in the text.*

To better visualize these transfer functions and their effect on the phonon spectra, **Figure S35** and **Figure S36** compile the calculated spectra for CuPc and CuOEP, their corresponding ratios (i.e. transfer functions) up to 600 cm^{-1}, as well as their effects on the experimentally measured $S_{VISION}(\omega)$. The lower panels show $S_{VISION}(\omega)$ (colored curves), together with $G_{NW}(\omega)$ and $G(\omega)$ obtained by applying the corresponding transfer functions. All spectra are normalized to unity up to 600 cm^{-1}.

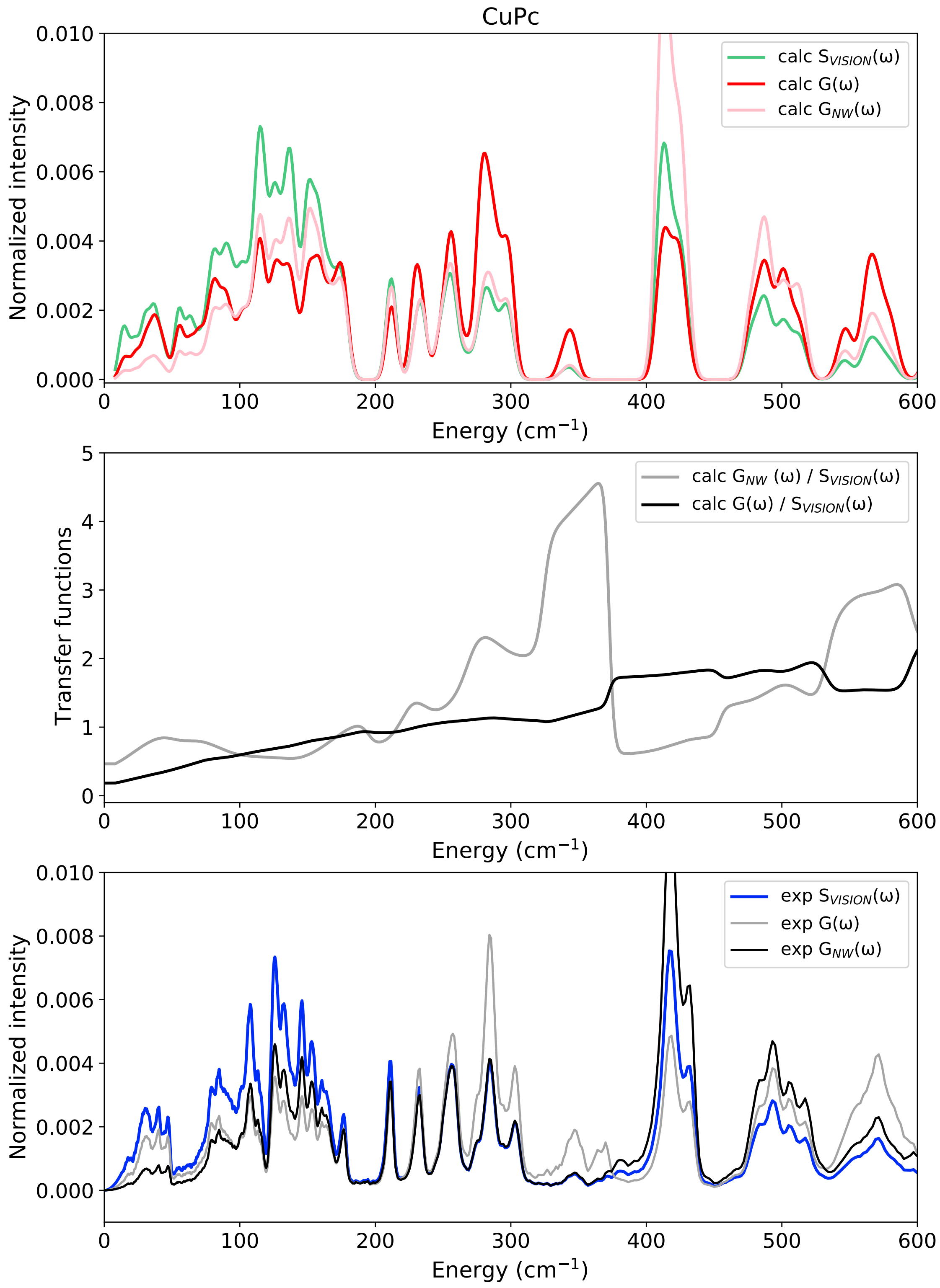

Figure S35. *Computational phonon DOS G(ω) and neutron-weighted DOS $G_{NW}(\omega)$ of CuPc from measured $S_{VISION}(\omega)$. Top: calculated 1-phonon VISION spectrum $S_{VISION}(\omega)$, G(ω), and $G_{NW}(\omega)$. Middle: transfer functions $S_{VISION}(\omega)\leftrightarrow G(\omega)$ (gray) and $S_{VISION}(\omega)\leftrightarrow G_{NW}(\omega)$ (black) obtained from ratios of calculated spectra. Bottom: experimental $S_{VISION}(\omega)$ after all corrections described in previous sections (blue curve), together with G(ω) and $G_{NW}(\omega)$ obtained by applying the corresponding transfer functions to the experimental spectrum.*

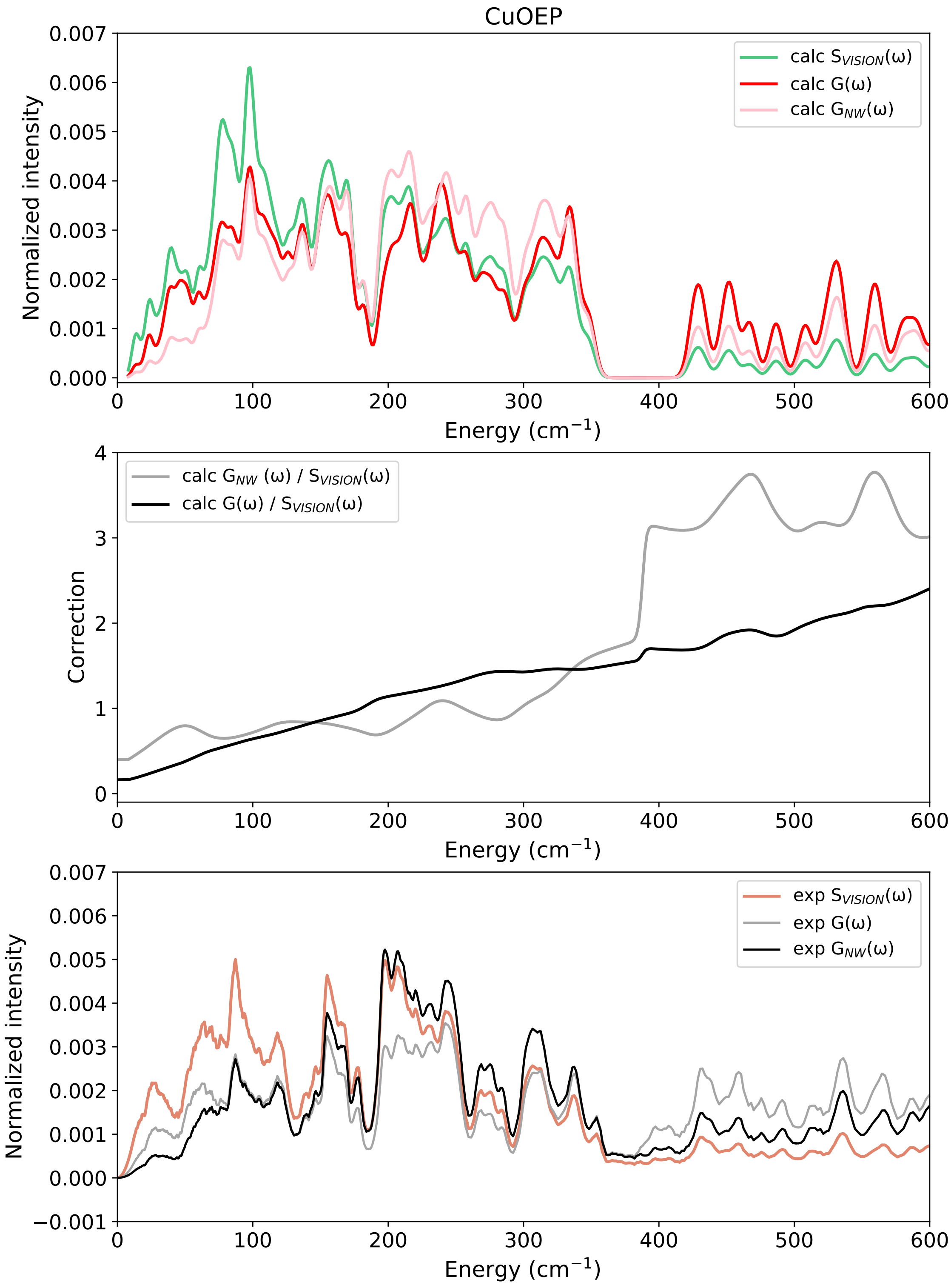

Figure S36. *Computational phonon DOS $G(\omega)$ and neutron-weighted DOS $G_{NW}(\omega)$ of CuOEP from measured $S_{VISION}(\omega)$. Top: calculated 1-phonon VISION spectrum $S_{VISION}(\omega)$, $G(\omega)$, and $G_{NW}(\omega)$. Middle: transfer functions $S_{VISION}(\omega)\leftrightarrow G(\omega)$ (gray) and $S_{VISION}(\omega)\leftrightarrow G_{NW}(\omega)$ (black) obtained from ratios of calculated spectra. Bottom: experimental $S_{VISION}(\omega)$ after all corrections described in previous sections (orange curve), together with $G(\omega)$ and $G_{NW}(\omega)$ obtained by applying the corresponding transfer functions to the experimental spectrum.*

The neutron-weighting correction skews the spectra almost monotonically, suppressing low-energy modes and enhancing higher-energy contributions. In contrast, the full-DOS transfer function is more structured. Its spectral features have wider widths than those of individual phonon modes, however. At low energies (below 200–300 cm^{-1}), the correction fluctuates around a constant value, resulting in relatively minor spectral reshaping. Stronger corrections appear at higher energies, leading to enhanced intensity in *G(ω)*. In particular, the optical modes of CuPc near 300 cm^{-1} are amplified, while modes in CuOEP above 400 cm^{-1} gain intensity. These reduce the low-energy spectral weight upon normalization (see next section).

For the calculation of SPC, the full phonon DOS containing contributions from all atomic species is preferable to the neutron-weighted DOS that has a skewed spectrum. However, as discussed above, the transfer functions required to obtain the full DOS exhibit stronger spectral features that can distort the experimental data and may introduce computational artifacts. To avoid imposing assumptions about which spectrum most accurately represents the true phonon DOS, we compute the SPC coefficients using all three spectral representations and compare their results (see Section 7.1). The main text reports only the experimentally measured $S_{VISION}(\omega)$, incorporating minimal computational input.

4.7 Normalization

For calculating the SPC coefficients, we require a phonon density of states that is normalized to unity. Since the choice of cutoff energy used for normalization directly affects the spectral intensities, its influence is discussed here. A cutoff energy is introduced to prevent high-energy spectral features, measured at reduced energy resolution on VISION, from artificially influencing the low-energy spectral intensities relevant for the SPC analysis. We are interested in processes occurring up to 300 K; therefore, a cutoff energy of 600 cm^{-1} safely includes all thermally accessible phonon modes at room temperature that might influence T_1 (the corresponding thermal population is shown in the next section). However, as discussed in the main text, the spectral features most relevant for SPC lie below 380 cm^{-1}, and phonon populations above this energy are minimal. Both CuPc and CuOEP exhibit a natural phonon gap near this energy, making 380 cm^{-1} an appropriate alternative cutoff. **Figure S37** compares the measured VISION spectra $S_{VISION}(\omega)$, the phonon DOS *G(ω),* and the neutron-weighted DOS $G_{NW}(\omega)$ obtained using these two normalization cutoffs.

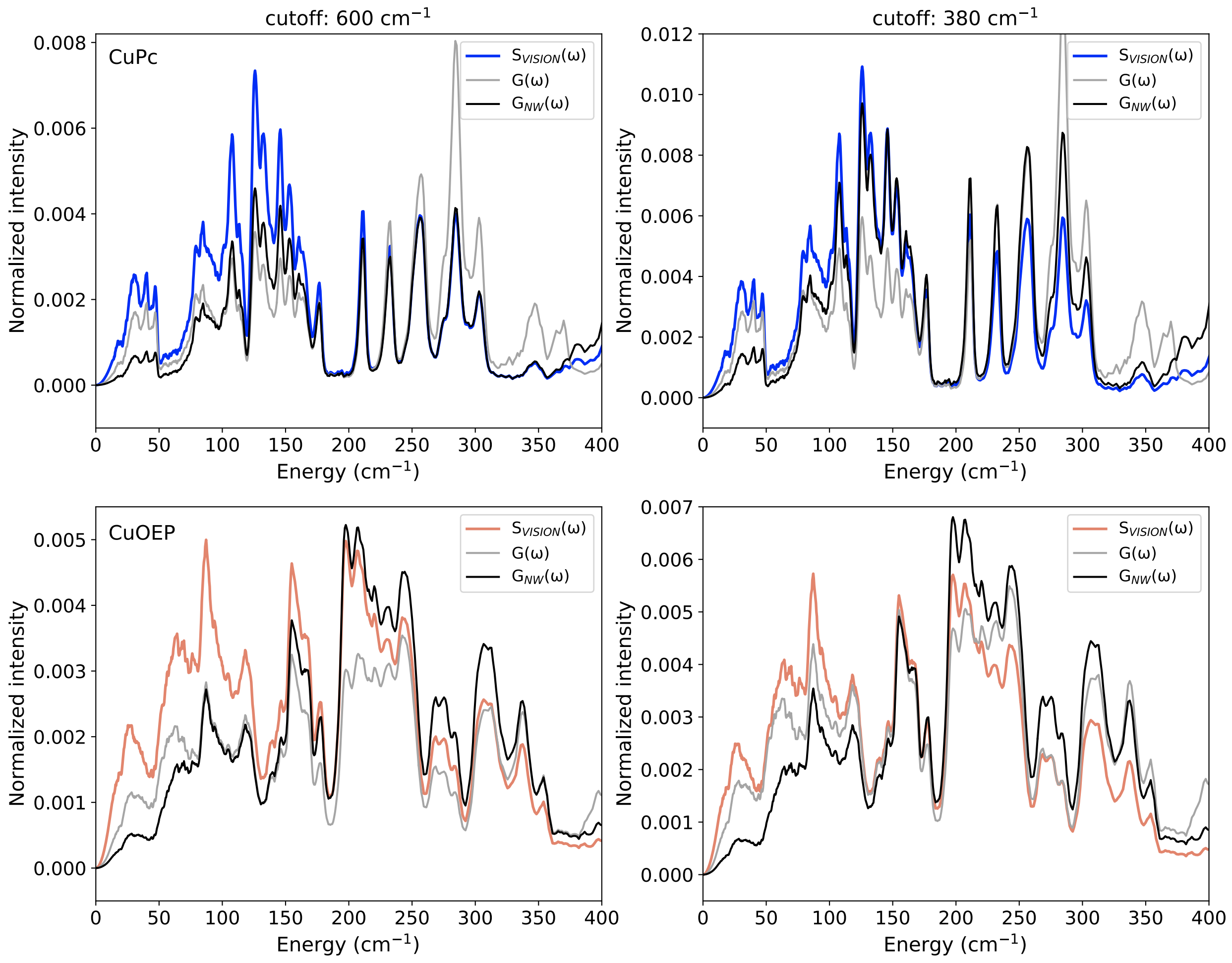

Figure S37. *Effects of normalization cutoff energy on the experimentally measured $S_{VISION}(Q,\omega)$, the phonon DOS $G(\omega)$, and the neutron-weighted DOS $G_{NW}(\omega)$. Left: E_{cutoff} =600 cm^{-1}. Right: E_{cutoff} =380 cm^{-1}. Top: CuPc. Bottom: CuOEP.*

When normalizing up to 600 cm^{-1}, the presence of additional spectral features at higher energies reduces the overall spectral intensities. This effect is more pronounced for CuPc, which exhibits sharper phonon features. The relative intensities between $S_{VISION}(\omega)$, $G(\omega)$, and $G_{NW}(\omega)$ also change, as the transfer functions used to obtain these spectra redistribute spectral weight in different ways. In CuOEP, these relative intensities are more strongly affected by the normalization choice than in CuPc. In particular, the intensity of $G(\omega)$ (gray curve) agrees significantly better with the measured spectrum $S_{VISION}(\omega)$ when normalized up to 380 cm^{-1} (a similar effect is observed for CuPc below 50 cm^{-1}).

This behavior indicates that normalization up to 380 cm^{-1} yields a full DOS that is more similar to the measured VISION spectrum than normalization up to 600 cm^{-1}. Since all SPC-relevant processes involve phonons below 380 cm^{-1}, this cutoff is appropriate for the present study, as it excludes effects from spectral features that are unlikely to participate in spin–phonon coupling. Nevertheless, in Section 7.2 we explicitly quantify the effect of the normalization choice on the computed SPC coefficients.

5 Thermal phonon population

To quantify and visualize the energies of the phonon spectrum that are thermally accessible at different temperatures, we weight the spectra measured at VISION, $S_{VISION}(E)$, by the Bose–Einstein occupation factor, $n(E,T)$. **Figure S38** and **Figure S39** show the resulting phonon populations of CuPc and CuOEP at the temperatures measured by INS.

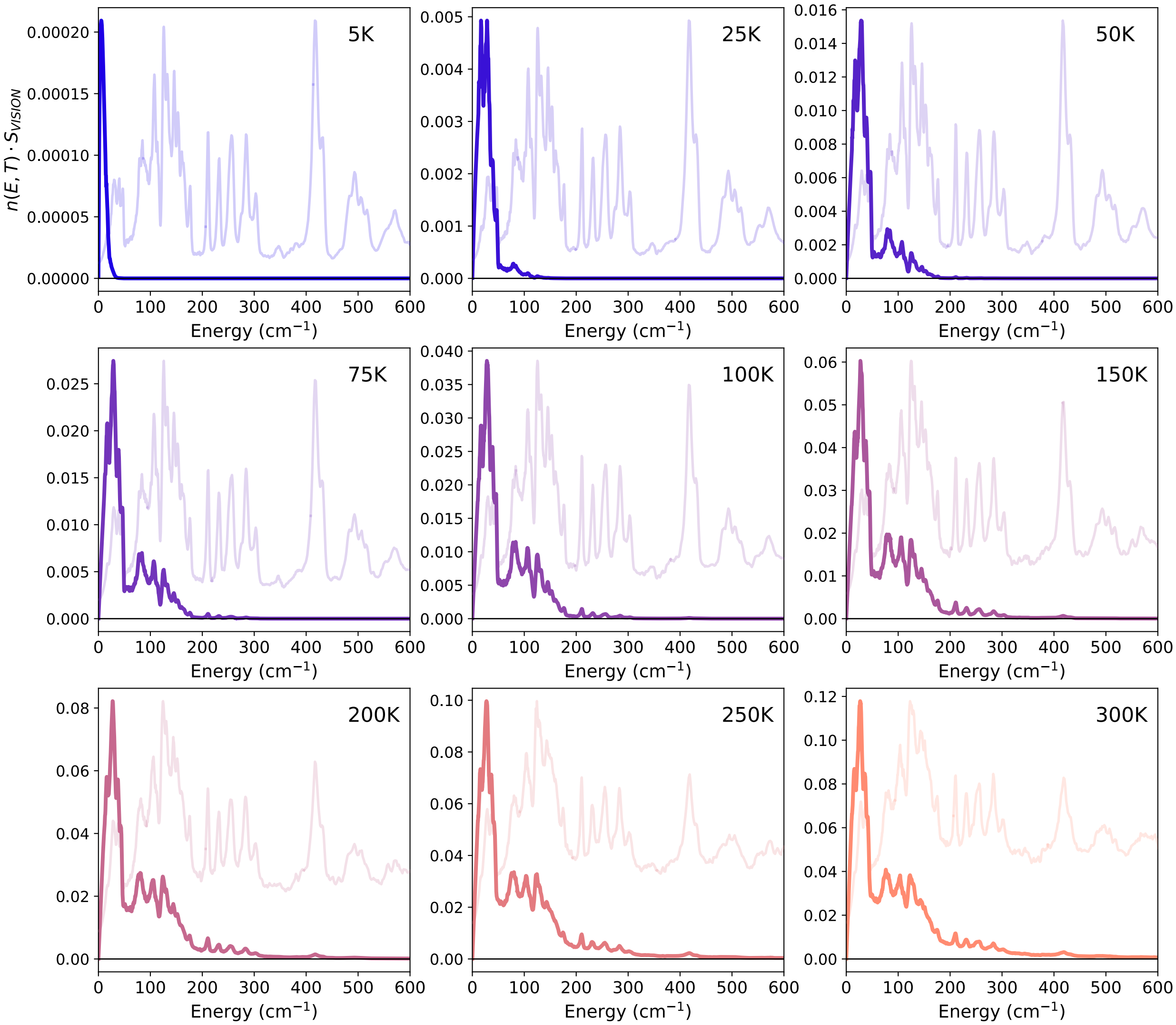

Figure S38. *Phonon thermal population of CuPc. The weighted phonon spectra, $n(E,T) \cdot S_{VISION}$, are overlaid with the measured spectra S_{VISION} (light curves, no axis) for reference.*

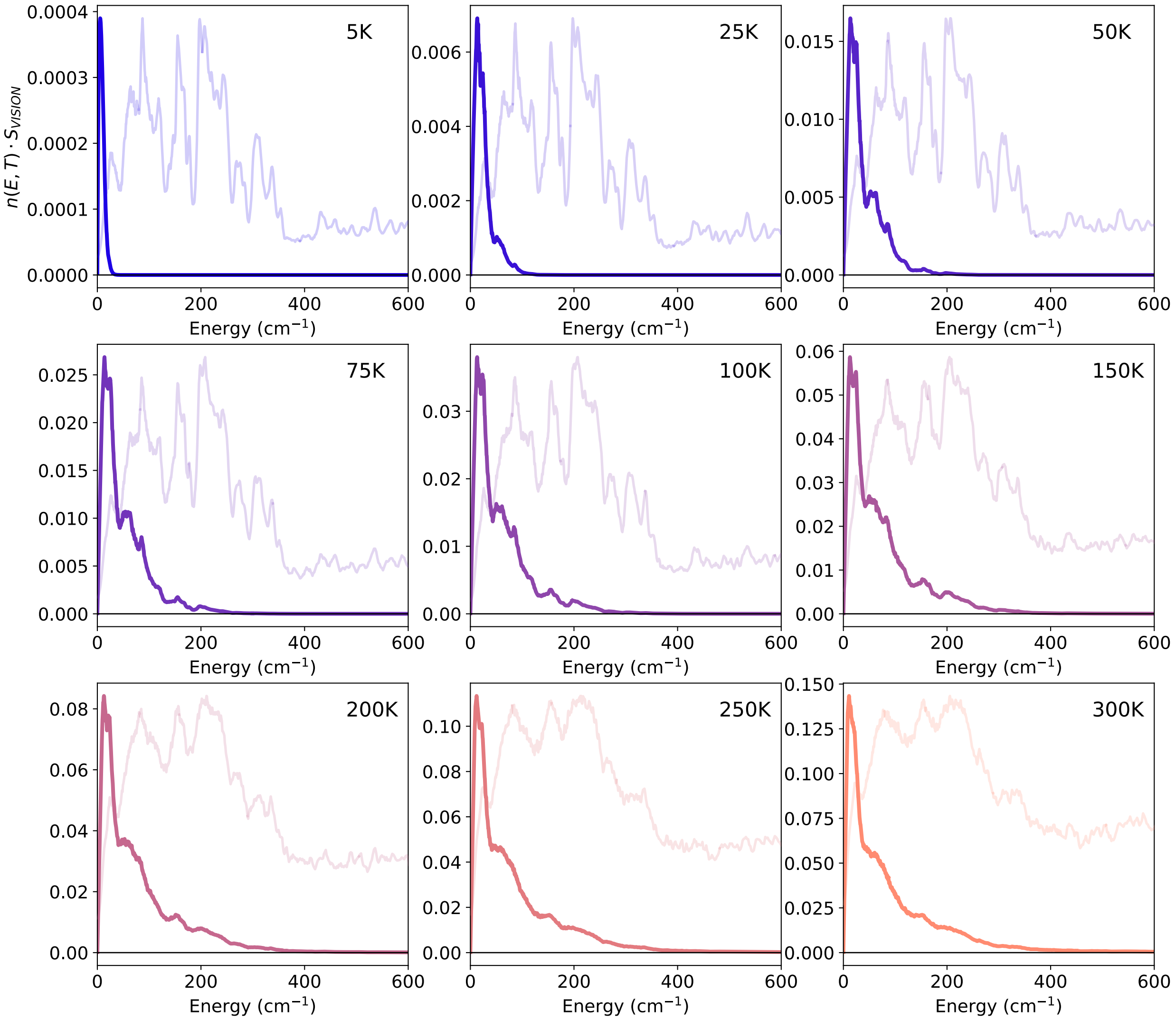

Figure S39. *Phonon thermal population of CuOEP. The weighted phonon spectra, n(E,T)·S_{VISION}, are overlaid with the measured spectra S_{VISION} (light curves, no axis) for reference.*

6 Phonon Anharmonicities

The positions and widths of the phonon peaks were obtained by fitting Gaussian functions to the measured spectra (and Voigt functions for the low-energy CuPc peaks below 7 meV). Overlapping features were modeled as sums of these profiles. Fits for CuPc are shown in **Figure S40**. Because of the sharpness of the CuPc spectra, we obtained reliable fits up to room temperature. The extracted phonon energy shifts and linewidth broadenings (changes in peak FWHM, Γ) are summarized in **Figure S41**. Lower-energy modes (darker shades) exhibit larger redshifts. In contrast, higher-energy modes show the greatest linewidth broadening, consistent with shorter phonon lifetimes ($\tau \approx \hbar/\Gamma$).

Within the quasiharmonic approximation (QHA), phonon frequencies do not scale directly with temperature but vary linearly with volume: $E_{ph}(T) = E_{ph}\big(V(T)\big)$. Consequently, phonon shifts are driven by thermal expansion. The mode Grüneisen parameter is defined as

$$\gamma_i = -\frac{V}{E_i}\frac{\partial E_i}{\partial V}$$

a dimensionless measure of the fractional energy change with fractional volume change, or how strongly phonons respond to an expanding lattice. We measured lattice volume expansion by *in situ* neutron diffraction to determine the fractional volume change, $\Delta V/V$ (see Section 0). As shown in **Figure S42** (center), the phonon energies correlate mostly linearly with changes in V, supporting the QHA description. Deviations from linearity indicate pure anharmonic effects (e.g., phonon–phonon or spin–phonon interactions) beyond QHA (13, 14).

The resulting mode-resolved Grüneisen parameters are largest for low-energy modes (**Figure S42**, right). At low temperatures, the fractional volume change $\Delta V/V$ is very small, and being in the denominator, this amplifies fitting uncertainties of the phonon energies and overestimates γ. We therefore consider the high temperature region above 100–150 K. For CuPc, the largest γ values are ≈ 4.

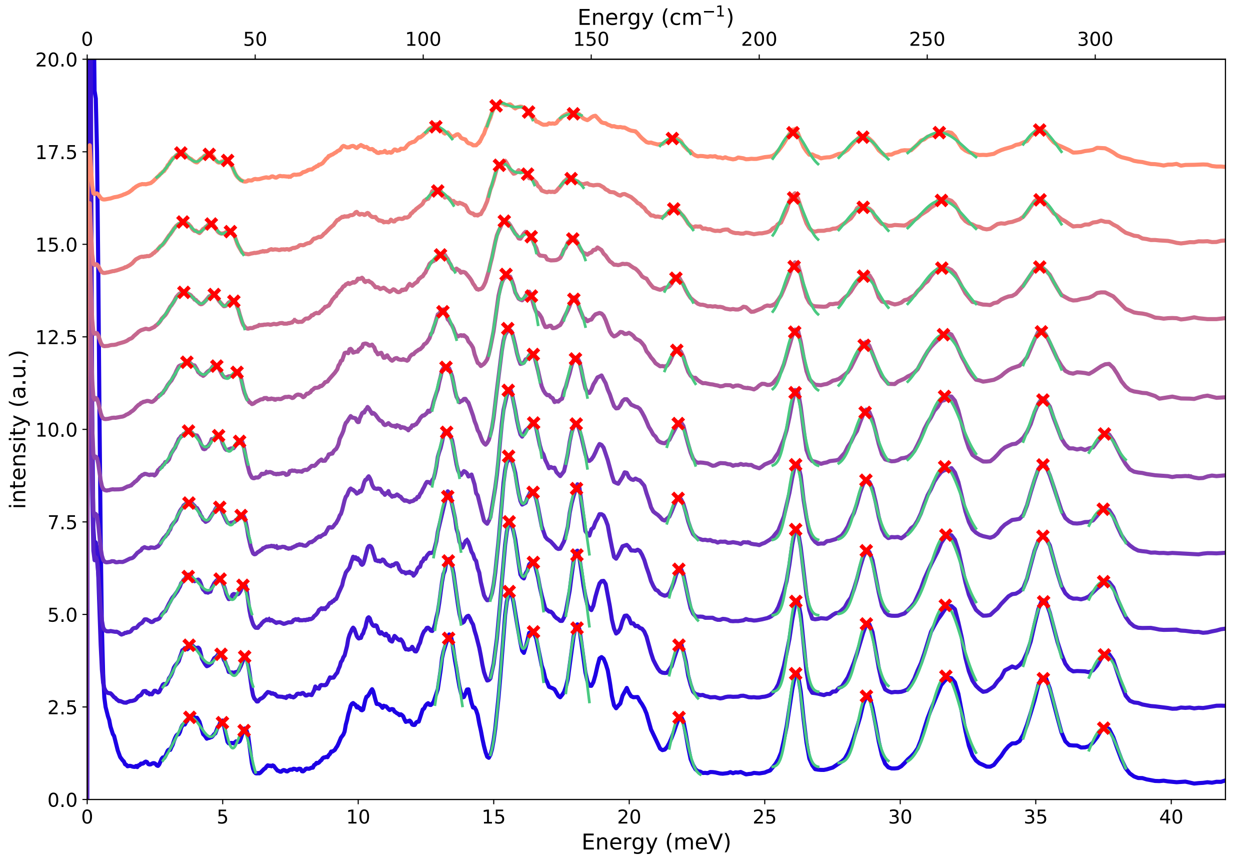

Figure S40. *Fitting of phonon peaks for CuPc. Green curves are fitted Gaussian functions (and Voigt profiles below 7 meV), red crosses are fitted maxima, corresponding to the phonon energies.*

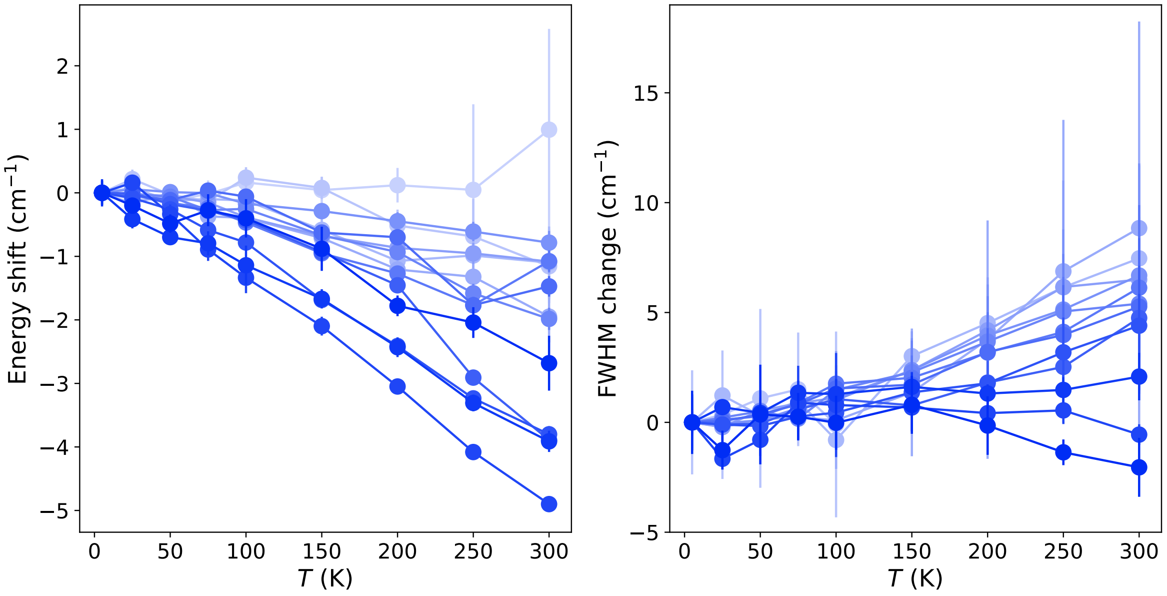

Figure S41. *Phonon energy shifts (left) and linewidth broadening (right) of CuPc, extracted from the data through fits of Figure S40. Blue shades from dark to light correspond to increasing phonon energies. Error bars represent one standard deviation from the peak fits.*

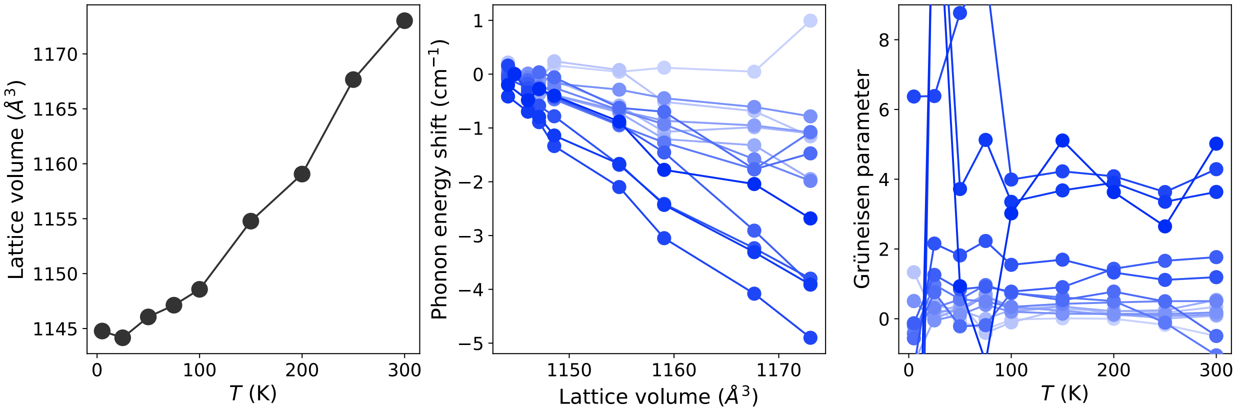

Figure S42. *Quasiharmonic energy shifts of CuPc. Left: Lattice volume measured by neutron diffraction by assuming an isotropic expansion (see section 0). Center: Phonon energy shifts vs. lattice volume, showing their mostly linear correlation. Right: Grüneisen parameters (blue shades from dark to light correspond to increasing phonon energies).*

The broader phonon peaks of CuOEP cause substantial overlap between modes, which prevents reliable fits at all temperatures. To enable comparison with CuPc, we fit the energy positions of the most pronounced CuOEP peaks with Gaussian functions. Most fits were not feasible at higher temperatures, and linewidths are not reported because they are unreliable. The fitted peak positions are shown in **Figure S43**, and their temperature dependence is visualized in **Figure S44.** Although these fits are more qualitative than for CuPc, they allow us to establish the trends discussed below.

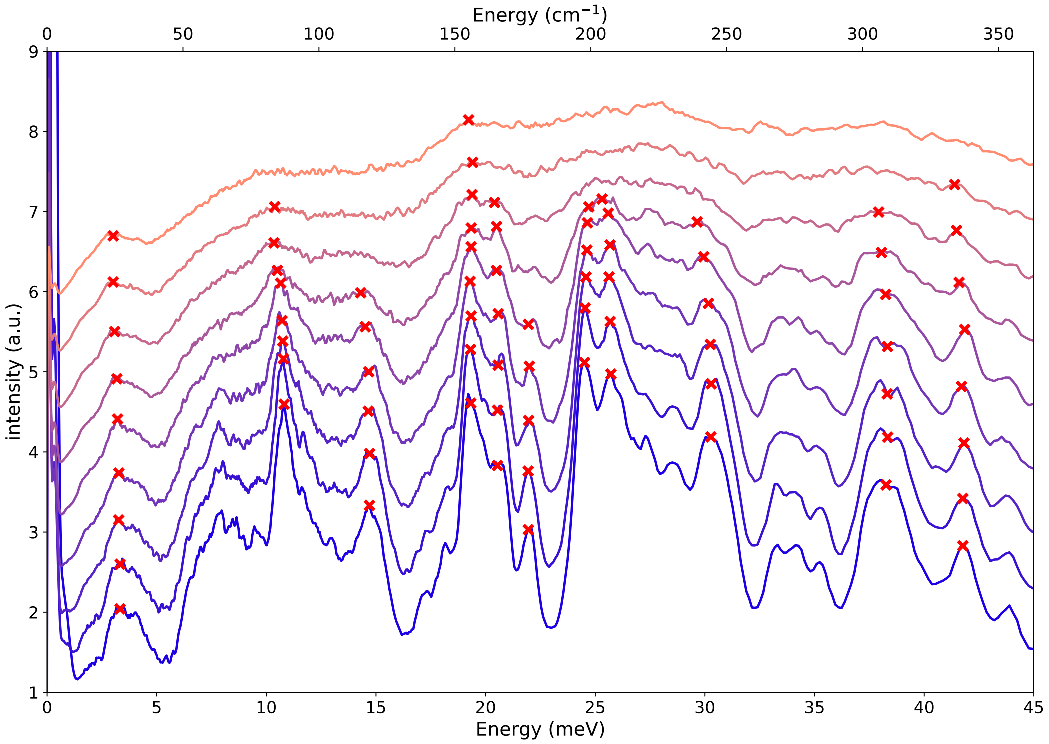

Figure S43. *Fitting of phonon peaks for CuOEP. Red crosses are fitted maxima, corresponding to the phonon energies.*

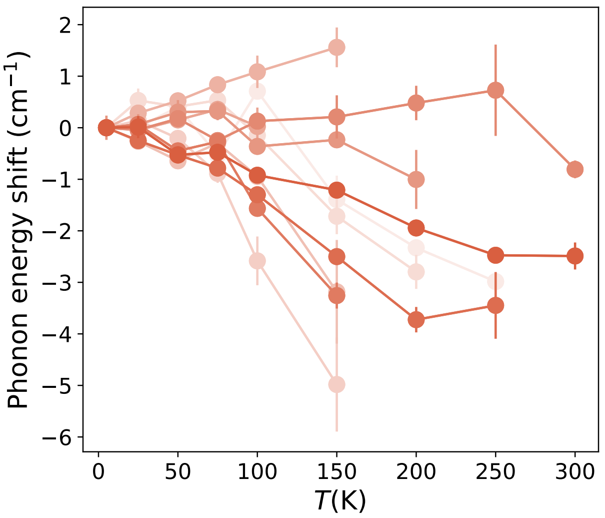

Figure S44. *Phonon energy shifts of CuOEP extracted from the data through fits of Figure S43. Orange shades from dark to light correspond to increasing phonon energies. Error bars represent one standard deviation from the peak fits.*

Low-energy modes (darker shades) in CuOEP exhibit smaller shifts than in CuPc, whereas some higher-energy modes (lighter shades) show more pronounced shifts, especially the feature near 240 cm^{-1} (which softens by ~5 cm^{-1} by 150 K). Consequently, even though CuOEP undergoes roughly twice the lattice expansion of CuPc, its low-energy mode Grüneisen parameters are smaller, about half as large, with the largest values around 2 above 150 K (**Figure S45**, right). Lower-energy modes yield larger γ with respect to high-energy ones with comparable absolute peak shifts because their fractional change $\Delta E/E$ is larger. This is consistent with these modes being the thermally active part of the spectrum that carries most of the heat and therefore has greater weight in quantities related to lattice expansion. The lower values of the Grüneisen parameters in CuOEP indicate that volume changes couple less strongly to its vibrational energies than in CuPc, at least for low-energy phonons.

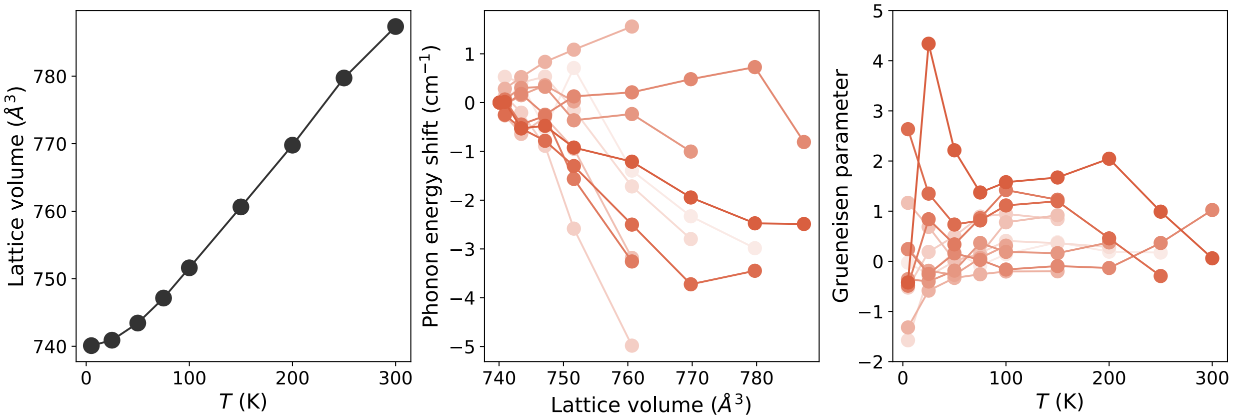

Figure S45. *Quasiharmonic energy shifts of CuOEP. Left: Lattice volume measured by neutron diffraction by assuming an isotropic expansion (see section 0). Center: Phonon energy shifts vs. lattice volume, showing their mostly linear correlation. Right: Grüneisen parameters (orange shades from dark to light correspond to increasing phonon energies).*

A direct comparison of phonon frequency shifts and Grüneisen parameters between CuPc and CuOEP is provided in **Figure S46**. The left panel shows that CuOEP's low-energy modes have a weaker dependence (shallower slopes) on lattice-size changes, corresponding to lower mode γ (center). In contrast, several high-energy modes in CuOEP shift more with temperature than in CuPc, most notably the optical mode near 240 cm^{-1} whose change of -5 cm^{-1} by 150 K is comparable to the largest shift in CuPc at 300 K. The Grüneisen parameters of such high-energy modes are therefore often larger in CuOEP than in CuPc. The right panel of Figure S46 shows γ_i at 150 K versus phonon energy, highlighting this dual behavior: while CuOEP's low-energy modes exhibit weaker quasiharmonic coupling to the lattice volume than in CuPc (lower γ_i), specific high-energy modes in CuOEP display enhanced frequency shifts resulting in larger γ_i. This suggests additional explicit anharmonicity beyond the QHA for certain high-energy modes in CuOEP, consistent with its broader linewidths (shorter phonon lifetimes) and the mixed character of its vibrations, which can also enhance phonon–phonon scattering (see main manuscript).

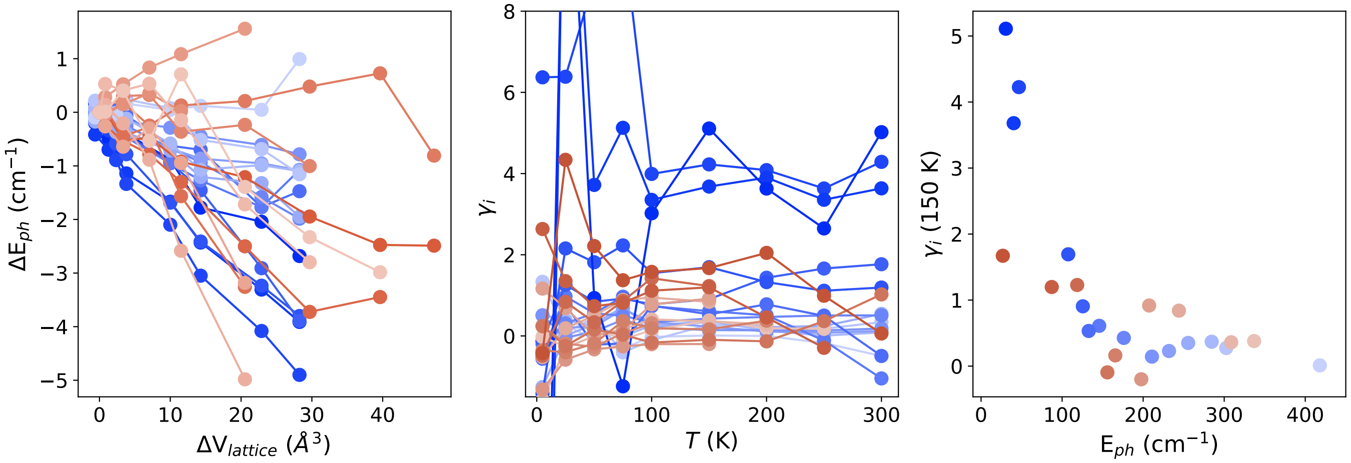

Figure S46. *Comparison of anharmonic phonon shifts in CuPc (blue) and CuOEP (orange). Left: Phonon energy shifts versus lattice-volume change. Center: Resulting Grüneisen parameters. Right: Grüneisen parameters at 150 K versus the corresponding phonon energies. Color shades from dark to light correspond to increasing phonon energies.*

7 Spin–Phonon Coupling Fits

To test the robustness of our model, which uses experimentally determined phonon spectra to fit SPC coefficients (λ_{SPC}), we explored variations of the fitting procedure presented in the main text. The model follows:

$$\left(\frac{1}{T_1}\right)_T = \int_0^{E_{ph,max}} \lambda_{SPC}(\varepsilon) \cdot G_T(\varepsilon) \cdot \frac{e^{\varepsilon/k_B T}}{(e^{\varepsilon/k_B T} - 1)^2} d\varepsilon \,,$$

where $G_T(\varepsilon)$ is the phonon density of states, the last term represents the thermal factor for two-phonon excitation, and $E_{ph,max}$ is the cutoff energy.

7.1 Dependence of phonon spectrum: *S_{VISION}(E)* vs. *G(E)* vs. *G_{NW}(E)*

As discussed in Section 4.6 the phonon spectrum measured with VISION corresponds to the self-dynamic structure factor *$S_{VISION}(E)$,* and not directly the phonon density of states. Using calculated spectra, we can construct transfer functions to compute the phonon DOS *G(E)* and the neutron-weighted DOS *$G_{NW}(E)$.* To minimize computational input, the main text presents SPC coefficients extracted solely from the experimental spectrum *$S_{VISION}(E)$*. Here, we want to quantify the effect of using these different phonon spectra on the low- and high-energy SPC coefficients (labelled λ_1 and λ_2 here). The coefficients are directly compared in **Table S6** and the corresponding fits and $1/T_1$ spectral densities are shown in **Figure S47** and **Figure S48**. $E_{ph,max}$ = 600 cm^{-1} was used here for all spectra.

		$S_{VISION}(E)$	*G(E)*	*$G_{NW}(E)$*
CuPc	λ_1	0.068	0.135	0.318
	λ_2	127.4	111.3	126.4
	λ_2/λ_1	1879	825	397
CuOEP	λ_1	0.033	0.082	0.175
	λ_2	20.47	25.86	17.88
	λ_2/λ_1	615	316	102

Table S6. *Dependence of the extracted SPC coefficients (in μs^{-1}) on the phonon spectrum used in the fitting procedure.*

For both samples, the low-energy SPC coefficient λ_1 is smallest when using *$S_{VISION}(E)$*. It increases by approximately a factor of two when using *G(E),* and by roughly a factor of 4–5 when using *$G_{NW}(E)$.* This behavior is expected, as both *G(E)* and especially *$G_{NW}(E)$* suppress spectral weight below 50 cm^{-1}, thereby requiring larger coupling coefficients to reproduce the experimental relaxation rates. The spectra *$S_{VISION}(E)$, G(E), and $G_{NW}(E)$*, were directly compared in **Figure S35** and **Figure S36***,* and are shown as light-shaded curves in **Figure S47** and **Figure S48**.

Neutron weighting is not desirable for computing SPC coefficients, as the coupling should account for vibrational contributions from all atoms in the molecule rather than being dominated by hydrogen, which controls $G_{NW}(E)$. In the low-energy regime, the experimentally measured $S_{VISION}(E)$ more closely resembles the full phonon DOS than the neutron-weighted one. SPC coefficients extracted using $G_{NW}(E)$ therefore provide an effective upper bound within our analysis framework.

The high-energy SPC coefficient λ_2 is significantly less sensitive to the choice of phonon spectrum, varying by at most 13% for CuPc and 31% for CuOEP. In CuPc, the DOS $G(E)$ enhances spectral weight near 300 cm^{-1}, leading to a reduction of λ_2.. In CuOEP, $G(E)$ suppresses and $G_{NW}(E)$ enhances intensities in the 200–350 cm^{-1} range, resulting in opposing trends in λ_2.
In all cases, the high-energy SPC coefficient is two to three orders of magnitude larger than the low-energy coefficient. The ratio λ_2/λ_1 decreases when progressing from $S_{VISION}(E)$ to $G(E)$ and to $G_{NW}(E)$, reflecting the progressive shift of spectral weight toward higher energies introduced by DOS and neutron-weighting corrections.

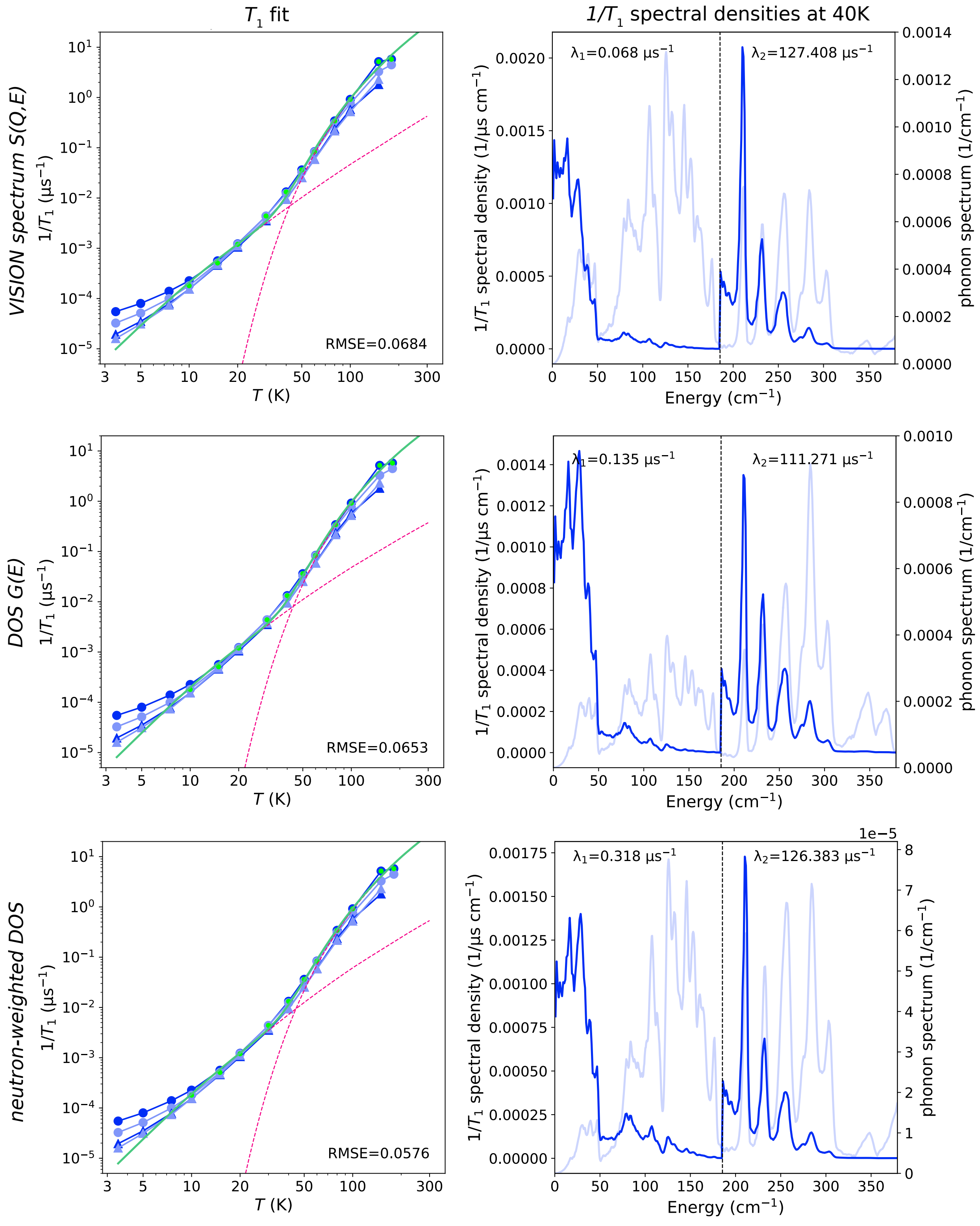

Figure S47. *Dependence of the spin-lattice relaxation fits of CuPc on the phonon spectrum used in the analysis. Top: VISION spectrum $S_{VISION}(E)$. Middle: phonon DOS G(E). Bottom: neutron-weighted phonon DOS $G_{NW}(E)$. Left: $1/T_1$ fits. Right: $1/T_1$ spectral densities overlaid with corresponding phonon spectrum (light shades).*

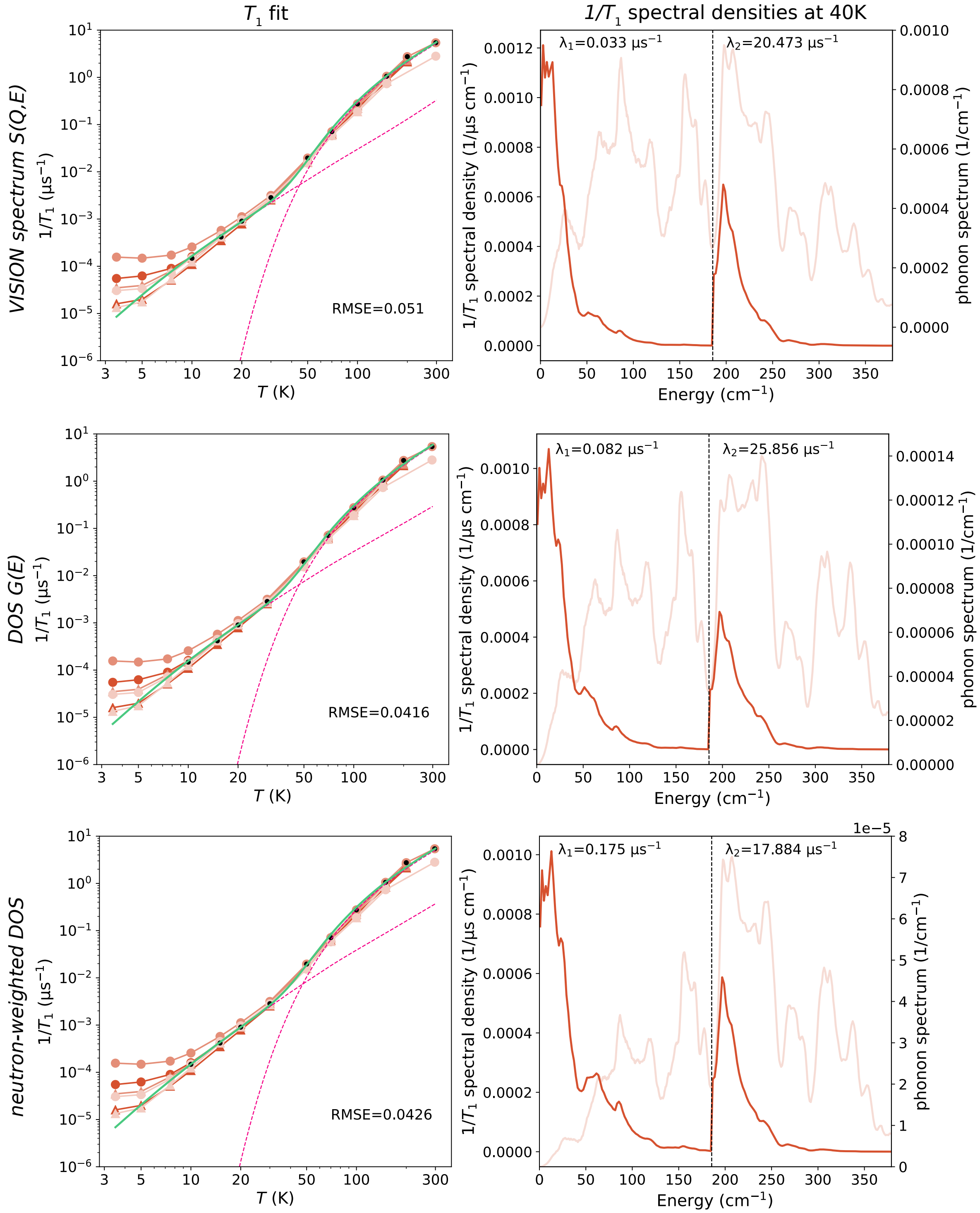

Figure S48. *Dependence of the spin-lattice relaxation fits of CuOEP on the phonon spectrum used in the analysis. Top: VISION spectrum $S_{VISION}(E)$. Middle: phonon DOS G(E). Bottom: neutron-weighted phonon DOS $G_{NW}(E)$. Left: $1/T_1$ fits. Right: $1/T_1$ spectral densities overlaid with corresponding phonon spectrum (light shades).*

7.2 Dependence of normalization cutoff energy

The cutoff energy chosen for normalization modifies the spectral intensities and therefore affects the values of the fitted SPC coefficients. As discussed in Section 4.7, two cutoff energies are appropriate for our analysis: 600 cm^{-1} and 380 cm^{-1}. **Table S7** summarizes the dependence of the fitted SPC coefficients on the choice of cutoff energy when using the experimental VISION spectrum, the phonon DOS, and the neutron-weighted DOS for the fits.

		Cutoff: 600 cm^{-1}			*Cutoff: 380 cm^{-1}*		
		$S_{VISION}(E)$	$G(E)$	$G_{NW}(E)$	$S_{VISION}(E)$	$G(E)$	$G_{NW}(E)$
CuPc	λ_1	0.068	0.135	0.318	0.045	0.081	0.148
	λ_2	127.4	111.3	126.4	94.55	72.91	70.94
	λ_2/λ_1	1879	825	397	2080	903	480
CuOEP	λ_1	0.033	0.082	0.175	0.028	0.051	0.132
	λ_2	20.47	25.86	17.88	17.46	16.60	13.19
	λ_2/λ_1	615	316	102	613	323	100

Table S7. *Dependence of cutoff energy on fitted SPC coefficients (in μs^{-1}). Coefficients were fit using the VISION spectra $S_{VISION}(E)$, the phonon DOS $G(E)$, and the neutron-weighted DOS $G_{NW}(E)$.*

Reducing the cutoff energy from 600 to 380 cm^{-1} excludes higher-energy spectral features, thereby increasing the relative intensities within the remaining energy window. Consequently, the extracted SPC coefficients decrease. This reduction is more pronounced for CuPc (26–55%) than for CuOEP (15–38%), reflecting the sharper phonon features of CuPc, which are more strongly affected by the inclusion or exclusion of spectral weight near the cutoff.

The magnitude of the reduction also increases when using $G(E)$ or $G_{NW}(E)$ instead of $S_{VISION}(E)$, since these spectral representations shift phonon weight toward higher energies that are removed by the lower cutoff. For CuPc, for example, the low-energy SPC coefficient λ_1 is reduced by 34% when using $S_{VISION}(E)$ and by 54% when using $G_{NW}(E)$. For CuOEP, the corresponding reductions are 15% and 25%, respectively.

It should be noted that these variations in the SPC coefficients are significantly smaller than the differences arising from the choice of phonon spectrum itself. Moreover, the relative strengths of the low- and high-energy coupling channels are largely preserved, as the ratios λ_2/λ_1 remain approximately constant upon changing the normalization cutoff.

7.3 Dependence of experimental field and EPR sequences

Here we compare fits to the T_1 data measured at different field strengths/orientations and acquired using both inversion-recovery and saturation-recovery pulse sequences. We use the same 185 cm^{-1} cutoff between low- and high-energy regions as in the main manuscript, and a normalization cutoff at 600cm^{-1}. Only data above 10 K were included, as the direct relaxation mechanism becomes important at lower temperatures. The fitted SPC coefficients for CuPc and CuOEP, together with the total RMSE evaluated in log-scale, are shown in **Figure S49** and **Figure S50**. The fits presented in the main text used saturation-recovery data below 30 K and inversion-recovery data above 30 K, both measured at the perpendicular field orientations (3390 G for CuPc and 3381 G for CuOEP).

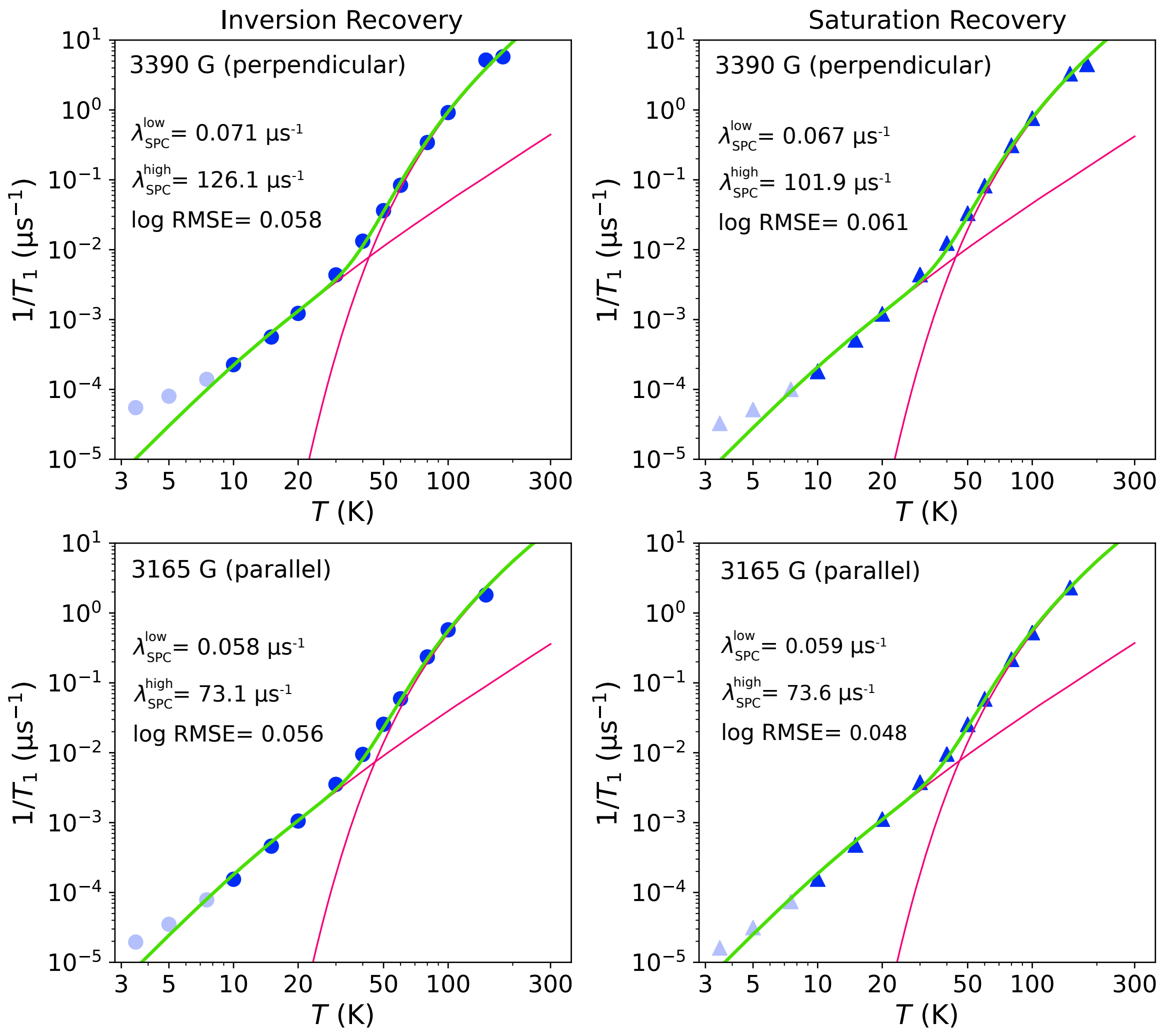

Figure S49. *SPC fits for CuPc at different field positions, collected using inversion-recovery (o) and saturation-recovery (Δ) pulse sequences. Pink curves show individual contributions from* λ_{SPC}^{low} *and* λ_{SPC}^{high}*, and green curves show the total fits.*

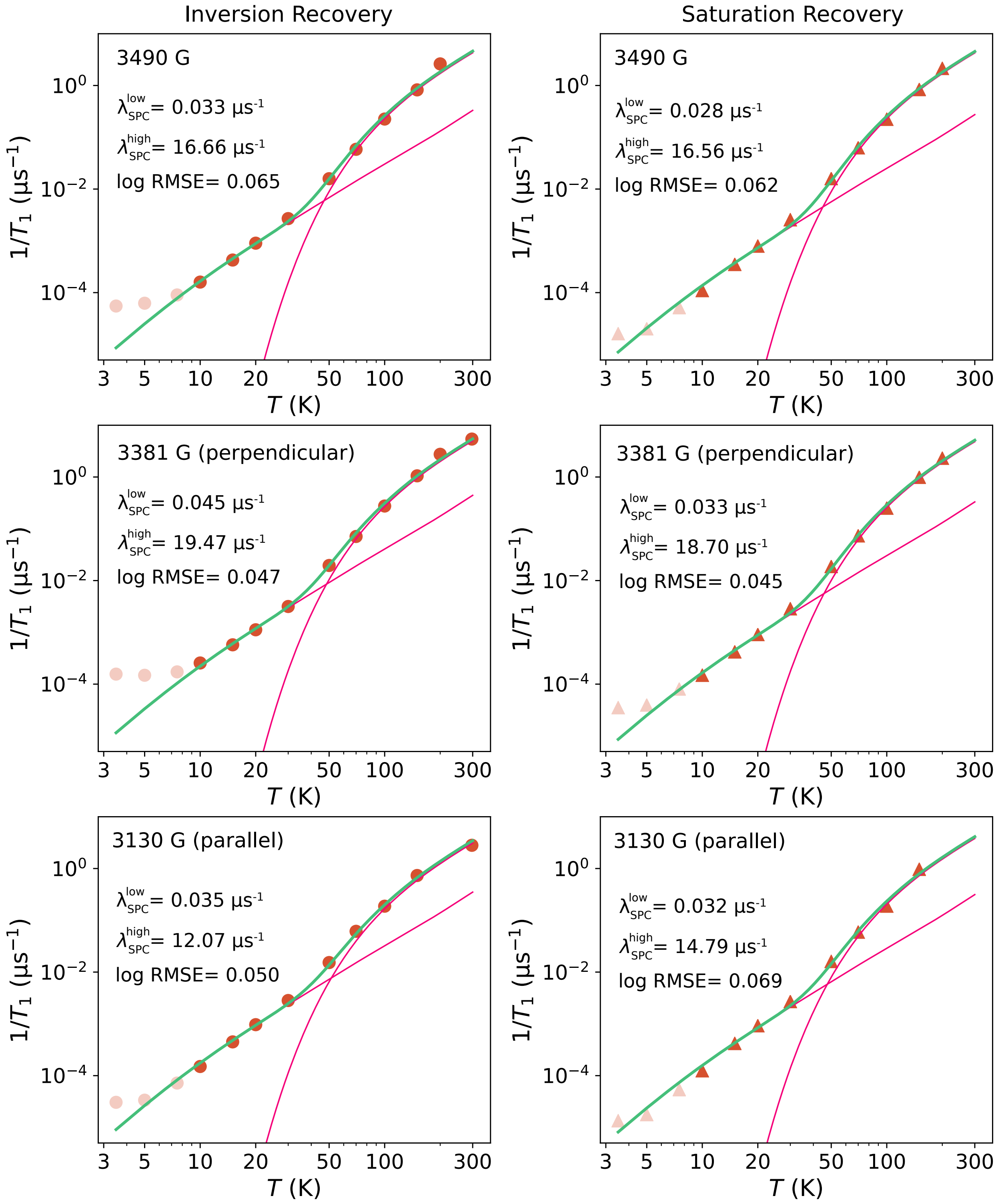

Figure S50. *SPC fits for CuOEP at different field positions, collected using inversion-recovery (o) and saturation-recovery (Δ) pulse sequences. Pink curves show individual contributions from* λ_{SPC}^{low} *and* λ_{SPC}^{high}*, and green curves show the total fits. Only data above 10 K were included, as the direct relaxation mechanism becomes important at lower temperatures.*

7.4 Smooth Crossover Description of $\lambda_{SPC}(E)$

The fits presented in the main manuscript assume an abrupt change in the SPC parameter at a transition energy between λ_{SPC}^{low} and λ_{SPC}^{high}. While this piecewise description minimizes the number of free parameters, such a discontinuous change is not physically realistic. To introduce a smooth crossover between the low- and high-energy regimes, we alternatively model the SPC coefficient using a sigmoidal function of the form:

$$\lambda_{SPC}(E) = \lambda_{SPC}^{low} + \frac{\lambda_{SPC}^{high} - \lambda_{SPC}^{low}}{1 + \exp[-k(E - E_{trans})]}$$

Here, λ_{SPC}^{low} and λ_{SPC}^{high} represent the low- and high-energy asymptotic limits, respectively, while E_{trans} defines the midpoint of the transition and k is the new parameter that controls its sharpness (the characteristic transition width is given by $1/k$). **Figure S51** shows the corresponding fits for CuPc and CuOEP.

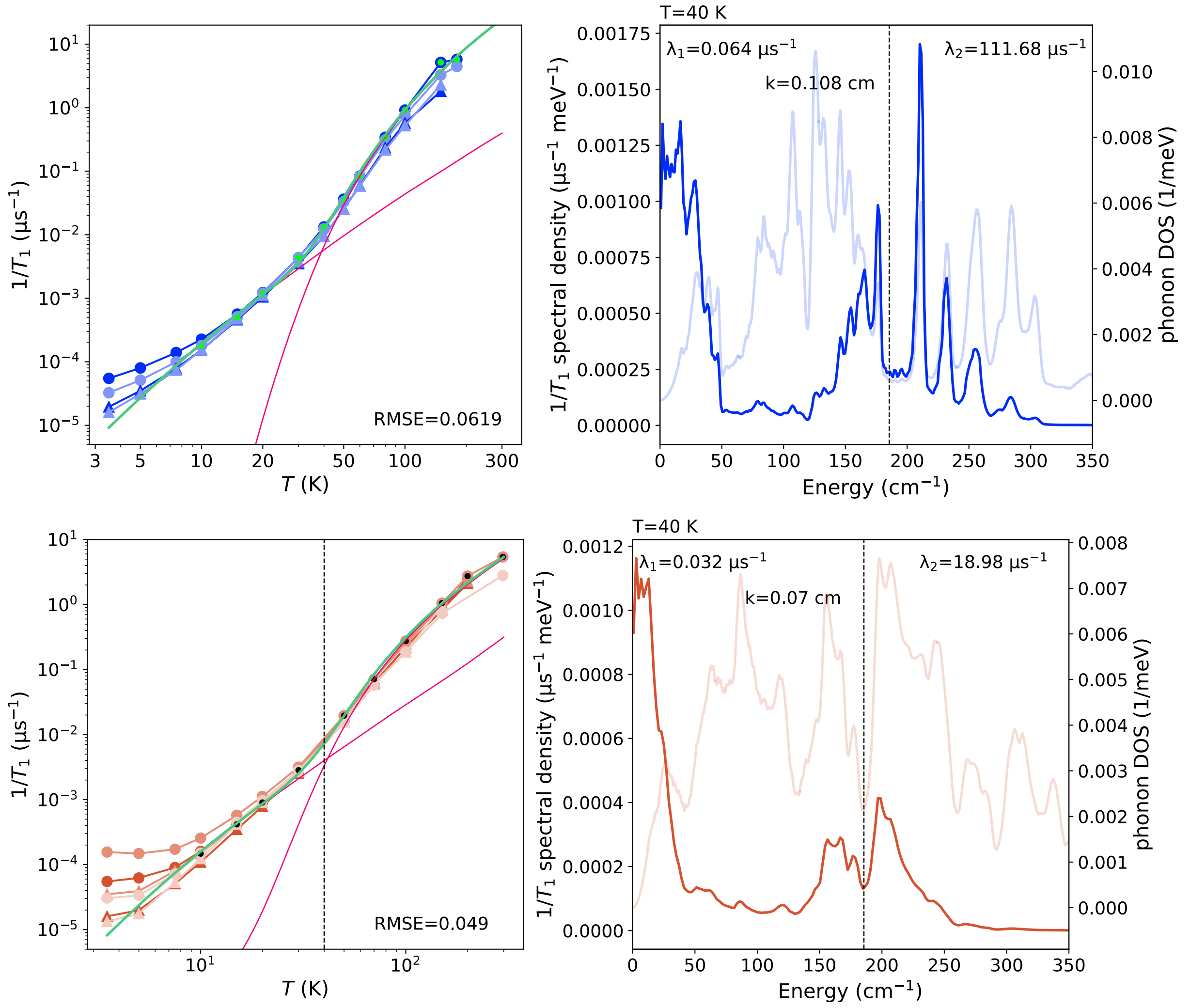

Figure S51. *SPC fits with a sigmoid description for $\lambda_{SPC}(E)$* . Top: *CuPc.* Bottom: *CuOEP.* Left: *T_1 values measured by EPR at different fields using both inversion-recovery and saturation-recovery pulse sequences. Green curves show the*

full fit, and pink curves show the individual contributions from λ_1 (=λ_{SPC}^{low}), and λ_2 (=λ_{SPC}^{high}). Marked data points (green for CuPc and black for CuOEP) were included in the fit. Right: *$1/T_1$ spectral density at 40 K overlaid with the phonon density of states (light shaded curve). The fitted SPC coefficients are shown. Dashed lines indicate the energy transition E_{trans}.*

7.5 Including a third window

A third energy window between 50 and 185 cm^{-1} was added to isolate the contribution of intermediate-energy modes. As discussed in the main text, vibrations in this range do not appear to contribute significantly to relaxation but were isolated here to evaluate their contribution explicitly. As shown in **Figure S52**, including the third window causes the SPC coefficients for modes below 50 cm^{-1} and above 185 cm^{-1} to decrease slightly relative to the two-window fit. The SPC coefficients for modes within the new 50–185 cm^{-1} window are larger than those below 50 cm^{-1}. However, because these modes are much less thermally populated, their overall contribution to $1/T_1$ remains smaller than that of both the low- and high-energy regions, and they never dominate the spin relaxation.

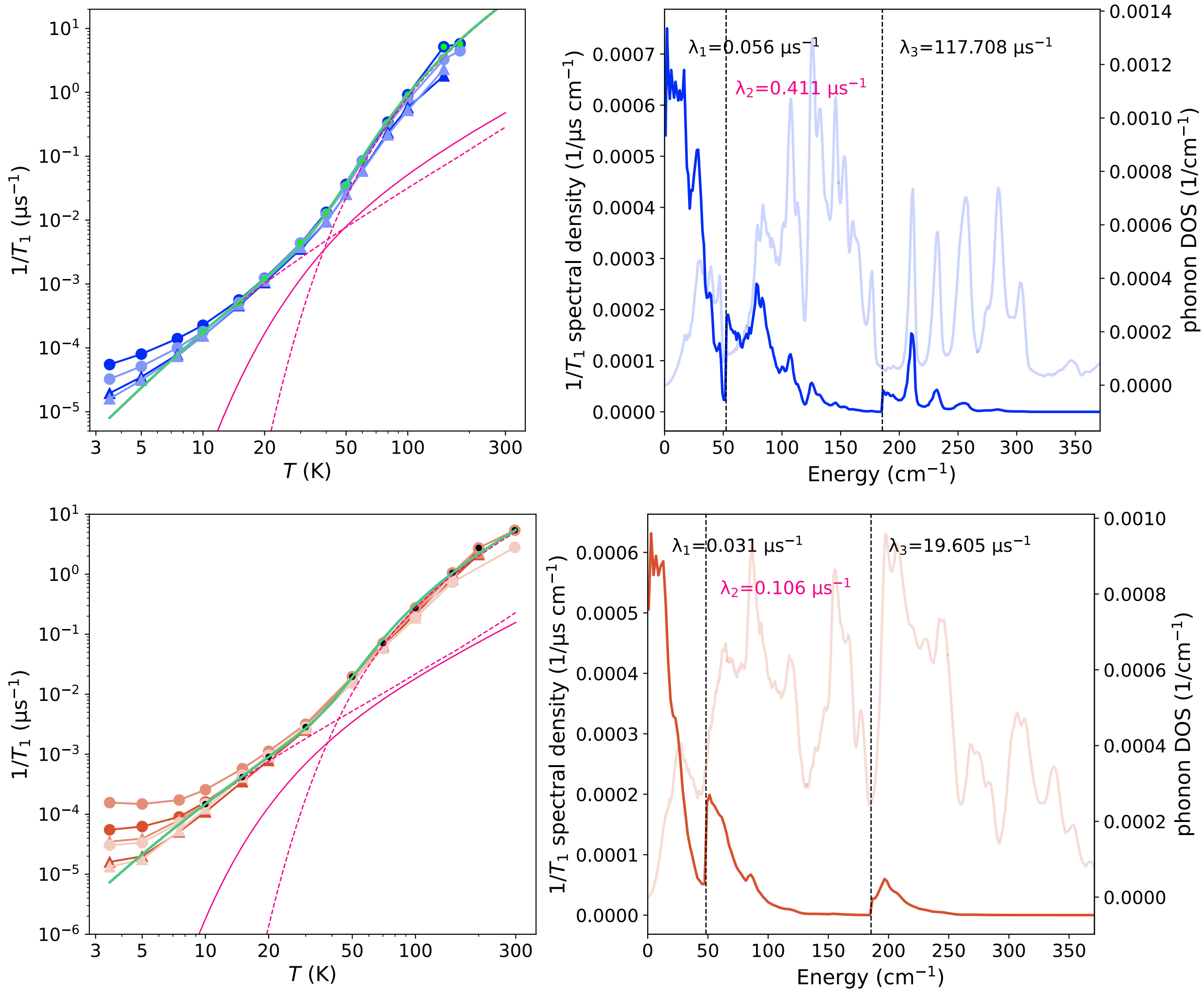

Figure S52. *SPC fits using three energy windows.* Top: *CuPc.* Bottom: *CuOEP.* Left: *T_1 values measured by EPR at different fields using both inversion-recovery and saturation-recovery pulse sequences. Green curves show the full fit,*

and pink curves show the individual contributions from λ_1, λ_2, and λ_3. Marked data points (green for CuPc and black for CuOEP) were included in the fit. The fit RMSE in log-space is 0.051 for CuPc and 0.043 for CuOEP. Right: *$1/T_1$ spectral density at 30 K overlaid with the phonon density of states (light shaded curve). The fitted SPC coefficients are listed for each energy window. Dashed lines indicate the energy thresholds separating the three fitting regions.*

If only phonon energies above 15 cm^{-1} are considered, this trend becomes clearer as shown in **Figure S53** (acoustic modes below 15 cm^{-1} are mostly translational modes that cannot directly couple with spins if acoustic-optical mixing is ignored). For CuPc, the fitted SPC coefficient for modes between 50 and 185 cm^{-1} becomes essentially the same as that of the low-energy modes below 50 cm^{-1}. Due to their lower population, phonons in the intermediate-energy window contribute much less to relaxation than vibrations in the other regions at all temperatures, validating the discussion in the main text. Strikingly, for CuOEP, the SPC strength for phonons in the intermediate-energy range approaches zero ($\lambda = 8 \times 10^{-11}$ μs^{-1}), demonstrating that these modes contribute negligibly to spin relaxation.

These fits, which include a third intermediate-energy range, reinforce our interpretation that modes between 50 and 185 cm^{-1} play only a minor role in spin relaxation. They also justify the use of two energy windows for the final fits: when the mid-range phonons are combined with the low-energy window (with the same SPC coefficient), they contribute less due to their low thermal occupation. Such an approach is consistent with the T_1 data measured for both CuPc and CuOEP, which exhibit a clear single slope change around 40 K.

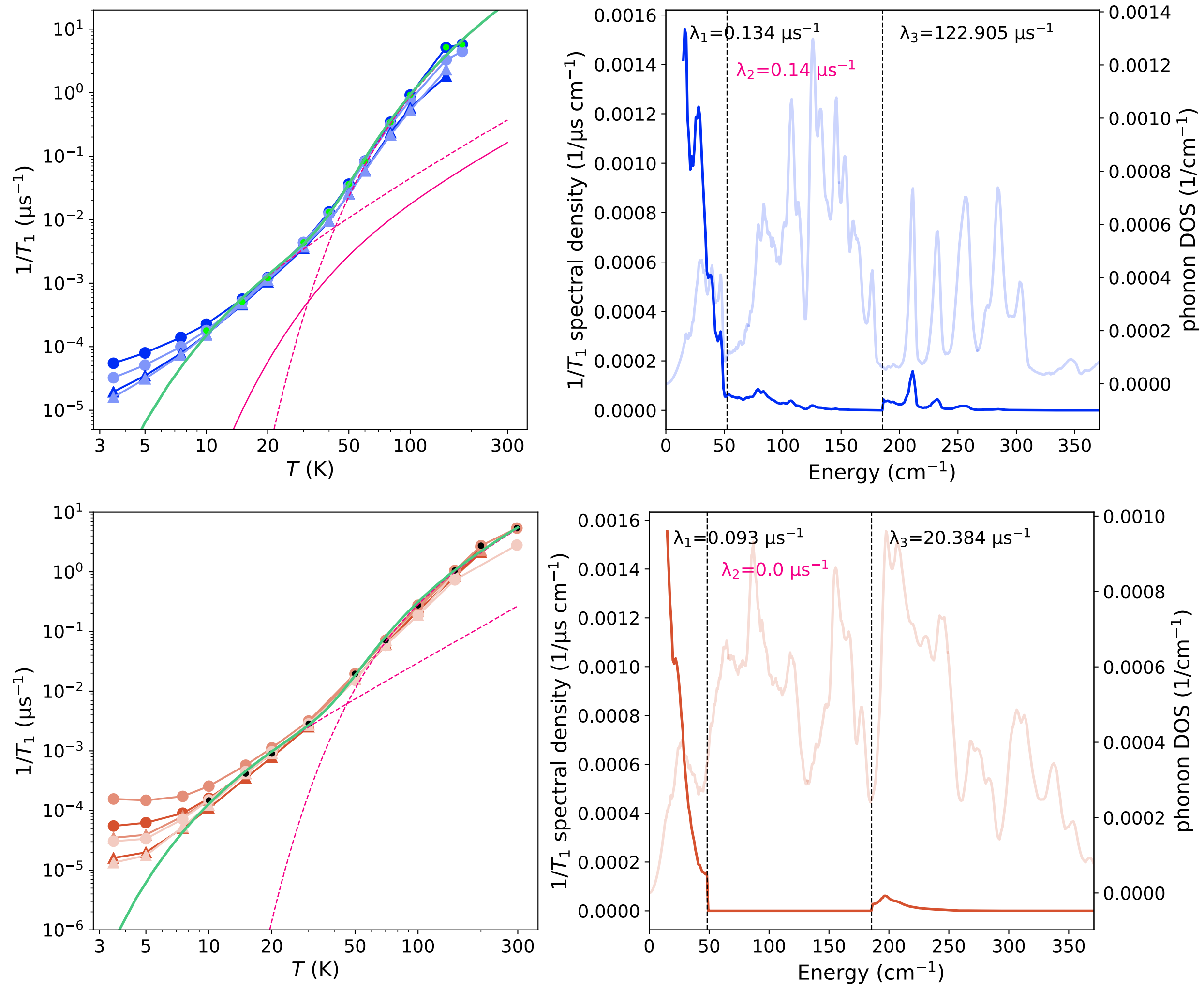

Figure S53. *SPC fits using three energy windows while excluding phonon modes below 15 cm^{-1}.* Top: *CuPc.* Bottom: *CuOEP.* Left: *T_1 values measured by EPR at different fields using both inversion-recovery and saturation-recovery pulse sequences. Green curves show the full fit, and pink curves show the individual contributions from λ_1, λ_2, and λ_3. Marked data points (green for CuPc and black for CuOEP) were included in the fit. The fit RMSE in log-space is 0.058 for CuPc and 0.048 for CuOEP.* Right: *$1/T_1$ spectral density at 30 K overlaid with the phonon density of states (light shaded curve). The fitted SPC coefficients are listed for each energy window. Dashed lines indicate the energy thresholds separating the three fitting regions.*

8 Phonon calculations

Spin-polarized density functional theory (DFT) calculations were carried out using the Vienna *Ab initio* Simulation Package (VASP)(15). The projector augmented-wave (PAW) method (16, 17) was employed to describe the core–electron interactions, with a plane-wave energy cutoff of 800 eV for the valence electrons. The lattice parameters and atomic coordinates reported in the literature (2, 4) were used as the initial structural model. The CuOEP unit cell contained 85 atoms, including one Cu atom, while the CuPc unit cell contained 114 atoms, including two Cu atoms. A Hubbard U correction of 4.0 eV was applied to account for the localized Cu 3d electrons. While coupling between magnetic moments of Cu(II) is weak in this case, anti-ferromagnetic (AFM) configuration was found to have slightly lower potential energy. A 1 × 1 × 2 supercell of CuOEP was thus created as the magnetic unit cell, with the two Cu(II) having anti-parallel spin polarization. For CuPc, the two Cu(II) in the unit cell were assigned to have AFM configuration.

The electronic structures were computed using a Γ-centered *k*-point mesh of 2 × 2 × 3 for CuOEP and 2 × 7 × 3 for CuPc. The convergence criteria for electronic and ionic relaxations were set to 10^{-8} eV and 10^{-7} eV, respectively. After the unit cells were fully relaxed, the maximum residual interatomic force was below 0.001 eV $Å^{-1}$. Dispersion interactions were treated using the optB86b-vdW functional (18, 19).

Second derivatives of the potential energy with respect to the atomic coordinates were obtained using the magnetic unit cell of CuOEP and a 1 × 2 × 1 supercell for CuPc. The vibrational eigenfrequencies and modes were subsequently calculated using *Phonopy (20)*. The DFT-calculated phonon results were then converted to simulated inelastic neutron scattering (INS) spectra using the *OCLIMAX* software (21).

8.1 Mean-squared displacements

Root-mean-squared displacements (RMSDs) were computed from harmonic phonon eigenvectors following the formulation implemented in Phonopy *(19, 21)*. For each vibrational mode m with frequency ω_m, the thermally averaged mean-square displacement of atom j was calculated as

$$\langle u_j^2 \rangle = \frac{\hbar}{2M_j\omega_m}(2n_m(T)+1)\left|e_{j,m}\right|^2$$

where M_j is the atomic mass, $e_{j,m}$ is the phonon eigenvector component of atom j, and $n_m(T)$ is the Bose–Einstein occupation factor at temperature *T*. Frequencies were converted to angular units, and all quantities were evaluated at *T* = 300 K.

The molecular RMSD was obtained by summing the mean-square displacements over the phonon modes (0–400 cm^{-1} and 185–300 cm^{-1}) and averaging over all atoms in the molecule,

yielding a per-atom isotropic RMSD. Core RMSDs were computed analogously but were restricted to the Cu atom and its four first-coordination N atoms. For both quantities, RMSDs were evaluated independently for the two crystallographically equivalent molecules in the unit cell and subsequently averaged. Results of per-atom RMSDs are presented in **Table S8**.

Energy window	Quantity	CuPc RMSD (Å)	CuOEP RMSD (Å)
0 – 400 cm^{-1}	Molecule RMSD	2.4376e-01	3.2084e-01
	Cu-N core RMSD	1.7411e-01	1.5243e-01
185 – 300 cm^{-1}	Molecule RMSD	5.7170e-02	1.1946e-01
	Cu-N core RMSD	6.6331e-02	6.6463e-02

Table S8. *Per-atom root-mean-squared displacements (RMSDs) of CuPc and CuOEP for the full molecule and the Cu–N coordination core, obtained by summing vibrational contributions over two distinct phonon energy windows.*

8.2 Stretching character of modes

The stretching character of vibrational modes involving the Cu–N coordination core was quantified directly from the phonon eigenvectors. For each Cu–N core, the four Cu–N bond unit vectors $\hat{b}_i$ were defined from the equilibrium structure using the shortest Cu–N distances under periodic boundary conditions. A local coordination plane was determined for each core by fitting a plane through the four N atoms, and all displacements were projected onto this plane to remove out-of-plane contributions.

For a given vibrational mode m, the in-plane displacement of each N atom, $u^{\parallel}_{N_i,m}$, was projected onto its corresponding Cu–N bond direction to obtain a radial stretching component $s_i = u^{\parallel}_{N_i,m} \cdot \hat{b}_i$. The degree of phase coherence between the four N displacements was quantified by an in-phase metric, $C_{phase} = |\sum_i s_i| / \sum_i |s_i|$, which equals 1 for perfectly in-phase (symmetric) stretching and approaches 0 for completely out-of-phase motion. The overall stretching strength of the mode was defined as $S_{stretch} = \sum_i s_i^2$, which measures the magnitude of radial Cu–N distortions independent of phase. The final stretching score for each core and mode was defined as the product $S_{stretch} \cdot C_{phase}$. Stretching scores were computed independently for the two Cu–N cores in the unit cell and subsequently averaged to obtain a single value per vibrational mode. This procedure yields a continuous, symmetry-agnostic ranking of vibrational modes according to their Cu–N symmetric stretching character and is robust to structural distortions and symmetry lowering.

The phonon modes were ranked by their stretching score. The top 20 modes and their corresponding scores are listed in **Table S9** and **Table S10**. In both systems, pairs of nearly degenerate modes are observed, reflecting the presence of two molecules per unit cell/ supercell. These pairs correspond to the same underlying vibrational motion, with small frequency splittings due to subtle structural asymmetries.

Rank	Frequency (cm^{-1})	Stretch Score
1	255.86	2.703390e-02
2	256.70	2.141357e-02
3	254.30	7.474024e-03
4	259.34	2.380107e-03
5	165.77	4.234526e-04
6	173.31	4.205230e-04
7	176.12	2.990811e-04
8	230.83	2.836578e-04
9	179.49	2.505402e-04
10	257.33	2.096486e-04
11	263.66	1.998487e-04
12	285.24	1.498015e-04
13	148.07	1.475676e-04
14	228.18	1.160499e-04
15	151.07	1.104611e-04
16	289.47	1.085934e-04
17	130.90	5.391739e-05
18	116.54	3.928659e-05
19	127.72	3.630205e-05
20	84.37	3.238191e-05

Table S9. *Phonon modes of CuPc ranked by their stretching character (the stretching score is defined in text).*

Rank	Frequency (cm^{-1})	Stretch Score
1	347.88	3.053113e-02
2	352.31	3.030369e-02
3	267.89	1.779256e-02
4	267.85	9.011818e-03
5	287.70	6.418883e-03
6	220.42	4.381592e-03
7	259.05	3.350064e-03
8	217.28	3.264049e-03
9	292.29	3.242908e-03
10	240.20	3.231435e-03
11	203.40	2.872381e-03
12	183.24	2.762012e-03
13	320.68	2.274311e-03
14	194.38	2.091520e-03
15	198.84	2.036775e-03
16	216.17	1.379839e-03
17	211.07	1.202342e-03
18	324.43	1.156709e-03

19	166.56	9.809366e-04
20	288.70	9.293994e-04

Table S10. *Phonon modes of CuOEP ranked by their stretching character (the stretching score is defined in text).*

9 References

1. H. Ogata, *et al.*, Absorption, Magnetic Circular Dichroism, IR Spectra, Electrochemistry, and Molecular Orbital Calculations of Monoaza- and Opposite Diazaporphyrins. *Eur. J. Inorg. Chem.* (2004).

2. H. Jiang, *et al.*, Hole Mobility Modulation in Single-Crystal Metal Phthalocyanines by Changing the Metal–π/π–π Interactions. *Angew Chem Int Ed* **57**, 10112–10117 (2018).

3. D. Li, *et al.*, Green synthesis and characterization of crystalline zinc phthalocyanine and cobalt phthalocyanine prisms by a simple solvothermal route. *CrystEngComm* **20**, 2749–2758 (2018).

4. R. Pak, W. R. Scheidt, Structure of (2,3,7,8,12,13,17,18-octaethylporphinato)copper(II). *Acta Crystallogr. Sec. C* **47**, 431–433 (1991).

5. A. Ozarowski, H. M. Lee, A. L. Balch, Crystal Environments Probed by EPR Spectroscopy. Variations in the EPR Spectra of CoII (octaethylporphyrin) Doped in Crystalline Diamagnetic Hosts and a Reassessment of the Electronic Structure of Four-Coordinate Cobalt(II). *J. Am. Chem. Soc.* **125**, 12606–12614 (2003).

6. N. P. Kazmierczak, N. E. Lopez, K. M. Luedecke, R. G. Hadt, Determining the key vibrations for spin relaxation in ruffled Cu(II) porphyrins *via* resonance Raman spectroscopy. *Chem. Sci.* **15**, 2380–2390 (2024).

7. N. P. Kazmierczak, R. G. Hadt, Illuminating Ligand Field Contributions to Molecular Qubit Spin Relaxation via T_1 Anisotropy. *J. Am. Chem. Soc.* **144**, 20804–20814 (2022).

8. Z. Xue, *et al.*, Neutron Instruments for Research in Coordination Chemistry. *Eur J Inorg Chem* **2019**, 1065–1089 (2019).

9. A. Ramirez-Cuesta, R. Balderas Xicohtencatl, Y. Cheng, Inelastic neutron scattering: A unique tool to study hydrogen in materials. *Journal of Materials Research* **39**, 727–736 (2024).

10. O. Arnold, *et al.*, Mantid—Data analysis and visualization package for neutron scattering and μ SR experiments. *Nuclear Instruments and Methods in Physics Research Section A: Accelerators, Spectrometers, Detectors and Associated Equipment* **764**, 156–166 (2014).

11. E. Garlatti, *et al.*, The critical role of ultra-low-energy vibrations in the relaxation dynamics of molecular qubits. *Nat Commun* **14**, 1653 (2023).

12. G. L. Squires, *Introduction to the theory of thermal neutron scattering*, 3rd ed (Cambridge university press, 2012).

13. L. Mauger, S. H. Lohaus, B. Fultz, The temperature dependence of nuclear resonant X-Ray spectra of magnetic iron and cementite. *Hyperfine Interact* **243**, 5 (2022).

14. L. Mauger, *et al.*, Nonharmonic phonons in α-iron at high temperatures. *PHYSICAL REVIEW B* **90**, 064303–064303 (2014).

15. G. Kresse, J. Furthmüller, Efficient iterative schemes for *ab initio* total-energy calculations using a plane-wave basis set. *Phys. Rev. B* **54**, 11169–11186 (1996).

16. P. E. Blöchl, Projector augmented-wave method. *Phys. Rev. B* **50**, 17953–17979 (1994).

17. G. Kresse, D. Joubert, From ultrasoft pseudopotentials to the projector augmented-wave method. *Phys. Rev. B* **59**, 1758–1775 (1999).

18. J. P. Perdew, K. Burke, M. Ernzerhof, Generalized Gradient Approximation Made Simple. *Phys. Rev. Lett.* **77**, 3865–3868 (1996).

19. J. Klimeš, D. R. Bowler, A. Michaelides, Chemical accuracy for the van der Waals density functional. *J. Phys.: Condens. Matter* **22**, 022201 (2010).

20. A. Togo, I. Tanaka, First principles phonon calculations in materials science. *Scripta Materialia* **108**, 1–5 (2015).

21. Y. Q. Cheng, L. L. Daemen, A. I. Kolesnikov, A. J. Ramirez-Cuesta, Simulation of Inelastic Neutron Scattering Spectra Using OCLIMAX. *J. Chem. Theory Comput.* **15**, 1974–1982 (2019).

21. Phonopy documentation, *Thermal displacement*, available at https://phonopy.github.io/phonopy/formulation.html#mean-square-displacement (accessed December 2025).
